# Evolution and Allometry of Calcaneal Elongation in Living and Extinct Primates

**DOI:** 10.1371/journal.pone.0067792

**Published:** 2013-07-03

**Authors:** Doug M. Boyer, Erik R. Seiffert, Justin T. Gladman, Jonathan I. Bloch

**Affiliations:** 1 Department of Evolutionary Anthropology, Duke University, Durham, North Carolina, United States of America; 2 Department of Anthropology, City University of New York Graduate Center, New York, New York, United States of America; 3 New York Consortium in Evolutionary Primatology, New York, New York, United States of America; 4 Department of Anatomical Sciences, Stony Brook University, Stony Brook, New York, United States of America; 5 Florida Museum of Natural History, University of Florida, Gainesville, Florida, United States of America; Monash University, Australia

## Abstract

Specialized acrobatic leaping has been recognized as a key adaptive trait tied to the origin and subsequent radiation of euprimates based on its observed frequency in extant primates and inferred frequency in extinct early euprimates. Hypothesized skeletal correlates include elongated tarsal elements, which would be expected to aid leaping by allowing for increased rates and durations of propulsive acceleration at takeoff. Alternatively, authors of a recent study argued that pronounced distal calcaneal elongation of euprimates (compared to other mammalian taxa) was related primarily to specialized pedal grasping. Testing for correlations between calcaneal elongation and leaping versus grasping is complicated by body size differences and associated allometric affects. We re-assess allometric constraints on, and the functional significance of, calcaneal elongation using phylogenetic comparative methods, and present an evolutionary hypothesis for the evolution of calcaneal elongation in primates using a Bayesian approach to ancestral state reconstruction (ASR). Results show that among all primates, logged ratios of distal calcaneal length to total calcaneal length are inversely correlated with logged body mass proxies derived from the area of the calcaneal facet for the cuboid. Results from phylogenetic ANOVA on residuals from this allometric line suggest that deviations are explained by degree of leaping specialization in prosimians, but not anthropoids. Results from ASR suggest that non-allometric increases in calcaneal elongation began in the primate stem lineage and continued independently in haplorhines and strepsirrhines. Anthropoid and lorisid lineages show stasis and decreasing elongation, respectively. Initial increases in calcaneal elongation in primate evolution may be related to either development of hallucal-grasping or a combination of grasping and more specialized leaping behaviors. As has been previously suggested, subsequent increases in calcaneal elongation are likely adaptations for more effective acrobatic leaping, highlighting the importance of this behavior in early euprimate evolution.

## Introduction

**Figure 1:**
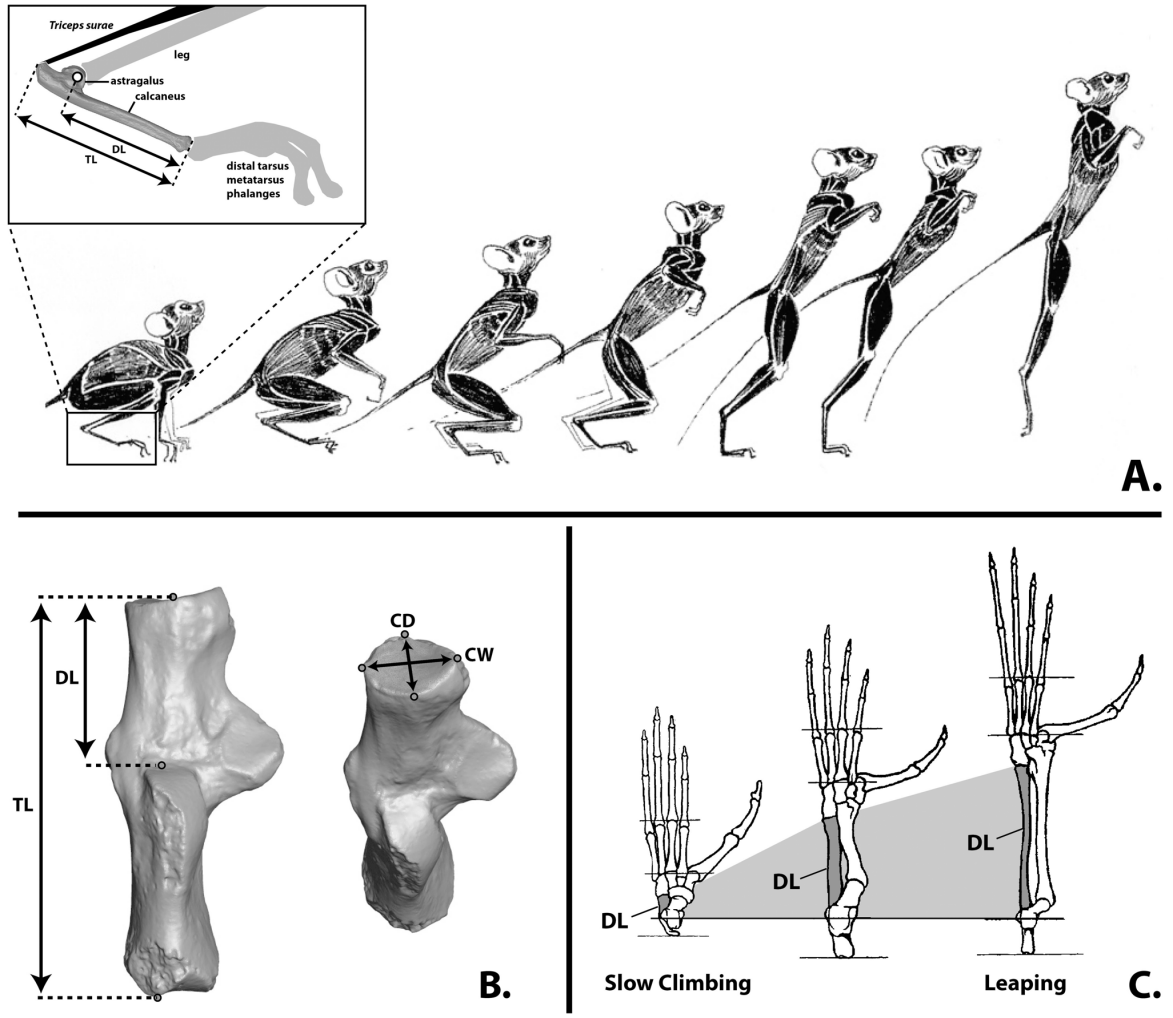
The biomechanical role of the ankle in leaping with a tarsifulcrumating foot. **A**, Incremental stages in hind limb extension that accelerates the center of mass in a largely vertical direction in order to produce inertia that carries the animal through the air after the limbs are fully extended. The inset shows the relationship of distal segment (DL) of the calcaneus to the rest of the foot: it forms the “load arm” in a *class 2* lever system. The lever arm (the heel) comprises the rest of the calcaneal length (TL). **B**, Measurements used in this study shown on a left calcaneus. Abbreviations: CD, cuboid facet depth; CW, cuboid facet width; TL, total proximodistal length; DL, distal segment length. **C**, Left feet of primates exhibiting different degrees of leaping specialization scaled to same metatarsus length and aligned at fulcrum of ankle. Taxa that never use leaping behavior have much shorter tarsal bones as shown on the left. The way in which differential degrees of leaping specialization and body-size interact to influence and complicate this relationship is debated [7].

Extant primates are unusual among mammals in having relatively large brains, large forward facing eyes with high visual acuity, and hands and feet that are specialized for grasping [1], [2]. Additionaly, many strepsirrhine, tarsiers and certain platyrrhine primates are also unique among mammals in their “grasp-leaping” locomotion [3]. This arboreal behavior is characterized by the use of grasping feet to anchor on a horizontal or vertical support while the hind limbs extend and accelerate the body in a direction that has some vertical component. As the hind limbs reach full extension and the support is released, the body motion is ballistic. Importantly, termination of the leap involves relatively precise “grasping” of the support on landing. Theoretically, such precise grasping requires quick reflexes and exceptional eye-hand coordination [4] (Fig. 1). The “Vertical Clinging and Leaping” (VCL) locomotor mode in primates involves preferential use of “vertical supports” and in some cases more acrobatically specialized leaping styles [5], [6], but is otherwise similar to “grasp-leaping.”

While key morphological correlates of grasp-leaping are debated [7], grasp-leaping behaviors are nonetheless often inferred as having been present in the common ancestor of living primates [3], [8], [9], and are regularly implicated as a driving influence in the early adaptive radiation of euprimates [3], [8], [9]. If this is correct, then selection for improvements in leaping performance may explain the evolution of certain euprimate characteristics, even those that are not directly related to generating acceleration for a leap. Even forward facing eyes might have evolved as part of an adaptive suite that allowed improved rapid and acrobatic negotiation of an arboreal setting. On the other hand, if leaping behaviors were not important to the ancestral modern primate, as suggested by researchers who have used marsupial analogies to study primate origins [10], [11], then visual features must have evolved for a biological role unrelated to leaping. A major alternative idea for the adaptive significance of euprimate features is the nocturnal visual predation hypothesis [12], [13], which has received mixed support over the years [14]–[16]. In the most recent explanation of this hypothesis [17] it is proposed that at least the unusually specialized features of the visual system and associated skull features, including a postorbital bar, orbital convergence, and frontated orbits, arose in response to a selective pressure favouring the visual detection and stealthy capture of insects at night. This idea has been supported by comparative data showing that among extant groups of closely related animals, species that are nocturnal visual predators have greater orbital convergence [14]. Additionally, vertebrates with the most convergent orbits tend to be predators (e.g., raptorial birds) [18].

The fossil record provides the only direct evidence to evaluate whether visual and leaping features arose at the same time in primate evolution. Fossil stem primates (“plesiadapiforms”) are a diverse group [19] that first appear at the beginning of the Paleogene and reach their greatest diversity prior to the appearance of any fossils likely to represent crown primates or “primates of modern aspect” (Euprimates [20]). All known “plesiadapiforms” appear to lack certain key features characteristic of euprimates (e.g., a postorbital bar, orbital convergence, and flattened nails on the non-hallucal digits) while other euprimate features are present in at least some “plesiadapiforms” (e.g., prehensile proportions of the hands and feet, a mobile forearm, and a divergent, opposable hallux) [15], [16], [18]. Though current perceptions of cranial and postcranial diversity in plesiadapiforms are tempered knowing that very few skulls or skeletons have been recovered relative to “plesiadapiform” taxonomic and dental diversity, the available stem primate fossil record strongly suggests that postcranial features for grasping and locomotion in a fine-branch niche preceeded visual and leaping specializations [15], [16], [19]. In contrast, even the oldest and most dentally primitive euprimates known from both cranial and postcranial morphology (species of *Teilhardina*) have a postorbital bar and orbital convergence [21] and have been argued by some authors to exhibit leaping specializations [5], [22], [23]. However, because *Teilhardina* is still potentially somewhat removed from the “euprimate ancestor,” and because debate remains [7] about how to interpret leaping behaviors from bones, the fossil record remains ambiguous as to the timing of acquisition of primate-like visual specializations relative to postcranial features that may relate to leaping.

Advances in statistical methods that use data from extant and fossil taxa in conjunction with a specified phylogenetic tree (or sample of trees) to estimate morphological and behavioral trait values in ancestral taxa [24] provide the potential for more rigorously supported hypotheses regarding the role of leaping in primate origins. To produce meaningful results such an approach should maximize inclusion of taxonomic diversity [24]. Dozens of fossil species known from dental remains give a small glimpse into ancient euprimate diversity [25]–[27]. Unfortunately, among aspects of the skeleton correlated with locomotor behavior, few are preserved with any degree of comprehensiveness.

**Figure 2:**
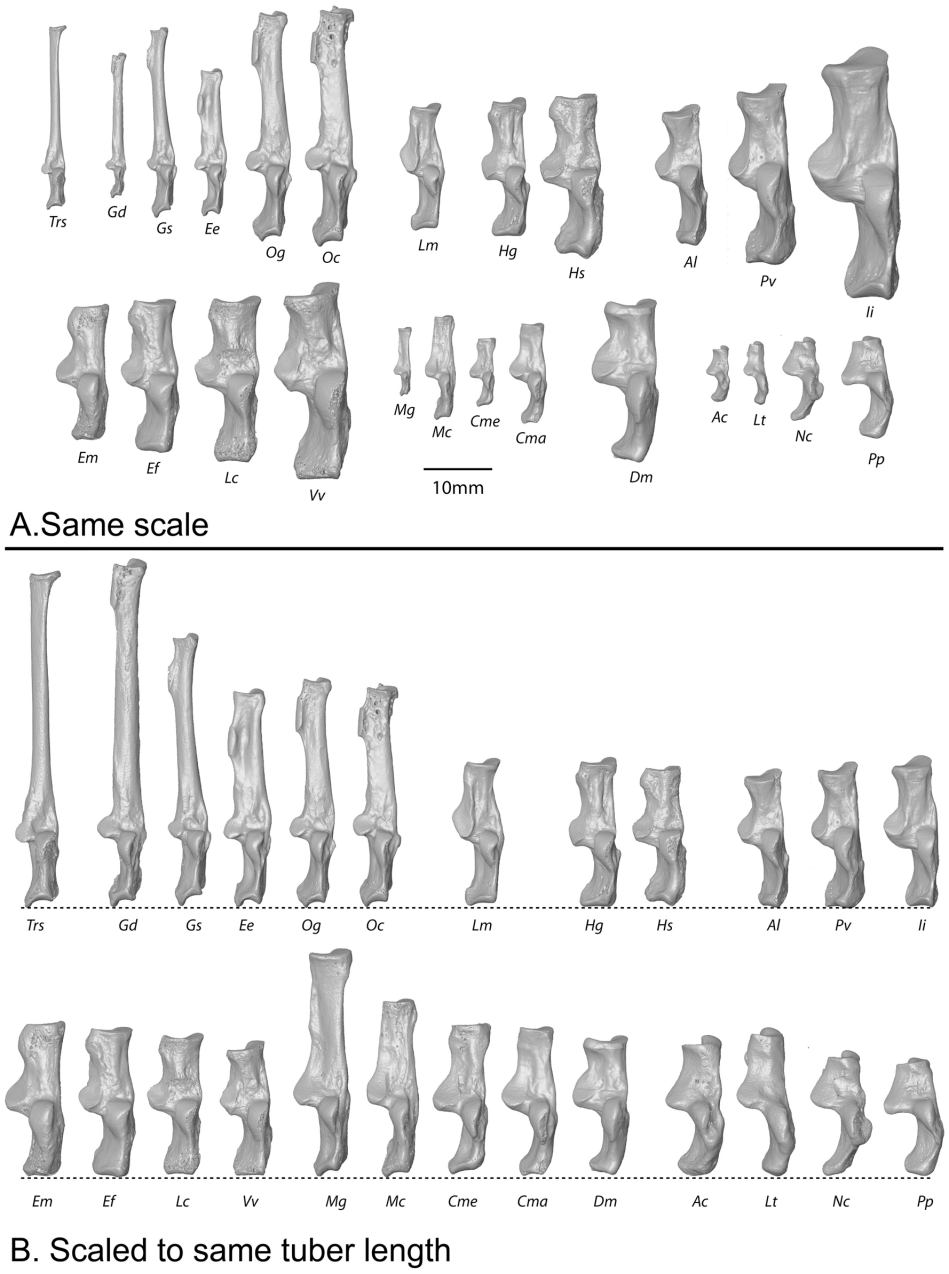
Extant prosimian calcanei exhibit a diversity of sizes and proportions. **A**, Almost all major prosimian genera are represented at the same scale. **B**, The same taxa are represented, scaled to length of the proximal segment and arranged (within familial groups) so that the smallest members are on the left, while the largest are on the right. This organization helps one visualize qualitatively, the allometric trends plotted in subsequent figures. Abbreviations: Ac, *Arctocebus calabarensis*; Al, *Avahi laniger*; Cma, *Cheirogaleus major*; Cme, *Cheirogaleus medius*; Dm, *Daubentonia madagascariensis*; Ee, *Euoticus elegantulus*; Ef, *Eulemur fulvus*; Em, *Eulemur mongoz*; Gd, *Galagoides demidovii*; Gs, *Galago senegalensis*; Hg, *Hapalemur griseus*; Hs, *Hapalemur simus*; Ii, *Indri indri*; Lc, *Lemur catta;* Lm, *Lepilemur mustelinus*; Lt, *Loris tardigradus*; Mc, *Mirza coquereli*; Mg, *Microcebus griseorufus*; Nc, *Nycticebus coucang*; Oc, *Otolemur crassicaudatus*; Og, *Otolemur garnetti*; Pp, *Perodicticus potto*; Pv, *Propithecus verreauxi*; Vv, *Varecia variegata*.

Because the form of the tarsals is both relatively well known for early fossil euprimates and reflects functional attributes of the foot that vary with behavior [7], [28]–[30], studies focusing on the tarsus have relatively good potential to help address questions about locomotion and positional behavior in the early evolution of primates. While the relationship between calcaneal form and leaping can be complicated by strong allometric affects [7], [31]–[35], it has been suggested that among small taxa an elongated distal segment of the calcaneus reflects proclivity for acrobatic leaping [29], [30]. Extreme calcaneal elongation in small taxa is correlated with the specialized niche of Vertical Clinging and Leaping (VCL) [5]. On the other hand, there is not a clear signature of elongation that signifies leaping when taxa of very different body sizes are considered: The largest specialized leapers (i.e., extant Indriidae), have calcanei with absolute degrees of elongation that are virtually identical to those of small taxa [7], [33] (Fig. 2). This clearly equates to substantially less elongation relative to body mass in these large taxa. This situation complicates the use of elongation as a proxy for leaping ability: the absolute length of the calcaneus and its segments increase with body size among leapers going from tarsiers and small galagos to large galagos and then decreases when considering still larger taxa such as *Prolemur simus* and some indriids. This has led to the proposition (also supported by sound biomechanical reasoning) that with increasing body size, the distal limb segment gives up its role in acceleration production [5], [32], [33]. However, as noted 30 years ago by Matt Cartmill [12], *Lepilemur* and *Hapalemur griseus* are similar in mass to *Otolemur crassicaudatus* and appear to rely on leaping to an even greater degree [5], but have much less absolute and relative elongation (Fig. 2). Recognition of these phenomena in the literature is also reflected by the statement of Dagosto et al. [36], that “no features of the calcaneus clearly distinguish extant leaper/quadrupeds from VCLs” (p.196). Even in the face of this complexity and related ambiguities, phenetic similarities are still interpreted by some as reflective of locomotor equivalence regardless of body size [37].

It has even been suggested that, with the exception of the case of small-bodied vertical clingers and leapers, there is no relationship between leaping and calcaneal elongation [7]. Thus, calcaneal elongation would have evolved almost solely to accommodate the loss of foot leverage that occurred in the acquisition of a mobile, grasping-specialized hallucal metatarsal, which shifts the fulcrum of the distal limb segment from the metatarsal heads to the tarso-metatarsal joint [29]. A comprehensive allometric analysis of calcaneal elongation by Moyà-Solà et al. [7] showed that euprimates have a distal calcaneal segment which, when corrected for body size differences, is longer than that of most non-primate mammals. They argued that because this also applies for non-leaping primates (e.g., lorises, orangutans, howler monkeys), calcaneal elongation among primates relative to other mammals is not explained by unique leaping abilities. In addition, lack of a leaping “signal” in calcaneal distal elongation was further demonstrated by the finding that leaping taxa such as indriids do not exhibit a relatively longer distal segment length than more generalized lemurids. They noted that the only calcaneal form from which leaping behaviors can be inferred is that in which the degree of calcaneal elongation matches that of tarsiers and galagos. Miocene galagids, omomyiforms, and eosimiids were argued by Moyà-Solà et al. [7] to exhibit no evidence for leaping proclivity. Based on their analyses, they considered the evidence for leaping in the early euprimates lacking and concluded that the grasp-leaping hypothesis for euprimate origins could not be supported on these grounds. While the current study was in press, another study was published [38] describing what is possibly the most basal omomyiform species yet discovered, *Archicebus achilles*. The holotype for this new species, IVPP V18618, is a skull and skeleton more complete than any other available for an omomyiform. The combination of features described for this taxon and its basal position in primate phylogeny could be taken as providing additional support for Moyà-Solà et al.’s [7] hypothesis. This partial skeleton was argued to exhibit leaping features in the femur [38], but to have a calcaneus with a shorter distal segment than in *T. belgica*. This could suggest that calcaneal elongation and leaping demands are decoupled. However, the specimen also already has a strongly divergent hallux and tarsifulcrumating foot, so it is unclear what increases in calcaneal elongation in *T. belgica* would indicate about improved grasping.

In this study, we re-assess the allometric constraints on, and functional significance of, calcaneal elongation based on measurements from a new data set of 270 individual specimens representing 112 species of non-primate euarchontans, stem primates, all major prosimian genera except *Phaner* and *Allocebus*, and the majority of platyrrhine and catarrhine genera (Tables 1–2). Our primary analytical tool is regression. To account for phylogenetic autocorrelation, we use Phylogenetic Generalized Least Squares (PGLS) for regression and phylogenetic ANOVA. Finally, we reconstruct the evolution of calcaneal elongation using a Bayesian approach to ancestral state reconstruction (ASR).

**Table 1:** Extant taxon means and standard errors for body mass, distal segment lengths, elongation ratios, and residuals (see Table 2 for footnote explanations).

Taxon	Higher Taxon	N	Behavior^1^	est ln(BM)^2^	SE	ln(DL)	SE	ln(DL/TL)^3^	SE	Res A^4^	Res B^4^
*Euoticus elegantulus*	Galagonidae	1	AQ	5.849	–	2.65	–	−0.452	–	0.331	0.828
*Galago senegalensis*	Galagonidae	5	VCL/L	5.596	0.022	2.98	0.034	−0.324	0.785	0.442	1.221
*Galagoides demidoff*	Galagonidae	3	VCL/L	4.351	0.166	2.69	0.027	−0.266	0.806	0.415	1.242
*Otolemur crassicaudatus*	Galagonidae	4	VCL/L	7.322	0.026	3.10	0.013	−0.445	0.373	0.438	0.909
*Otolemur garnetti*	Galagonidae	3	VCL/L	6.886	0.018	3.03	0.035	−0.437	1.249	0.417	0.948
*Loris tardigradus*	Lorisidae	4	SC/T	5.269	0.075	1.35	0.023	−0.836	2.127	−0.092	−0.328
*Nycticebus coucang*	Lorisidae	2	SC/T	6.146	0.013	1.45	0.016	−1.016	1.255	−0.213	−0.447
*Nycticebus javanicus*	Lorisidae	1	SC/T	6.426	–	1.53	–	−0.995	–	−0.173	−0.437
*Arctocebus calabarensis*	Lorisidae	2	SC/T	5.341	0.011	1.15	0.003	−0.945	3.936	−0.196	−0.546
*Perodicticus potto*	Lorisidae	4	SC/T	6.813	0.08	1.60	0.061	−1.004	2.121	−0.155	−0.464
*Hapalemur griseus*	Lemuridae	4	VCL/L	6.67	0.1	2.11	0.037	−0.769	0.723	0.070	0.082
*Hapalemur simus*	Lemuridae	9	VCL/L	7.893	0.033	2.36	0.018	−0.85	1.008	0.072	0.027
*Avahi laniger*	Indriidae	1	VCL/L	7.193	–	2.05	–	−0.93	–	−0.055	−0.109
*Propithecus verreauxi*	Indriidae	4	VCL/L	7.752	0.035	2.29	0.008	−0.91	1.217	0.003	−0.008
*Indri indri*	Indriidae	3	VCL/L	8.655	0.038	2.68	0.037	−0.825	0.242	0.149	0.156
*Varecia variegata*	Lemuridae	3	AQ	8.254	0.083	2.43	0.015	−0.899	0.84	0.048	0.006
*Eulemur fulvus* ssp.	Lemuridae	6	VCL/L	7.511	0.045	2.31	0.015	−0.807	0.981	0.089	0.072
*Lemur catta*	Lemuridae	3	AQ	7.683	0.01	2.34	0.018	−0.832	1.393	0.076	0.059
*Lepilemur mustelinus*	Megaladapidae	5	VCL/L	6.593	0.05	2.20	0.026	−0.725	0.94	0.109	0.192
*Daubentonia madagascariensis*	Daubentoniidae	1	AQ	7.874	–	2.26	–	−0.893	–	0.028	−0.069
*Cheirogaleus major*	Cheirogaleiidae	1	AQ	5.791	–	1.92	–	−0.747	–	0.032	0.112
*Cheirogaleus medius*	Cheirogaleiidae	4	AQ	5.424	0.07	1.64	0.059	−0.711	0.962	0.043	−0.076
*Microcebus griseorufus*	Cheirogaleiidae	4	VCL/L	4.117	0.045	1.81	0.032	−0.477	1.213	0.188	0.421
*Mirza coquereli*	Cheirogaleiidae	2	VCL/L	5.641	0.03	2.09	0.001	−0.607	2.056	0.162	0.320
*Tarsius bancanus*	Tarsiidae	4	VCL/L	4.906	0.034	3.00	0.033	−0.256	0.768	0.463	1.413
*Tarsius tarsier*	Tarsiidae	3	VCL/L	5.094	0.034	2.97	0.013	−0.281	0.6	0.451	1.336
*Tarsius syrichta*	Tarsiidae	3	VCL/L	5.001	0.032	2.90	0.018	−0.289	0.261	0.437	1.290
*Alouatta caraya*	Atelidae	3	SC/T	8.707	0.175	2.38	0.036	−1.113	1.305	−0.135	−0.157
*Aotus azarae*	Cebidae	1	AQ	6.937	–	2.04	–	−0.905	–	−0.048	−0.055
*Aotus infulatus*	Cebidae	1	AQ	7.239	–	1.97	–	−0.872	–	0.006	−0.200
*Aotus nancymaae*	Cebidae	1	AQ	7.255	–	1.99	–	−0.924	–	−0.045	−0.184
*Ateles belzebuth*	Atelidae	1	SC/T/SUS	9.222	–	2.68	0.000	−0.877	–	0.136	0.014
*Ateles fusciceps*	Atelidae	1	SC/T/SUS	9.463	–	2.61	–	−0.947	–	0.082	−0.116
*Ateles geoffroyi*	Atelidae	1	SC/T/SUS	9.588	–	2.66	–	−0.906	–	0.131	−0.097
*Lagothrix lagotricha*	Atelidae	2	AQ	8.604	0.005	2.47	0.006	−0.943	1.789	0.028	−0.041
*Callicebus moloch*	Pithecidae	2	AQ	7.242	0.023	1.96	0.063	−0.871	1.588	0.007	−0.211
*Pithecia pithecia*	Pithecidae	2	VCL/L	8.053	0.234	2.10	0.007	−0.981	0.609	−0.048	−0.274
*Cacajao calvus*	Pithecidae	3	AQ	8.493	0.018	2.52	0.012	−0.858	2.917	0.105	0.036
*Chiropotes satanas*	Pithecidae	3	AQ	8.322	0.09	2.31	0.026	−0.929	0.79	0.022	−0.131
*Leontopithecus rosalia*	Callitrichidae	1	AQ	6.333	0	1.80	–	−0.887	0	−0.071	−0.144
*Callimico goeldii*	Callitrichidae	2	VCL/L	6.627	0.077	1.71	0.066	−0.892	4.461	−0.056	−0.307
*Callithrix jacchus*	Callitrichidae	1	AQ	5.703	–	1.51	–	−0.864	–	−0.091	−0.276
*Callithrix pygmaea*	Callitrichidae	2	VCL/L	4.733	0.137	1.10	0.013	−0.853	0.197	−0.146	−0.444
*Saguinus midas*	Callitrichidae	1	AQ	6.837	–	1.89	–	−0.847	–	0.003	−0.180
*Saguinus mystax*	Callitrichidae	2	AQ	5.897	0.008	1.59	0.018	−0.84	1.22	−0.054	−0.245
*Saimiri boliviensis*	Cebidae	2	AQ	6.899	0.051	1.95	0.021	−0.832	1.525	0.023	−0.135
*Saimiri sciureus*	Cebidae	1	AQ	6.79	–	1.95	–	−0.827	–	0.020	−0.108
*Cebus apella*	Cebidae	3	AQ	8.039	0.037	2.32	0.038	−0.848	3.108	0.084	−0.050
*Allenopithecus nigroviridis*	Cercopithecinae	1	AQ	8.307	–	2.41	–	−0.958	–	−0.008	−0.027
*Nasalis larvatus*	Colobinae	3	SC/T	9.734	0.057	2.75	0.026	−1.014	1.057	0.033	−0.044
*Erythrocebus patas*	Cercopithecinae	1	SC/T	8.581	–	2.48	–	−0.985	–	−0.016	−0.026
*Lophocebus albigena*	Cercopithecinae	1	AQ	8.984	0	2.55	–	−1.029	0	−0.033	−0.056
*Theropithecus gelada*	Cercopithecinae	2	SC/T	9.455	0.146	2.61	0.089	−1.123	2.503	−0.095	−0.114
*Trachypithecus cristata*	Colobinae	1	VCL/L	8.635	–	2.21	–	−0.965	–	0.008	−0.309
*Trachypithecus obscura*	Colobinae	1	VCL/L	8.645	–	2.44	–	−0.967	–	0.006	−0.082
*Papio ursinus*	Cercopithecinae	1	SC/T	10.066	–	2.74	–	−1.15	–	−0.080	−0.137
*Presbytis melalophos*	Colobinae	1	AQ	8.576	–	2.51	–	−0.912	–	0.057	0.006
*Procolobus badius*	Colobinae	2	VCL/L	8.853	0.169	2.55	0.144	−0.917	3.558	0.071	−0.024
*Pygathrix nemaeus*	Colobinae	1	VCL/L	9.436	–	2.74	–	−0.912	–	0.115	0.021
*Colobus guereza*	Colobinae	1	VCL/L	9.425	–	2.50	–	−1.127	−	−0.101	−0.217
*Chlorocebus cynosuros*	Cercopithecinae	1	AQ	8.648	–	2.49	–	−0.896	–	0.078	−0.032
*Chlorocebus aethiops*	Cercopithecinae	2	SC/T	8.322	0.037	2.33	0.056	−0.993	5.141	−0.042	−0.111
*Macaca fascicularis*	Cercopithecinae	3	SC/T	8.331	0.052	2.27	0.059	−0.987	5.802	−0.035	−0.173
*Macaca nigra*	Cercopithecinae	1	SC/T	8.677	–	2.49	–	−0.928	–	0.048	−0.039
*Pongo pygmaeus*	Hominidae	3	SC/T/SUS	10.944	0.143	2.74	0.027	−1.262	6.058	−0.132	−0.356
*Gorilla gorilla*	Hominidae	3	SC/T	11.618	0.151	2.87	0.109	−1.512	2.417	−0.336	−0.395
*Pan troglodytes troglodytes*	Hominidae	1	SC/T	10.954	−	2.87	–	−1.201	–	−0.071	−0.229
*Pan troglodytes verus*	Hominidae	2	SC/T	10.761	0.057	2.74	0.028	−1.278	7.971	−0.161	−0.311
*Hoolock hoolock*	Hylobatidae	1	SC/T/SUS	9.305	–	2.08	–	−0.961	–	0.057	−0.607
*Hylobates lar*	Hylobatidae	1	SC/T/SUS	9.017	–	2.13	–	−1.192	–	−0.193	−0.485
*Symphalangus syndactylus*	Hylobatidae	1	SC/T/SUS	8.536	–	2.16	–	−1.068	–	−0.102	−0.334
*Ptilocercus lowii*	Scandentia	3	NA	3.658	0.037	0.64	0.031	−0.974	1.103	−0.340	−0.635
*Tupaia* sp.	Scandentia	3	NA	4.883	0.046	1.18	0.205	−0.954	1.34	−0.236	−0.401
*Cynocephalus volans*	Dermoptera	2	NA	6.984	0.169	1.68	0.885	−0.95	0.333	−0.090	−0.426

**Table 2:** Fossil taxon means and standard errors for body mass, distal segment lengths, elongation ratios, and residuals.

Taxon	Higher Taxon	N	Behavior^1^	est ln(BM)^2^	SE	ln(DL)	SE	ln(DL/TL)^3^	SE	Res A^4^	Res B^4^
*Cantius mckennai*	Notharctinae	3	NA	6.472	0.129	2.10	0.039	−0.849	1.155	−0.023	0.122
*Cantius abditus*	Notharctinae	6	NA	7.05	0.1	2.25	0.016	−0.889	0.945	−0.024	0.127
*Cantius feretutus*	Notharctinae	2	NA	6.658	0.056	2.08	0.022	−0.888	1.214	−0.050	0.055
*Cantius trigonodus*	Notharctinae	4	NA	6.714	0.162	2.21	0.022	−0.846	1.962	−0.004	0.171
*Cantius ralstoni*	Notharctinae	1	NA	6.12	–	1.98	0.004	−0.789	–	0.013	0.090
*Notharctus* sp.	Notharctinae	9	NA	7.743	0.093	2.29	0.016	−0.894	1.106	0.018	−0.006
*Smilodectes gracilis*	Notharctinae	2	NA	7.824	0.071	2.30	0.018	−0.918	1.357	0.000	−0.016
*Anchomomys frontanyensis*	Cercamoniinae	2	NA	4.74	0.046	1.68	0.030	−0.662	1.825	0.046	0.135
*Adapis parisiensis*	Adapinae	6	NA	6.972	0.035	1.61	0.045	−1.241	1.483	−0.381	−0.493
*Adapis* sp.	Adapinae	1	NA	7.71	–	1.98	–	−1.119	–	−0.209	−0.308
*Leptadapis magnus*	Adapinae	11	NA	8.768	0.065	2.32	0.026	−1.186	2.197	−0.204	−0.232
*Asiadapis cambayensis*	Asiadapinae	1	NA	5.83	–	1.45	–	−0.939	–	−0.157	−0.368
*Marcgodinotius indicus*	Asiadapinae	5	NA	4.786	0.082	1.13	0.028	−0.869	1.506	−0.158	−0.427
*Teilhardina belgica*	Omomyiformes	4	NA	3.818	0.029	1.28	0.034	−0.643	0.941	0.002	−0.035
*Absarokius* sp.	Omomyiformes	1	NA	4.52	–	1.73	–	−0.611	–	0.082	0.240
*Tetonius homunculus*	Omomyiformes	1	NA	4.403	–	1.58	–	−0.685	–	0.000	0.119
*Arapahovius* sp.	Omomyiformes	1	NA	4.095	–	1.60	–	−0.524	–	0.140	0.216
*Washakius insignis*	Omomyiformes	1	NA	4.862	–	1.72	–	−0.626	–	0.090	0.144
*Shoshonius cooperi*	Omomyiformes	1	NA	4.62	–	1.70	–	−0.619	–	0.081	0.185
*Omomys carteri*	Omomyiformes	5	NA	5.833	0.051	2.05	0.028	−0.641	0.843	0.141	0.232
*Ourayia uintensis*	Omomyiformes	1	NA	7.065	–	2.38	–	−0.722	–	0.144	0.254
*Hemiacodon gracilis*	Omomyiformes	1	NA	6.079	–	2.03	–	−0.671	–	0.128	0.150
*Necrolemur antiquus*	Omomyiformes	1	NA	5.559	–	2.57	–	−0.404	–	0.360	0.820
*Komba robustus*	Lorisiformes	2	NA	5.691	0.004	1.97	–	−0.605	4.829	0.167	0.187
*Eosimias sinensis*	Eosimiidae	3	NA	4.45	0.249	1.30	0.087	−0.741	2.409	−0.053	−0.173
Parapithecidae var.	Parapithecidae	5	NA	6.526	0.202	2.06	0.057	−0.961	2.524	−0.132	0.068
*Proteopithecus sylviae*	?Parapithecidae	1	NA	5.641	–	1.70	–	−0.825	–	−0.056	−0.071
*Mesopropithecus dolichobrachion*	Indrioidea	1	SC/T/SUS	8.046	–	2.49	0.009	−1.182	–	−0.249	0.118
*Paleopropithecus sp.*	Indrioidea	3	SUS	9.254	0.095	2.04	–	−1.510	7.428	−0.495	−0.634
*Babakotia radioflai*	Indrioidea	3	SUS	8.622	0.132	1.79	0.099	−0.854	1.459	0.118	−0.726
*Archaeolemur.sp.*	Indrioidea	5	SC/T	9.628	0.011	2.20	0.000	−1.120	1.188	−0.080	−0.567
*Pachylemur insignis*	Lemuridae	1	SC/T	9.043	–	2.50	0.017	−1.234	–	−0.234	−0.121
*Megaladapis.sp.*	Megaladapidae	3	VC	10.473	0.180	2.88	0.051	−1.067	3.987	0.031	−0.099
*Plesiadapis cookei*	Plesiadapidae	1	NA	7.683	–	1.72	–	−1.156	–	−0.248	−0.561
*Nannodectes gidleyi*	Plesiadapidae	1	NA	5.9	–	1.12	–	−1.165	–	−0.378	−0.715
*Carpolestes simpsoni*	Carpolestidae	1	NA	5.149	–	0.97	–	−0.926	–	−0.190	−0.678
*Dryomomys szalayi*	Micromomyidae	1	NA	2.359	–	−0.19	–	−1.146	–	−0.600	−1.140
*Phenacolemur simonsi*	Paromomyidae	1	NA	4.96	–	0.88	–	−1.103	–	−0.380	−0.720

1 Behavior codes based on the literature (see methods): Abbreviations: VCL, vertical clinging & leaping; L, specialized/frequent leaper and/or grasp-leaper; AQ, arboreal quadruped with unspecialized/infrequent leaping; SC, slow climber (virtually no leaping); T, terrestrial; SUS, suspensory; NA, not applicable (extinct taxon). SC/T/SUS, taxon is characterized by one or more of the three indicated categories.

2 Natural log of body mass (BM) estimated from cuboid facet size using the following equation: ln[BM] = 1.3274*ln[CW*CD]+3.0238 (see Table S1 in File S1 for data).

3 Natural log of the ratio of calcaneal distal length (DL) to calcaneal total length (TL).

4 Res, Residual Elongation from lines calculated in *caper* using “All Euprimates” with n = 100 species (average from trees 1–4) (Fig. 8)]. Res A: ln[DL/TL] = −0.068(SE ±0.011)*ln[BM]+ −0.39(SE ±0.08); Res B (based on regression of absolute distal calcaneal length versus body mass): ln[DL] = 0.25(SE ±0.022)*ln[BM]+0.36(SE ±0.17).

Other abbreviations: est, estimate; ln, natural logarithm; n, sample size; SE = standard error.

We focus our re-assessment on the following questions: 1) Does variation in body mass explain variation in relative calcaneal elongation across primates? 2) Does variation in locomotor behavior explain variation in relative calcaneal elongation across primates? 3) Is locomotion predictable from calcaneal elongation, and if so, in what contexts? 4) What do ancestral state reconstructions of calcaneal elongation and body mass reveal about the role of leaping in the origin and early evolution of primates? In the course of addressing these questions we further test two specific conclusions of Moyà-Solà et al. [7]. Namely, that 1) calcaneal elongation residuals do not coincide with degree of leaping in euprimates and 2) calcaneal elongation can be explained by the acquisition of a grasping foot. For the first issue, our use of phylogenetic comparative methods allows for an evaluation of the possibility that phylogenetic covariance and clade shifts (as defined by Nunn [24]) in calcaneal elongation might have obscured behavioral associations with leaping when looking at primates as a whole, an issue not addressed by Moyà-Solà et al. [7]. The second issue is also tested through novel application of phylogenetic comparative methods, with an emphasis on comparing basal euprimates to their closest relatives. The fossil record of stem primates provides a direct test for assessing whether changes in calcaneal elongation correspond to the acquisition of a grasping foot [19]. If a grasping foot explains increases in calcaneal elongation, then increases in hallucal specialization in stem primates should be accompanied by acquisition of euprimate-like distal calcaneal segment length. Finally, we evaluate the significance of new morphology presented by the basal omomyiform, *Archicebus achilles* [38] in the context of our analyses.

Instead of comparing absolute calcaneal measures to species mean mass from the literature [7], we take an approach that is biomechanically more pertinent, easier to interpret, and provides greater sample sizes for analysis. We plot ratios of calcaneal distal segment length (DL) (i.e., distal to the crurotarsal, or “upper ankle” joint) to total calcaneal length (TL) on body mass estimates (BM) generated from the length and width of the calcaneal cuboid facet of the same calcaneal specimen (see **Materials and Methods**). The defined ratio is equivalent to a load arm-lever arm ratio. This metric therefore summarizes the functionally relevant components to leaping. When this ratio is relatively high, accelerations for a given rate of contraction by the plantar flexors will be high (as needed by small-bodied leapers). When it is low, mechanical advantage will be high (as needed by large-bodied leapers) and acceleration will be lower [31]. Our analyses of new fossils of early euprimates, together with a comprehensive sample of extant primates, non-primate euarchontans, and fossil-stem primates, provide a view of the fundamental allometry of this system and allow for a more definitive, cohesive interpretation of the functional significance of variation in calcaneal elongation in early euprimates. In addition, these analyses will lead to a reconstruction of the variation that occurred during the transition up to, and through, the early evolution of euprimates.

In the sections that follow, we begin by demonstrating a strong correlation between body mass and measurements representing the area of the calcaneocuboid facet in extant primates; the area of this facet also accurately predicts mass of extant non-primate euarchontans indicating its general applicability to stem primates as well. Following this, the remainder of our investigation into calcaneal allometry builds out from patterns observed among five species of the long-ranging (∼2 million years) fossil genus *Cantius*, which is thought to represent no more than two anagenetic lineages of notharctine primates [39]. A previous study of *Cantius* calcanei [40] established relative stasis in calcaneal shape combined with significant gradual increases in absolute size through time. The strength of using these fossil lineages as a starting point in our evaluation of the effects of body size variation on calcaneal morphology is that it 1) allows investigation of morphology across a body size range beyond what can be observed within an extant species, 2) eliminates the confounding factor of other morphological differences that might represent selection for significantly different locomotor styles (typical when comparing different species not representing different points along an evolving lineage), and 3) allows an analysis of taxa separated evolutionarily by much less time than most extant sister taxa, which appear to have diverged at least several million years ago in most cases [41].

## Materials and Methods

### Sample

We measured the calcaneus of 73 extant species (individual n = 168) and 38 fossil species (individual n = 102) euarchontan species. Included is UF 252980, a newly discovered specimen that is determined to be among the oldest and most complete known for *C. ralstoni* (see Results). To our knowledge, for the measurements of interest (CW, DC, DL, and TL: see below), our sample is the largest ever analyzed for the euprimates *Cantiu*s (five species; n = 16) and *Notharctus* and *Smilodectes* (two species; n = 11). Other fossil euprimate taxa include omomyiforms (ten species; n = 17), asiadapines (two species; n = 6), adapines (three species; n = 18), *Anchomomys frontanyensis* (n = 2), *Komba robustus* (n = 2), parapithecids (two species; n = 6), and eosimiids and/or “protoanthropoids” (n = 5). Our extant sample includes lemuriforms (14 species; n = 50), lorisiforms (ten species; n = 29), *Tarsius* (three species; n = 10), platyrrhines (21 species; n = 36), cercopithecoids (16 species; n = 23), and hominoids (7 species; n = 12). A sample of extant non-primate euarchontans includes *Ptilocercus lowii* (n = 3), *Tupaia* sp. (n = 3), and *Cynocephalus volans* (n = 2). The sample of fossil non-euprimate euarchontans includes plesiadapids (two species; n = 2), *Dryomomys szalayi* (n = 1), *Phenacolemur simonsi* (n = 1), and *Carpolestes simpsoni* (n = 1). See Tables 1–2 for summary of sample. See Table S1 in File S1 for all data.

### Fossil Specimens

As per the standards of *PLoS ONE*, we provide a list of fossil specimens used in this study and indicate individuals and/or institutions that granted permission for their study parenthetically in this list. As well, where relevant, we indicate permit numbers. Furthermore, we affirm that all necessary permits were obtained for the described study, which complied with all relevant regulations. Institutional abbreviations are found in the next section. All fossil specimens and extant specimens can be found listed with measurements and other details in Table S1 in File S1. Specimens used in this study include the following: UM 79150 *Cantius ralstoni*, UM “SLC VC Msc6” *Cantius ralstoni*, UM 98604 *Omomys carteri*, UM 87990 *Plesiadapis cookei*, UM 101963 *Carpolestes simpsoni*, UM 41870 *Dryomomys szalayi* (permission to study from P. Gingerich); UF 252980 *Cantius ralstoni* [collected under Bureau of Land Management permits to JIB (PA04-WY-113, PA10-WY-185)]; USGS 5897 *Cantius mckennai,* USGS 25029A *Cantius mckennai,* USGS 25029B *Cantius mckennai,* USGS 6769 *Cantius trigonodus,* USGS 6765 *Cantius trigonodus,* USGS 21829 *Cantius trigonodus,* USGS 21767 *Cantius trigonodus,* USGS 21765 *Cantius trigonodus,* USGS 21776 *Cantius frugivorus,* USGS 21828 *Cantius frugivorus,* USGS 6792 *Cantius frugivorus,* USGS 21774 *Cantius abditus,* USGS 21775 *Cantius abditus,* USGS 21827 *Cantius abditus,* USGS 21825 *Cantius abditus,* USGS 21771 *Cantius abditus,* USGS 6783 *Cantius abditus* (permission to study from K. Rose and USNM); AMNH 16852 *Cantius trigonodus,* AMNH 1727 *Notharctus* sp., AMNH 131956 *Notharctus* sp., AMNH 131955 *Notharctus* sp., AMNH 55061 *Notharctus* sp., AMNH 11474 *Notharctus* sp., AMNH 13766 *Notharctus* sp., AMNH 131945 *Notharctus* sp., AMNH 11478 *Notharctus* sp., AMNH 129382 *Notharctus* sp., AMNH 131774 *Smilodectes* sp., AMNH 131763 *Smilodectes* sp., AMNH 10016 *Adapis parisiensis,* AMNH 88820 *Tetonius* cf. *homunculus,* AMNH 88821 *Tetonius* cf. *homunculus,* AMNH 29164 cf. *Omomys,* AMNH 88824 *Washakius insignis,* AMNH 12613 *Hemiacodon gracilis,* AMNH 17379 *Nannodectes gidleyi* (permission to study from J. Meng); GU 709 *Marcgodinotius indicus,* GU 751 *Marcgodinotius indicus,* GU 1644 *Marcgodinotius indicus,* GU 1643 *Marcgodinotius indicus,* GU 710 *Marcgodinotius indicus,* GU 760 *Asiadapis cambayensis* (permission to study from K. Rose); NMB QE 644 *Adapis parisiensis,* NMB QE 741 *Adapis parisiensis,* NMB QE 779 *Adapis parisiensis,* NMB QF 558 *Adapis parisiensis,* NMB QH 640 *Adapis parisiensis,* NMB QE 530 *Adapis* sp., NMB QW 1676 *Leptadapis magnus,* NMB QE 604 *Leptadapis magnus,* NMB QE 830 *Leptadapis magnus,* NMB QE 920 *Leptadapis magnus,* NMB QF 421 *Leptadapis magnus* (permission to study from L. Costeur); ACQ 265 *Leptadapis magnus,* ACQ 266 *Leptadapis magnus,* ACQ 267 *Leptadapis magnus,* ACQ 268 *Leptadapis magnus,* PQ 1746 *Leptadapis magnus,* PQ 1747 *Leptadapis magnus* (permission to study from M. Godinot); IPS 7748 *Anchomomys frontanyensis*, IPS 7769 *Anchomomys frontanyensis* (measurements taken from [24]); IRSNB M 1247 *Teilhardina belgica*, IRSNB M 1236 *Teilhardina belgica*, IRSNB M 1237 *Teilhardina belgica*, IRSNB 16786-03 *Teilhardina belgica* (permission to study from T. Smith); UCM 67850 *Arapahovius gazini,* UCM 67907 *Absarokius* sp., UCM 67679 *Omomys carteri,* UCM 68745 *Omomys carteri,* UCM 69065 *Omomys carteri,* UCM 67678 *Omomys carteri* (permission to study from H. Covert); CM 69765 *Shoshonius cooperi*, IVPP 12313 *Eosimias,* IVPP 12280 *Eosimias,* IVPP 11851 eosimiid, IVPP 11847 eosimiid, IVPP 11848 eosimiid (permission to study from K.C. Beard); SDNH 4020-60933 *Ourayia uintensis* (permission to study from R. Dunn); PMZ A/Z 637 *Necrolemur zitteli* (permission to study from A. Rosenberger via P. Schmid); KNM-SO 1364 *Komba robustus* (measurements from cast held by E. Seiffert); DPC 10926a *Prolemur simus,* DPC 10926b *Prolemur simus,* DPC 10926c *Prolemur simus,* DPC 10975a *Prolemur simus,* DPC 6818 *Prolemur simus,* DPC 6652a *Prolemur simus,* DPC 6652c *Prolemur simus,* DPC 10988a *Prolemur simus,* DPC 9925 *Prolemur simus,* DPC 11843-B cf. *Varecia variegata,* DPC 10975b cf. *Indri indri,* DPC 24776 *Proteopithecus sylviae,* DPC 8810 Parapithecidae (?*Apidium*), DPC 15679 Parapithecidae, DPC 2381 Parapithecidae, DPC 20576 Parapithecidae, DPC 17214A(L&R) *Paleopropithecus* cf. *ingens*, DPC 17164 *Paleopropithecus* sp., DPC 11824 (L&R) *Babakotia radofilai,* DPC 11818 *Babakotia radofilai,* DPC 6833 *Mesopropithecus dolichobrachion,* DPC 11822 *Pachylemur insignis,* DPC 9106 (L&R) *Archaeolemur* cf. *edwardsi,* DPC 12879 (L&R) *Archaeolemur* sp., DPC 18740 *Archaeolemur majori,* DPC 18936 *Megaladapis* cf. *madagascariensis,* DPC 13733 *Megaladapis madagascariensis,* DPC 9089 *Megaladapis* cf. *madagascariensis/grandidieri* (permission to study from G. Gunnell); USNM 442240 Paromomyidae sp. indet.

### Institutional Abbreviations

AMNH, American Museum of Natural History, New York, NY, USA; CGM, Egyptian Geological Museum, Cairo, Egypt; DPC, Duke Lemur Center Division of Fossil Primates, Durham, NC, USA; CM, Carnegie Museum of Natural History, Pittsburgh, PA, USA; GU, H.N.B Garhwal University, Srinagar, Uttarakhand, India; IPS, Institut de Paleontologia de Sabadell ( = Institut Català de Paleontologia Miquel Crusafont), Spain; IRSNB, Institut Royal des Sciences Naturelles del Belgique, Brussels, Belgium; IVPP, Institute of Vertebrate Paleontology and Paleoanthropology, Chines Academy of Sciences, Beijing, China; KNM, Kenya National Museum, Nairobi, Kenya; MCZ, Museum of Comparative Zoology, Harvard University, Cambridge, MA, USA; MNHN, Muséum National d’Histoire Naturelle, Paris, France; NMB, Naturhistoisches Museum Basel, Basel, Switzerland; NMNH, Smithsonian Institution National Museum of Natural History, Washington, D.C., USA; NYCEP, New York Consortium in Evolutionary Primatology, New York, NY, USA; PMZ, Paleontology Museum of the University of Zurich, Zurich, Switzerland; SBU, Stony Brook University, Stony Brook, NY, USA; SDNHM, San Diego Natural History Museum, San Diego, CA, USA; UCM, University of Colorado Museum of Natural History, Boulder, CO, USA; UF, University of Florida, Florida Museum of Natural History, Gainesville, FL, USA; UM, University of Michigan, Ann Arbor, MI, USA; USGS, U.S. Geological Survey, Denver, CO, USA; UNSM, University of Nebraska Science Museum, Lincoln, NB, USA; USNM, United States National Museum, Smithsonian Institute, Washington DC, USA; RS, Randall Susman personal collection.

### Analysis

#### Generation of digital sample

All measurements were taken on 3D digital surface models. These were created by various scanning modalities. Most specimens were scanned using one of five instruments: at SBU, two different ScancoMedical brand machines were used (VivaCT 75, µCT40); at the AMNH Microscopy and Imaging Facility, a Phoenix brand v/tome/x s240 was used; and for specimens of *Nasalis*, *Gorilla*, *Pan*, and *Pongo* a GE eXplore Locus SP machine was used at the Ohio University µCT Facility. Some gorillas and a couple other large species were scanned with GE Medical CT scanner. A few specimen scans were generated with Breuckmann Structured light scanner provided to the New York Consortium in Evolutionary Primatology by funding of the National Science Foundation. Finally, several specimens were not scanned but measured manually with calipers (the measurements are quite basic: Fig. 1B) Specimens were mounted in foam or packed in cotton to prevent movement while scanning. Most specimens were scanned at a resolution of 39 microns or less. The highest resolutions used were on the order of 3–5 microns for the very smallest fossil specimens. The scanning resolution was usually high enough to result in an initial surface with 1–5 million faces, but all specimens were down-sampled to between 300,000 and 500,000 faces after fitting an initial surface to the data. See appendix for the original scan resolution of each specimen based on a microCT data set.

#### Measurements and Standard Regressions

In most regards, our approach is traditional: we measure total length (TL) and distal segment length (DL) of the calcaneus [28], [33], [40] (Fig. 1B). Moyà-Solà et al. [7] used a slightly different approach, and split the anterior (distal) and posterior (proximal) halves of the calcaneus at the midpoint of the ectal facet. Unless selection acts differently on either side of the proximodistal midpoint of ectal facet (i.e., to stretch or compress each half in order to effect different degrees of calcaneal distal elongation), the patterns generated by their method, versus more standard methods should be equivalent. In addition, illustrations in Hall-Craggs [42] show the center of rotation of the astragalotibial joint to be anteriorly adjacent to the ectal facet. Regardless, we think the measurement landmark on the ectal facet used is of minor concern relative to the concern that the particular landmark of choice can be determined repeatably: i.e., the distal boundary of the ectal facet used here is very easy to repeatably locate in almost all included taxa. Instead of then using log-transformed raw data in our analyses, as in Moyà-Solà et al. [7], we calculated elongation ratios as described in the introduction: Calcaneal Elongation = ln(DL/TL).

Regardless of the variable of choice (raw measures or ratios), assessing allometric trends in fossil primates has been hindered previously by lack of body mass (BM) information on isolated calcanei (Moyà-Solà et al. [7] use species mean body mass and mean calcaneal segment lengths). We have generated a regression to estimate body mass based on cuboid facet area as represented by the product of the maximum mediolateral width (cuboid facet width: CW) and maximum dorsoplantar depth (cuboid facet depth: CD) of this facet from a taxonomically comprehensive sample of primates (Fig. 2B), which allows estimation of body mass for any calcaneus with a largely intact cuboid facet. The sample for this regression includes 129 individuals from all major clades (see Table S1 in File S1). Body mass data were obtained mainly from Smith and Jungers [43] with some data coming from *Primates in Perspective* [44] and *Walker’s Mammals of the World* [45]. Given a strong correlation between body mass and the calcaneal cuboid facet area one might question the wisdom of using mass estimates derived from measured variables instead of simply using the measured variables themselves. To explain our reasoning some reporting of results is necessary up front.

The equation derived from the linear relationship between logged body mass and logged cuboid facet area is the following: ln(BM) = 1.3274*ln(CW*CD)+3.0238, r^2^ = 0.98. The obtained slope of 1.3274 of the regression and its confidence interval (95% CI: 1.29–1.36) excludes the value 1.5, which is the expectation for an isometric relationship between area and volume. Therefore, regressions of cuboid facet area (CW*CD) and calcaneal elongation directly would not be accurate representations of mass-related scaling of calcaneal elongation despite an excellent correlation between body mass and the area of the calcaneal cuboid facet. We therefore are obliged to use body mass estimates, rather than CW*CD as a covariate for calcaneal elongation. These estimates were used without Quasi-Maximum Likelihood Estimate (QMLE) correction [46]. We decided not to use the QMLE after comparing slopes and intercepts that resulted using both corrected and uncorrected data. Slopes were always either identical or the difference between slope estimates was never more than 0.5% (i.e., one half of one percent) of the standard error of the slope estimates. The difference between intercept estimates, likewise, was never more 50% of the standard error of the estimates (i.e., well within 95% confidence limits). Thus we found no reason to increase the number of estimated parameters by adding a QMLE correction. In Table S1 in File S1, we report anti-logged transformations of body mass estimates used in these analyses for individual specimens; in this case a QMLE correction is applied to give the reader an accurate sense of the estimates.

One might also worry about the phylogenetic specificity of this relationship. However, subdividing the sample into “prosimian” and anthropoid groups reveals no significant changes in slope or intercept (“prosimians” [n = 73, slope 95% C.I. = 1.299–1.395; intercept 95% C.I. = 2.875–3.159], anthropoids [n = 56, slope 95% C.I. = 1.320–1.435; intercept 95% C.I. = 2.541–3.015]). Thus, the taxonomically combined regression can be safely applied to any primate without having major concern about how its phylogenetic relationships might bias body mass estimates of the regression.

Regressions of calcaneal elongation on estimated body mass were also run as ordinary least squares in the program PAST. It could be argued that reduced major axis is a more appropriate method for estimating relationships between variables analyzed here [47], since we are, at this point, modeling a relationship between two variables, instead of predicting one from the other. Nevertheless, we have two important reasons for using least squares in this study: 1) when assessing allometry using a ratio of two linear measurements (as we have done) the null hypothesis (isometry) is a slope of zero and/or no significant relationship between body mass and the ratio of interest. Therefore, when using ratios against body mass, least squares is the most conservative approach for testing for departures from isometry as it tends to underestimate the slope of a line when the correlation is low. OLS cannot necessarily be considered a conservative approach when using absolute measures against body mass: to demonstrate allometry between mass and another measurement it must be shown that the slope of the relationship is significantly different from what is predicted for isometric scaling. A slope of zero, or the lack of a significant relationship, actually implies allometry when regressing an absolute measure on body mass. Furthermore, for a positively allometric relationship, a poor correlation could cause the slope to drop enough to mimic isometry or negative allometry. This situation cannot result when using least squares to regress body mass against a ratio – poor correlations bring the slope towards zero (suggesting isometry) whether the true relationship is positive or negative allometry. Secondly, and more pragmatically relative to the design of our study, there is no well-tested code for phylogenetic RMA currently available.

#### Phylogenetic Methods

Phylogenetic statistical methods use the pattern of connectivity and branch lengths in a phylogenetic tree to assess the presence of phylogenetic correlation of values in test variables, and to adjust standard error estimates on statistical tests to account for violation of the typical assumption in parametric tests that data points are metrically independent of each other.

It is intuitive and well-demonstrated [24] that different patterns of connectivity and the branch lengths of the phylogenetic distance matrix can result in different “phylogenetically adjusted” patterns. Nevertheless, it is generally acknowledged from simulation studies [24] that even when there are errors in the phylogeny, the results are more accurate than when assuming a “star phylogeny” ( = no phylogeny). In this study we utilize the “Phylogenetic Generalized Least Squares” (PGLS) [48] approach to incorporate phylogenetic information. We use this for three different specific analyses: 1) evaluation of trait correlation, 2) ANOVA on calcaneal elongation and distal segment length residuals from a phylogenetically-adjusted regression line, and 3) ancestral state reconstruction (ASR). The first two analyses were run using the *caper* [72] package implemented in R 2.15.0. The third was run using the *Continuous* module in *BayesTraits* 1.1B [70]. For all phylogeny-adjusted analyses, species mean values were used to represent each OTU of the sample.

The PGLS regressions and Bayesian reconstructions of continuous ancestral states presented here are each based on one of six different time-scaled phylogenetic trees of living and extinct primates, the overall topologies of which were computed by combining various source trees ([i.e., trees from previously published analyses, combined with new analyses performed specifically for this study [see below]) using the Matrix Representation with Parsimony (MRP) or “supertree” approach. MRP was used to combine the extant primate phylogeny of Springer et al. ([49], based on 61,199 base pairs from 69 nuclear genes and 10 mitochondrial genes) with the molecular analysis of Janečka et al. [50], the morphology-based trees of Tornow ([51], for omomyiforms; his Figure 10), Rose et al. ([52], for basal omomyiforms, their figure 13C), and Bloch et al. ([19], for plesiadapiforms; their Figure 4). These trees were combined with strict consensus topologies derived from *de novo* parsimony analyses of a matrix that has been used in several recent studies [52]–[57]; modified most recently by Gladman et al. [58] and Boyer and Seiffert (in press) that includes plesiadapiforms and several Paleogene primates (omomyiforms, adapiforms, and early anthropoids). This matrix was analyzed in PAUP 4.0b10 under various constraints so that the evolution of distal calcaneal elongation could be evaluated across several competing phylogenetic hypotheses: 1) with a molecular scaffold enforced, based on the results of Springer et al. [49], but with all extinct taxa unconstrained, 2) with the same molecular scaffold enforced, and adapiforms constrained to be more closely related to tarsiers and/or anthropoids than strepsirrhines [59], and 3) with the same molecular scaffold enforced, with tarsiers constrained to be more closely related to anthropoids than to any omomyiform [35], [52], [60], [61]. All parsimony analyses were performed (in PAUP 4.0b10 [62]) with random addition sequence and TBR branch-swapping across 10,000 heuristic search replicates. Some multistate characters were treated as ordered and were scaled so that transitions between “fixed” states in an ordered morphocline were equal to one step (polymorphisms were assigned their own state, intermediate between “fixed” states in each morphocline). MRP matrices were created and concatenated in Mesquite 2.75, and parsimony analyses were also run in PAUP 4.0b10.

**Figure 4:**
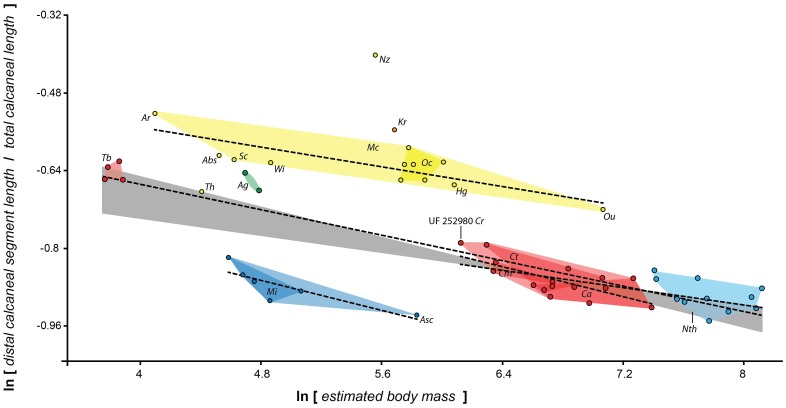
Plotting early fossil forms reveals allometric scaling within and between certain clades. Different interecepts but similar slopes of scaling of distal calcaneal elongation index to body mass (as reconstructed from cuboid facet area) characterize different groups of early primates. There is a low- (based on Asiadapinae), intermediate- (based on all or subsets of the following taxa: *Cantius*, *Notharctus*, *Smilodectes* and *Teilhardina*) and high-elongation line (based on Omomyinae: see Table 2); see also Fig. 3B. The intermediate elongation line appears to be primitive, as the non-primate taxa plotting near the low line (some scandentians and plesiadapiforms) actually exhibit a non-significant relationship between mass and elongation. Dashed lines represent ordinary least squares lines for different groups. Adapiforms are represented by a line describing *Cantius* species only and one representing all notharctids. The gray area represents the space in between the mean for the two lines. Polygons: Red, *Cantius* and *Teilhardina*; Light blue, *Notharctus*; Dark blue, asiadapines; Yellow, Omomyines; Solid yellow, *Omomys*; Green, *Anchomomys*. Th, *Tetonius homunculus*. See Figure 3 caption for taxon abbreviations.

**Figure 10:**
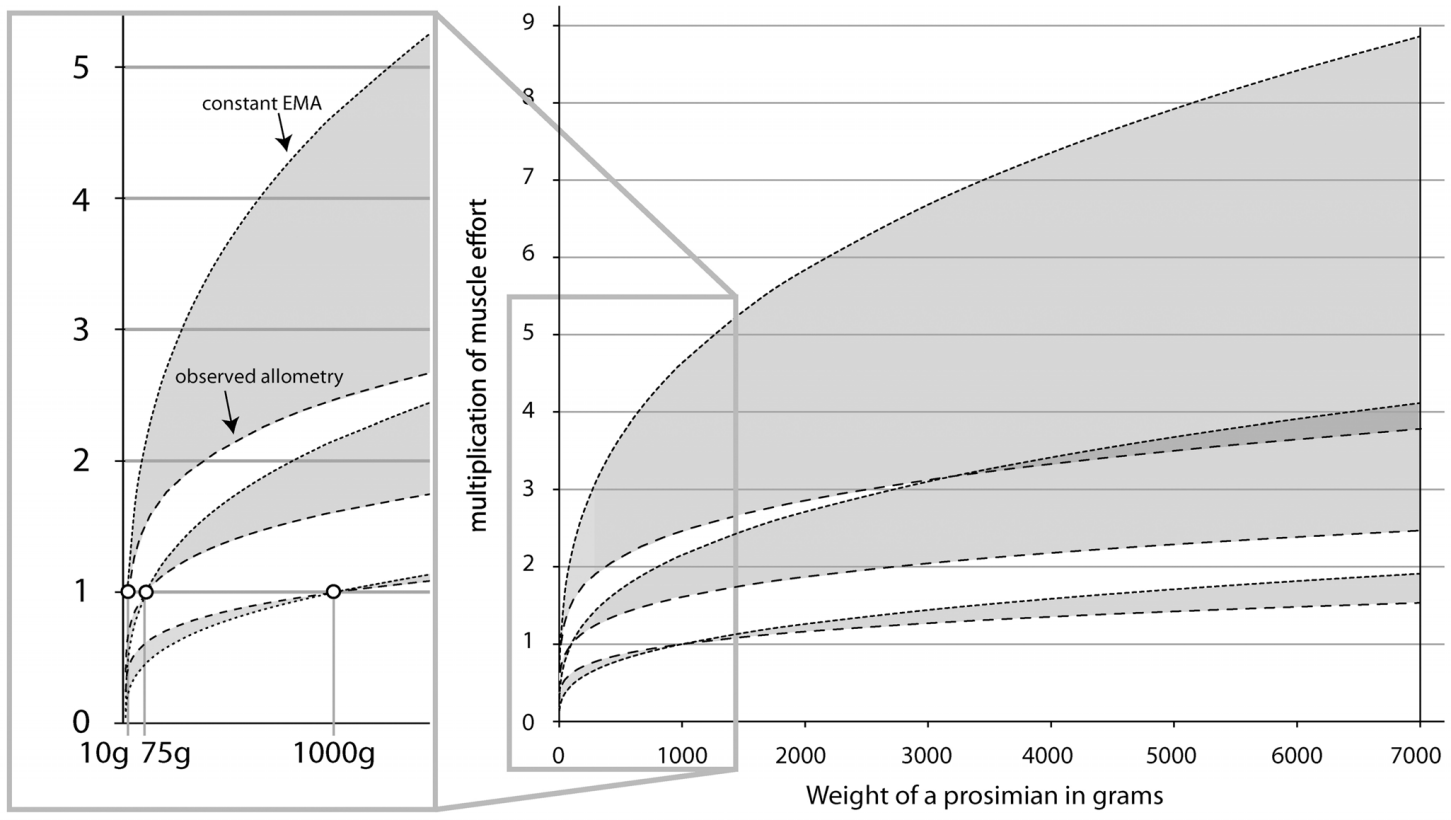
Modeling force magnification plot. We modeled the biomechanical significance of the empirically demonstrated allometry by assessing the scaling of the relative force needed to balance the load and lever arms of the calcaneus for a primate of varying body mass. We modeled this with three different “ancestral sizes” 10 g, 75 g and 1,000 g. For each starting weight we modeled the increase in relative effort required by the *m. triceps surae* muscles attaching to the calcaneal tuber for size increase with a constant load arm/lever arm ratio (upper, thin-dashed lines) and with the expected allometric change in load arm/lever arm ratios (lower, thick-dashed lines). We plot values up to 7 kg, the weight of the largest extant prosimians, and show that the observed allometry reduces the effort multiplication required by the animals’ hindlimbs by as much as a 9-to−4 ratio. Note also that evolving to smaller body sizes yields a diminished effort for constant and allometrically changing load arm/lever arm ratios. This opens the possibility for evolving “off” the line when body size decreases, without incurring extra effort on the muscular system (see text).

**Figure 5:**
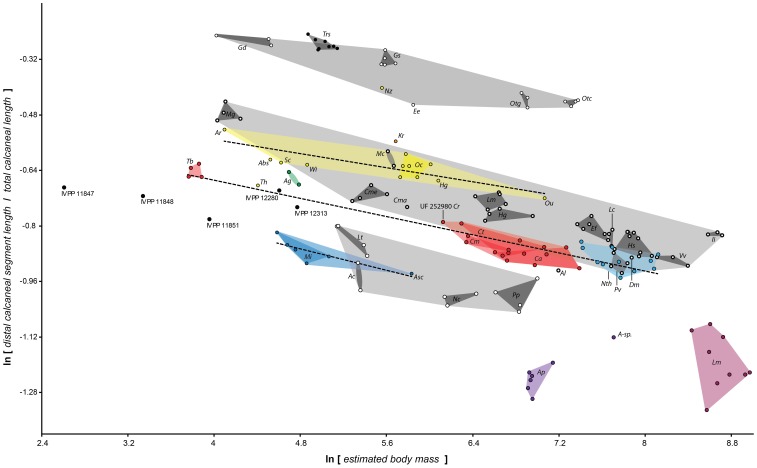
Plot of fossils with extant forms imposed shows similar allometric scaling relationships characterize in living taxa. To better understand the phenetic associations of the fossils and to help consider the functional implications of their proportions, we plot them with extant taxa. Each data point represents an individual. Dark gray polygons represent species groups. Light gray polygons bound different extant prosimian radiations: Upper polygon, Galagidae; middle polygon, lemuriformes; lower polygon, Lorisidae. (see Figures 2 and 3 for taxon abbreviations). “IVPP” specimens are eosimiids from Shanguang fissure fills with taxon identifications given in Gebo et al. (2000).

Subfossil lemurs posed special problems for the MRP approach because not all have been included in *bona fide* phylogenetic analyses, leaving us with no option but to graft them onto the MRP supertrees in their most probable phylogenetic positions, given recent assessments of their morphology. The subfossil lemurid *Pachylemur* appears to be the sister taxon of the lemurid *Varecia* based on genetics [63] and morphology [64], [65]; as such we placed this genus mid-way along the *Varecia* branch. The only palaeopropithecid that has been included in a molecular phylogenetic analysis is *Palaeopropithecus* [66], and that study supported its proposed placement as an indrioid. We used the palaeopropithecid topology proposed by Jungers et al. ([67], i.e. (*Mesopropithecus*, (*Babakotia*, *Palaeopropithecus*))), and placed this clade as the sister group of extant indriids. Internodes within Palaeopropithecidae were spaced evenly, as there are currently no objective means for estimating divergence times within the family. Finally, the archaeolemurid *Archaeolemur* was placed as the sister group of palaeopropithecids and indriids, again following Jungers et al.’s [67] and Orlando et al.’s [66] placement of archaeolemurids with indrioids (note, however, that Orlando et al. did not resolve the relationships of archaeolemurids, palaeopropithecids, and indriids). *Megaladapis* was placed as the sister taxon of Lemuridae, following Orlando et al. [66], and was grafted onto the lemurid stem lineage at its mid-point. In other parts of the tree, some additional assumptions had to be made due to a lack of taxonomic overlap in the source trees: 1) not all of the tarsiids for which we have calcaneal measurements were included in the MRP analysis, so Springer et al.’s tarsier phylogeny was grafted onto the tarsiid branch following computation of the MRP supertree; 2) Gunnell’s [39] notharctine phylogeny did not show sufficient taxonomic overlap with other trees to allow for notharctines’ resolved placement relative to non-notharctine primates, so we assumed notharctine monophyly and grafted Gunnell’s consensus tree (his Figure 5 [39]) onto the *Cantius abditus* branch, and 3) the species of *Chiropotes* that we measured was not present in Springer et al.’s tree, so that genus was collapsed into a single OTU in our tree. Finally, in order to reconstruct ancestral character states on 1) a tree that would be consistent with the hypothesis of a plesiadapiform-dermopteran clade [68], [69], and 2) another tree that would be consistent with a closer relationship of carpolestid plesiadapiforms to primates than to plesiadapid plesiadapiforms [15], we also modified the primary supertree by 1) moving plesiadapiforms to join dermopterans in an arrangement matching that proposed by Beard [69], and 2) moving *Carpolestes simpsoni* to be the sister group of living primates to the exclusion of all other euarchontans, with all other relationships remaining consistent with the primary supertree.

To convert the resulting supertrees into time-scaled phylogenies, we used the divergence times that Springer et al. [49] calculated using independent rates and soft bounds (their Text S2.4). The *Ptilocercus*-*Tupaia* divergence was not estimated in Springer et al.’s analysis; we placed this split at 61.8 Ma (i.e., the average of the mean divergence estimates calculated by Janečka et al. [50] and Roberts et al. [70]). Ghost lineages were minimized by placing extinct clades along stem lineages at successive 1 Ma intervals, working down from crown nodes. Within extinct clades, internodes were separated by 1 Ma unless adjacent sister taxa were geologically older. In the trees that recovered omomyiforms as paraphyletic with respect to tarsiids, the branch connecting *Necrolemur* to Tarsiidae was placed at 46 Ma because there is compelling fossil evidence that closer relatives of tarsiers were already present at ∼45 Ma (i.e., *Tarsius eocaenus* [71]). In order to evaluate whether a more recent haplorhine-strepsirrhine divergence (as implied by the molecular slowdown identified by Steiper and Seiffert [57]) had an impact on the reconstructed pattern of calcaneal evolution, we provided one additional modification of the primary supertree by adjusting the age of the primate crown node to reflect the ages of the oldest primate fossils (*Teilhardina* and *Cantius*) rather than the molecular divergence dates provided by Springer et al. [49]; divergence dates for more nested primate clades were the same as in the primary supertree.

Using the trees described above as input, three different sets of PGLS regressions were performed to determine how ankle elongation scales with body mass: 1) ln-transformed distal/total calcaneal length v. ln-transformed body mass as estimated from the cuboid facet area, 2) ln-transformed absolute distal calcaneal length v. ln-transformed body mass as estimated from the cuboid facet area, and 3) ln-transformed proximal calcaneal length v. ln-transformed body mass as estimated from the cuboid facet area. PGLS regressions in *caper* [72] employed the phylogenetic scaling parameter lambda (λ), a constant by which internal branch lengths are multiplied (λ of 0 would change all internal branch lengths to a length of 0). If trait evolution is well-modeled by Brownian motion, there will be a strong correlation between trait differences and branch lengths, and λ will approach 1.0. A λ value of 0 indicates that there is no correlation between trait values and branch lengths. In *caper* [72], other scaling parameters are available (δ, which adjusts overall path lengths, and κ, which adjusts individual branch lengths), but employing multiple branch length transformations simultaneously renders interpretation difficult; as such here we have only allowed λ to vary, and δ and κ were fixed as 1.

Phylogenetic ANOVA was used to assess whether significant among group variance in residual elongation values (Tables 1–2, Res A-B), exists for three different behavioral groups. The categorization for each taxon was determined through literature references [25], [72]–[77]. We treated animals that used acrobatic leaping behaviors from vertical or horizontal supports as a behavioral group, those that use primarily quadrupedal behaviors as another, and those that were slow-climbers or mainly terrestrialists as a third. We did not include animals that are committed to quadrumanual suspension (e.g., sloths, colugos, and presumably subfossil “sloth-lemurs”) in our analyses due to uncertainty about the demands that such behaviors place on the postcranium. Assignment of behavioral categories is described in more detail in **Results**. We report F statistics and P-values for these analyses. Post hoc comparisons were also executed. A sequential Dunn-Šidák adjustment to α = 0.05 was used to determine significance of P-values.

We used *BayesTraits* 1.1B to calculate means and 95% HPDs for ancestral states of both ln estimated body mass and the ratio of distal calcaneal segment length versus total calcaneal length. Mean values for each variable were used as the input data for each of the 117 OTUs in the primary supertree. Before reconstructing ancestral states, we ran several model tests to determine whether there were directional trends in the data given the input topologies, and whether inclusion of phylogenetic scaling parameters improved the likelihood of the reconstructions. For each tree, we ran two independent MCMC chains (10,050,000 iterations) for each of the following combinations: 1) non-directional model (model “A”, constant variance random-walk) with no scaling parameters; 2) non-directional model with parameter δ; 3) non-directional model with parameter κ; 4) non-directional model with parameter λ; 5) directional model (“model B”) with no scaling parameters; 6) directional model with parameter δ; 7) directional model with parameter κ; 8) directional model with parameter λ. The RateDev value was individually tuned for each analysis to achieve acceptances between 20–40%. The first 50,000 iterations were discarded from each chain as “burnin”, and the traces and mean log-likelihoods from the two independent chains were compared in Tracer 1.5 [78] to ensure convergence. Models for reconstruction of ancestral states were chosen by comparing harmonic means and averages of the mean log-likelihoods from each MCMC chain; i.e., models incorporating scaling parameters were only used for final reconstructions of ancestral states if the inclusion of a phylogenetic scaling parameter provided a log-likelihood that was 1.0 units greater than analyses that included no scaling parameters [79]. Ancestral reconstructions on each tree were based on two independent MCMC chains of 20,050,000 iterations (first 50,000 discarded as burnin, with the DataDev value tuned to achieve acceptances between 20–40%), using the distributions for the selected phylogenetic scaling parameters that were calculated in the previous step. Mean values and posterior densities for each reconstruction were taken from the combined results of the two independent MCMC runs.

## Results

### Allometry of the Earliest Euprimates

**Figure 3:**
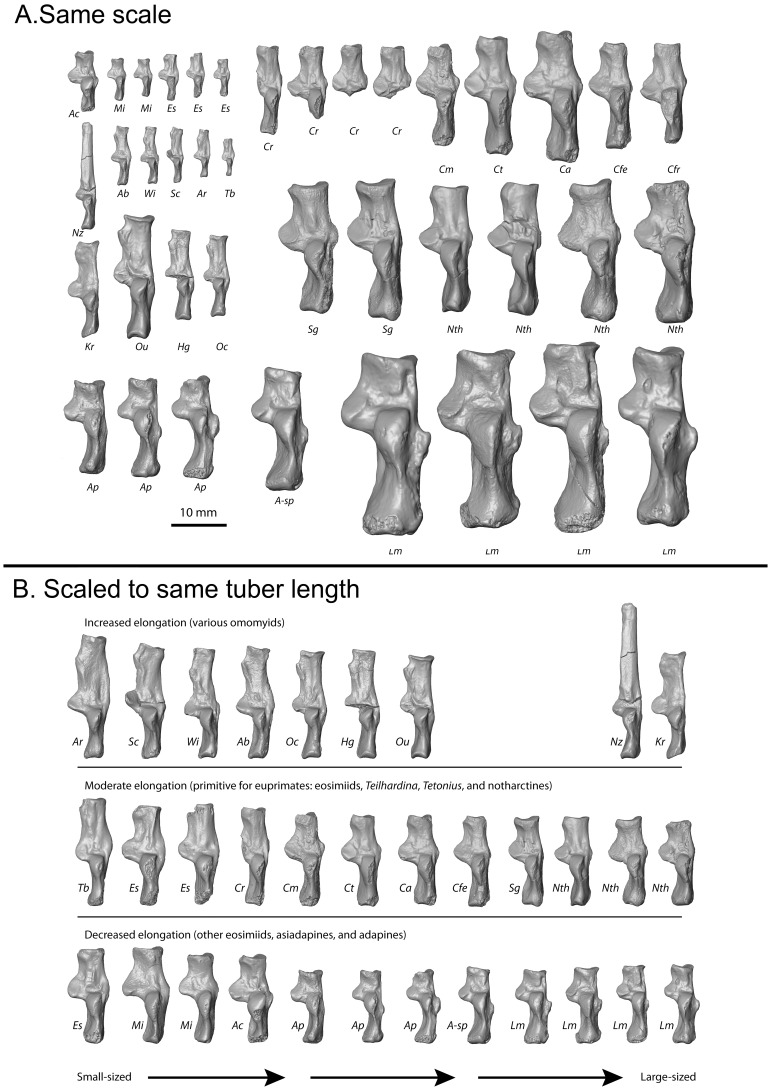
Relevant fossil calcanei exhibit a diversity of sizes and proportions. **A**, All relevant euprimate fossil (but not subfossil) genera measured and analyzed in this study are depicted at the same scale. **B**, the same taxa are depicted scaled to proximal segment length. The row corresponds to the scaling relationship of the taxa while the left-right position corresponds to body size. Note the left specimens (smaller) have relatively longer calcanei than the right speciments (larger). Abbreviations and specimen numbers (with numbers applying left to right; “*R”* stands for “reversed”): Ac, *Asiadapis cambayensis* (GU 760); Mi, *Marcgodinotius indicus* (GU 709,710); *Eosimias sinensis* (IVPP 12313*R*,12280*R*,11851); Cr, *Cantius ralstoni* (UF 252980; UM 79150; UM SLC VC misc6; CAB12–0209); Cm, *Cantius mckennai* ((USGS 5897*R*); Ct, *Cantius trigonodus* (USGS 21829); Ca, *Cantius abditus* (USGS 6783*R*); Cfe, *Copelemur feretutus* (USGS 21828*R*); Cfr, *Cantius frugivorus* (USGS 21781*R*); Nz, *Necrolemur zitteli* (A/V 637); Ab, *Absarokius sp.* (UCM 67907*R*); Wi, *Washakius indicus* (AMNH 88824); Sc, *Shoshonius cooperi* (CM69765); Ar, *Arapahovius gazini* (UCM 67850*R*); Tb, *Teilhardina belgica* (IRSNB 16786–03*R*); Kr, *Komba robustus* (KNM-SO 1364); Ou, *Ourayia uintensis* (SDSN 4020–60933); Hg, *Hemiacodon gracilis* (AMNH 12613); Oc, *Omomys carteri* (UCM 67678); Sg, *Smilodectes gracilis* (AMNH 131766*R*, 131774); Nth, *Notharctus tenebrosus.* (AMNH 11474*R*, 129382*R*, 131763*R*, 13766); Ap, *Adapis parisiensis* (NMB QE741*R*, QE644*R*, QE779); A-sp, *Adapis sp.* NMB QE 530; Lm, *Leptadapis magnus* (NMB QF421*R*, QE830*R*, QW 1676, QE604).

In 2007, we collected an isolated calcaneus that we attribute to the notharctine adapiform *Cantius ralstoni* UF 252980 (Fig. 3) based on size and morphology (see Fig. S1) from the Cabin Fork region [52], [55], [80], [81] of the Bighorn Basin, Wyoming. It is the first proximodistally complete specimen known for the species and is the oldest known *Cantius* specimen for which calcaneal elongation can be calculated. It is also the oldest known adapiform and potentially the oldest known stem strepsirrhine for which elongation can be calculated. It is thus notable that this specimen has the highest elongation ratio of any measureable *Cantius*. See Table S1 in File S1 for data. The new calcaneus attributed to *Cantius ralstoni* also has the smallest cuboid facet area of all specimens measured for *Cantius*, indicating a correspondingly small body size (Table S1 in File S1).

The coincidence of small size and high elongation in this specimen suggests an inverse correlation in these parameters among *Cantius*. Plotting body mass against calcaneal elongation (Fig. 4) indeed shows a significant inverse correlation for *Cantius* (ordinary least squares: ln [DL/TL] = −0.077*ln[BM]+ −0.343; p = 0.0012, n = 16).

This finding establishes a reasonable expectation that absolute size may explain calcaneal elongation among taxonomically larger groupings of notharctines. When *Notharctus* and *Smilodectes* are added to this sample, the correlation remains significant while the slope and intercept do not change significantly, as a result of overlapping 95% confidence intervals for both relationships (Table 3).

**Table 3:** Coefficients and confidence intervals for ordinary least squares regressions of ln(DL/TL) on estimated ln(BM) in extant and fossil taxa.

Regression Sample	abv	n	slope	slope SE	intercept	int SE	r	t	P(uncorr)	SLCI	SUCI	ILCI	IUCI	IR 1	IR 2
* *Cantius*	Ca	16	−0.077	0.019	−0.344	0.129	−0.73	−4.0	0.001	−0.118	−0.036	−0.619	−0.068	−0.051	−0.046
*Notharctus*	No	11	−0.058	0.037	−0.445	0.286	−0.47	−1.6	0.147	−0.141	0.024	−1.082	0.192	−0.003	−0.002
*Notharctines	Nn	27	−0.045	0.009	−0.559	0.068	0.47	−4.7	0.000	−0.064	−0.025	−0.699	−0.419	−0.007	−0.008
*Asiadapines	As	6	−0.077	0.024	−0.499	0.120	−0.85	−3.2	0.033	−0.139	−0.015	−0.807	−0.191	−0.206	−0.201
*Cantius* &asiadapines	CaAs	22	−0.006	0.009	−0.833	0.060	−0.13	−0.6	0.555	−0.025	0.014	−0.958	−0.708	0.029	0.020
* *Cantius* &*Teilhardina*	CaT	20	−0.076	0.005	−0.354	0.029	−0.97	−16.6	0.000	−0.085	−0.066	−0.414	−0.294	−0.049	−0.044
- Notharctines &asiadapines	NnAs	34	−0.013	0.006	−0.788	0.043	−0.36	−2.1	0.040	−0.026	−0.001	−0.875	−0.701	0.013	0.005
*Notharctines &*Teilhardina*	NonT	31	−0.065	0.004	−0.407	0.030	−0.94	−14.9	0.000	−0.074	−0.057	−0.468	−0.346	−0.022	−0.019
* *Cantius* &anaptomorphines	CaAn	22	−0.079	0.005	−0.328	0.031	−0.96	−15.9	0.000	−0.090	−0.069	−0.391	−0.264	−0.053	−0.047
*Notharctines &anaptomorphines	NnAn	33	−0.069	0.004	−0.380	0.029	−0.94	−15.8	0.000	−0.078	−0.060	−0.440	−0.320	−0.024	−0.020
Anaptomorphines	An	6	0.000	0.041	−0.646	0.166	0.00	0.0	0.995	−0.105	0.106	−1.073	−0.219	0.264	0.254
*Omomyines	Om	10	−0.052	0.010	−0.344	0.057	−0.88	−5.2	0.001	−0.075	−0.029	−0.473	−0.215	0.146	0.146
*North AmericanEocene primates	NAE	44	−0.083	0.008	−0.260	0.050	−0.86	−10.7	0.000	−0.098	−0.067	−0.361	−0.160	−0.012	−0.006
*Galagidae	Ga	15	−0.065	0.004	0.026	0.027	−0.97	−15.0	0.000	−0.074	−0.056	−0.031	0.083	0.417	0.419
*Lorisidae	Lr	13	−0.094	0.023	−0.380	0.139	−0.78	−4.1	0.002	−0.145	−0.044	−0.682	−0.077	−0.226	−0.217
* *Microcebus* & *Mirza*	McMr	6	−0.085	0.014	−0.125	0.066	−0.95	−6.0	0.004	−0.122	−0.049	−0.295	0.045	0.101	0.108
*Cheirogaleus*	Ch	5	−0.039	0.062	−0.502	0.341	−0.34	−0.6	0.571	−0.211	0.133	−1.448	0.444	0.092	0.090
* *Hapalemur*	Ha	13	−0.065	0.013	−0.336	0.095	−0.84	−5.2	0.000	−0.092	−0.038	−0.542	−0.130	0.053	0.056
*Lemurids	Le	25	−0.076	0.010	−0.248	0.078	−0.84	−7.5	0.000	−0.097	−0.055	−0.408	−0.087	0.050	0.056
*Lemurids & cheirogaleids	LeCh	36	−0.088	0.005	−0.159	0.037	−0.94	−16.7	0.000	−0.099	−0.078	−0.234	−0.084	0.043	0.051
*Lemuriforms	Lemf	50	−0.086	0.006	−0.179	0.043	−0.90	−14.3	0.000	−0.098	−0.074	−0.265	−0.094	0.045	0.052
*Indriids	Ind	8	0.081	0.015	−1.527	0.117	0.91	5.5	0.001	0.046	0.115	−1.804	−1.250	0.024	−0.003
*Tarsius*	Trs	8	−0.105	0.056	0.248	0.279	−0.61	−1.9	0.108	−0.236	0.027	−0.411	0.906	0.320	0.331
*Platyrrhines	Plat	35	−0.032	0.010	−0.668	0.077	−0.47	−3.2	0.003	−0.052	−0.012	−0.824	−0.512	−0.017	−0.021
*Cercopithecoidea	Cc	23	−0.071	0.027	−0.361	0.245	−0.49	−2.6	0.017	−0.128	−0.014	−0.869	0.147	−0.020	−0.016
*Hominoidea	Hm	12	−0.137	0.033	0.170	0.346	−0.80	−4.2	0.002	−0.208	−0.065	−0.593	0.932	−0.012	0.006
*Parapithecidae	Par	6	−0.136	0.013	−0.070	0.082	−0.98	−10.7	0.000	−0.169	−0.103	−0.280	0.139	−0.249	−0.231
*Euprimates	Eu	253	−0.103	0.006	−0.121	0.047	−0.71	−16.2	0.000	−0.114	−0.088	−0.225	−0.040	−0.031	−0.021
Scandentia	Sc	8	0.014	0.015	−1.025	0.069	0.34	0.9	0.410	−0.023	0.050	−1.189	−0.862	−0.010	−0.023
Plesiadapiforms	Pls	6	0.008	0.022	−1.152	0.114	0.17	0.0	0.744	−0.050	0.066	−1.445	−0.858	−0.181	−0.194
Sundatheria	Sun	10	0.008	0.007	−1.001	0.001	0.37	1.1	0.287	−0.008	0.023	−1.004	−0.998	−0.031	−0.043
Non-euprimates	NnEu	15	0.012	0.016	−1.080	0.083	0.20	0.8	0.459	−0.022	0.047	−1.258	−0.901	−0.073	−0.086

* Significant correlation between estimated ln(BM) and ln(DL/TL).

-Marginally significant or marginally non-significant.

Abbreviations: abv, sample abbreviation; n, sample size; SE, standard error; int, intercept; r, correlation coefficient; t, student’s t-value; SLCI, slope lower 95% confidence interval; SUCI, slope upper 95% confidence interval; ILCI, intercept lower 95% confidence interval; IUCI, intercept upper 95% confidence interval; P(uncorr), Probability of no correlation; IR 1, intercept residual from slope v. regression equation 1 [including indriids: (intercept) = −7.978 (slope) +0.908]; IR 2, intercept residual from slope v. regression equation 2 [excluding indriids: (intercept) = −7.77 (slope) +0.89].

The inter-generic robusticity of this relationship justifies evaluating the effect of including other primitive euprimates. Among omomyiforms, *Teilhardina* is well established as the most basal member of the group e.g., [21], [52], [82], and also the only to co-occur with the earliest species of *Cantius*. Adding data for *Teilhardina* results in no significant change to either coefficient relative to the line including *Cantius*, *Notharctus,* and *Smilodectes* (Fig. 4; Table 3; ordinary least squares (OLS): ln [DL/TL] = −0.0654*ln[BM]+ −0.407; p<0.0001, n = 31). Alternatively, adding data from various other early primates does in fact change the relationship in significant ways. Most notably, adding data on asiadapines from India [37], either significantly reduces the estimated slope or results in non-significant correlations (Table 3). On the other hand if asiadapines are treated as a separate subsample, regression analysis yields a strong correlation with a slope similar to that for the early Eocene North American primates, but with a lower mean intercept estimate as compared to their North American counterparts (Table 3). Furthermore, treating omomyine omomyiforms separately also yields a line with a strong correlation and similar slope, but elevated intercept relative to the early Eocene line. These initial regressions were run with data on individuals, but most relationships remain significant when using species mean values (Table 4). Phylogenetic Generalized Least Squares (PGLS) regression on species means for *Teilhardina*, *Cantius, Notharctus,* and *Smilodectes* results in a significant relationship with no significant differences in regression coefficients compared to OLS regression. The equation resulting from the PGLS regression is ln [CDL/CTL] = (−0.070 to 0.072)*ln[BM] - (0.38 to 0.37), n = 8. Value ranges represent variance due to phylogeny used, not confidence intervals (which include −0.065, the value from the ordinary least squares regression): see Table 5 for standard error on coefficient estimates.

**Table 4:** Coefficients and confidence intervals for ordinary least squares regressions of ln(DL/TL) on estimated ln(BM) for taxon means.

regression description	abv	n	slope	slope SE	intercept	int SE	r	t	P(uncorr)	SLCI	SUCI	ILCI	IUCI
*-Cantius*	Ca	5	−0.1046	0.03358	−0.16157	0.222	−0.87399	−3.115	0.05267	−0.198	−0.011	−0.778	0.455
*Notharctus*	No	2	na	na	na	na	na	na	na	na	na	na	na
*Notharctines	Nn	7	−0.057	0.01583	−0.47227	0.11	−0.84938	−3.599	0.015568	−0.096	−0.018	−0.742	−0.202
*Asiadapines	As	2	na	na	na	na	na	na	Na	na	na	na	na
*Cantius* & asiadapines	CaAs	7	0.00332	0.028	−0.416	0.159	0.053698	0.1203	0.74667	−0.066	0.072	−0.804	−0.027
* *Cantius* & *Teilhardina*	CaT	6	−0.0773	0.02762	−0.88786	0.173	−0.97722	−9.21	0.00077219	−0.148	−0.006	−1.333	−0.443
-Notharctines & asiadapines	NnAs	9	−0.0116	0.019	−0.79938	0.114	−0.24825	−0.678	0.51952	−0.055	0.032	−1.062	−0.537
*Notharctines & *Teilhardina*	NonT	9	−0.081	0.011	−0.310	0.067	−0.932	−6.804	0.00015299	−0.107	−0.056	−0.464	−0.156
* *Cantius* & anaptomorphines	CaAn	8	−0.0858	0.01019	−0.28429	0.06	−0.9602	−8.42	0.016271	−0.110	−0.062	−0.425	−0.144
*Notharctines & anaptomorphines	NnAn	8	−0.0773	0.00801	−0.32685	0.05	−0.95971	−9.66	1.10E-05	−0.096	−0.058	−0.446	−0.208
Anaptomorphines	An	3	0.00719	0.09844	−0.6767	0.419	0.072887	0.0731	0.95356	−0.416	0.431	−2.480	1.127
*Omomyines	Om	2	−0.0553	0.01112	−0.33373	0.061	−0.9279	−4.978	0.0076093	−0.197	0.086	−1.113	0.446
*North American Eocene primates	NAE	16	−0.0838	0.01304	−0.24704	0.078	−0.8641	−6.424	1.59E-05	−0.112	−0.056	−0.414	−0.080
*Galagidae	Ga	4	−0.0654	0.00689	0.027312	0.002	−0.98887	−9.399	0.011132	−0.087	−0.044	0.022	0.033
*Lorisidae	Lr	5	−0.0871	0.03843	−0.4369	0.232	−0.79458	−2.267	0.10826	−0.194	0.020	−1.080	0.206
*Microcebus* & *Mirza*	McMr	2	na	na	na	na	na	na	Na	na	na	na	na
*Cheirogaleus*	Ch	2	na	na	na	na	na	na	Na	na	na	na	na
*Hapalemur*	Ha	2	na	na	na	na	na	na	Na	na	na	na	na
*Lemurids	Le	5	−0.0788	0.01343	−0.23237	0.102	−0.95909	−5.868	0.0098712	−0.116	−0.042	−0.516	0.052
*Lemurids & cheirogaleids	LeCh	11	−0.0911	0.01074	−0.14755	0.073	−0.94279	−8.483	1.38E-05	−0.115	−0.067	−0.310	0.015
*Lemuriforms	Lemf	14	−0.0859	0.01372	−0.18906	0.097	−0.87489	−6.258	4.21E-05	−0.116	−0.056	−0.398	0.020
Indriids	Ind	3	0.07606	0.01596	−1.4866	0.016	0.97774	4.6602	0.13457	0.007	0.145	−1.555	−1.419
*Tarsius*	Trs	3	−0.1309	0.12643	0.37934	0.632	−0.71932	−1.036	0.48891	−0.675	0.413	−2.341	3.100
*Platyrrhines	Plat	19	−0.0243	0.01099	−0.71205	0.083	−0.47317	−2.215	0.040741	−0.047	−0.001	−0.887	−0.537
Cercopithecoidea	Cc	13	−0.0797	0.04098	−0.26949	0.364	−0.50607	−1.946	0.077637	−0.169	0.010	−1.062	0.523
*Hominoidea	Hm	7	−0.1166	0.03989	−0.02617	0.408	−0.79415	−2.922	0.032936	−0.214	−0.019	−1.024	0.972
Parapithecidae	Par	2	na	na	na	na	na	na	na	na	na	na	na
*Euprimates	Eu	98	−0.0967	0.00907	−0.15368	0.067	−0.73606	−10.65	5.89E-18	−0.115	−0.079	−0.287	−0.021
Scandentia	Sc	2	na	na	na	na	na	na	na	na	na	na	na
Plesiadapiforms	Pls	6	0.00786	0.02246	−1.1518	0.114	0.17238	0.0297	0.74399	−0.050	0.066	−1.445	−0.858
Sundatheria	Sun	3	0.00647	0.00401	−0.99285	0.021	0.85013	1.6145	0.35305	−0.011	0.024	−1.085	−0.901
Non-euprimates	NnEu	8	−0.0018	0.02498	−1.0376	0.136	−0.02866	−0.07	0.9463	−0.061	0.057	−1.359	−0.716

* Significant correlation between estimated ln(BM) and ln(DL/TL).

−marginally significant or non-significant.

Abbreviations: abv, sample abbreviation; n, sample size; SE, standard error; int, intercept; r, correlation coefficient; t, student’s t-value; SLCI, slope lower 95% confidence interval; SUCI, slope upper 95% confidence interval; ILCI, intercept lower 95% confidence interval; IUCI, intercept upper 95% confidence interval; P(uncorr), Probability of no correlation.

**Table 5:** Results of PGLS regressions of distal elongation index [ln(DL/TL)] on ln estimated body mass.

Groups	Tree	λ	Adj R^2^	slope	SE	Int	SE	t-value	P(>t)	RSE (DF)	F-value (DF)	P
*Cantius*	1–3	0.000	0.73	−0.108	0.032	−0.136	0.207	−3.43	0.042	0.111(3)	11.77(2,3)	0.038
Notharctines	1–3	0.000	0.69	−0.069	0.019	−0.391	0.128	−3.62	0.015	0.116(5)	13.08(2,5)	0.010
(a) Notharctines+*Th*	1,3	0.000	0.93	−0.070	0.006	−0.381	0.045	−10.04	<0.0001	0.055(6)	100.9(2,6)	<0.0001
(a) Notharctines+*Th*	2	0.000	0.94	−0.072	0.007	−0.372	0.043	−10.57	<0.0001	0.089(6)	111.7(2,6)	<0.0001
Adap.+Omo.	1	0.924	0.11	−0.040	0.021	−0.532	0.139	−1.88	0.075	0.318(20)	3.54(2,20)	0.048
Adap.+Omo	2	0.879	0.23	−0.056	0.021	−0.454	0.122	−2.67	0.015	0.362(20)	7.14(2,20)	0.0045
Adap.+Omo	3	0.905	0.19	−0.047	0.019	−0.496	0.116	−2.45	0.024	0.313(20)	5.99(2,20)	0.0092
Extant Primates	1,3	0.976	0.30	−0.092	0.016	−0.119	0.162	−5.78	<0.00001	0.309(74)	33.43(2,74)	<0.00001
Extant Primates	2	0.973	0.30	−0.092	0.016	−0.123	0.148	−5.79	<0.00001	0.311(74)	33.53(2,74)	<0.00001
Euprimates *sin* (a)	1	0.953	0.26	−0.072	0.012	−0.375	0.102	−5.85	<0.00001	0.303(93)	34.24(2,93)	<0.00001
Euprimates *sin* (a)	2	0.964	0.25	−0.072	0.012	−0.366	0.081	−5.69	<0.00001	0.329(93)	32.45(2,93)	<0.00001
Euprimates *sin* (a)	3	0.955	0.27	−0.072	0.012	−0.367	0.105	−6.02	<0.00001	0.304(93)	36.29(2,93)	<0.00001
All Euprimates	1	0.953	0.24	−0.066	0.011	−0.397	0.096	−5.78	<0.00001	0.298(100)	33.52(2,100)	<0.00001
All Euprimates	2	0.951	0.27	−0.069	0.011	−0.383	0.071	−6.15	<0.00001	0.311(100)	27.85(2,100)	<0.00001
All Euprimates	3	0.952	0.27	−0.068	0.011	−0.382	0.098	−6.17	<0.00001	0.295(100)	38.01(2,100)	<0.00001
Extant Prosimians	1,3	0.871	0.35	−0.112	0.027	0.062	0.213	−4.30	0.0002	0.316(31)	18.48(2,31)	<0.00001
Extant Prosimians	2	0.860	0.36	−0.114	0.026	0.054	0.203	−4.33	0.0001	0.323(31)	18.78(2,31)	<0.00001
Extant Anthropoids	1–3	0.872	0.29	−0.071	0.017	−0.394	0.157	−4.24	0.0001	0.305(41)	17.99(2,41)	<0.00001
All Prosimians	1	0.902	0.21	−0.066	0.017	−0.381	0.123	−3.94	0.0002	0.324(54)	15.57(2,54)	<0.00001
All Prosimians	2	0.872	0.26	−0.072	0.016	−0.362	0.096	−4.53	<0.0001	0.338(54)	20.52(2,54)	<0.00001
All Prosimians	3	0.903	0.25	−0.071	0.016	−0.360	0.124	−4.37	<0.0001	0.322(54)	19.11(2,54)	<0.00001
All Anthropoids	1–3	0.862	0.41	−0.071	0.012	−0.426	0.061	−5.68	<0.00001	0.200(44)	32.22(2,44)	<0.00001

Note that when **λ** is 0.000, regressions are equivalent to TIPS data (internal branch lengths = 0). Column heading abbreviations: Adj, adjusted; DF, degrees of freedom; Int, y-intercept; P, probability; RSE, Residual Standard Error. Taxon abbreviations: *Th, Teilhardina*. Trees:1, MP supertree with molecular divergence dates from Springer et al. (2012); 2, MP supertree with fossil dictated early branch lengths; 3, adapiform-haplorhine constraint supertree.

### Intrageneric Allometry in Modern Primates

Patterns similar to those described above have been noted among extant primates at the family level using species means for calcaneal elongation and body mass [33]. We re-assessed these patterns, modifying the approach slightly to match the structure of our comparisons among fossil euprimates. We specifically included samples representing closely related species that are either currently classified in the same genus, or that were, at one time, considered members of the same genus on the basis of sister taxon relationships that are still considered valid. Some of these “generic” groups of species therefore require explanation. Our “*Hapalemur*” group includes *Hapalemur griseus* and *Prolemur simus* [83]: *P. simus* is typically classified as *Hapalemur* [84] and is consistently reconstructed as a very recently diverged sister taxon [49]. The “*Galago*” group includes species classified in the genera *Otolemur*, *Galagoides* and *Euoticus* by recent authors [49], [83]. However, many authors previously classified some or all of these species in *Galago* [83], [85], [86]. Furthermore, the monophyly and inter-relationships of these genera are in flux (see explanation in **Results**). The “*Microcebus*” group includes species typically included in *Microcebus* as well as *Mirza coquereli*, which is sometimes also recognized as a species of *Microcebus* [87]; again, *Microcebus* and *Mirza* are almost certainly sister taxa [49], [83]. Figure 5 and Tables 3 and 4 give complete listings of comparisons and results. We also made broader intra-familial comparisons (Table 4). We found a significant pattern of inverse correlation between calcaneal elongation and body mass for all groups compared except *Cheirogaleus*, *Tarsius*, non-primate euarchontans, and Indriidae.

**Figure 6:**
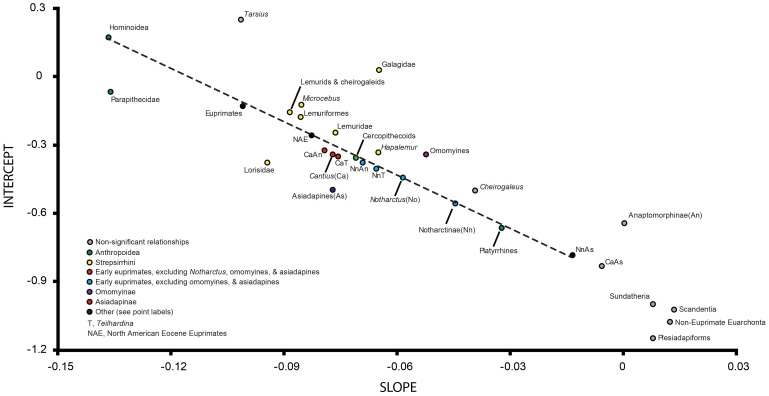
Comparison of ordinary least squares (OLS) lines by plotting slopes and intercepts. When using ordinary least squares, it is difficult to define a natural group to which to limit a sample for a scaling relationship. We dealt with this in several ways: 1) by starting with small (genus level) groups, and adding sister taxa until the slope and/or intercept of the line changed significantly, including the loss of a significant relationship. 2) For extinct taxa, we considered both phylogenetic proximity (not just monophyly). Our approach yielded a large number of regression equations (Table 2), which are difficult to compare with one another since changes in slope can be expected to yield changes in intercept. Therefore, we graphically compare the regression equation estimates by using slope of a relationship as the covariate and intercept as a dependent variable. This shows an expected relationship: more negative slopes have predictably higher intercepts. Fitting a line to this relationship we compare intercepts (or relative calcaneal elongation) as residuals from this line. This allows us to compare line position when methods like ANCOVA are not supported due to differing slopes of lines of interest. What can be seen is that parapithecids, asiadapines and lorisids have regression equations with the lowest residuals, Eocene taxa tend to have slightly negative residuals, lemuriforms have slightly positive residuals, omomyines have higher residuals, and galagos have the highest residuals. The tarsier relationship is non-significant (as is that for all gray points) so its position is not technically meaningful. However, the non-significant relationship for *Tarsius* appears mainly a result of small sample size (likely) given the high slope, in contrast to other non-significant relationships (“anaptomorphines,” scandentians, etc.) which have slopes close to zero. This plot presents data consistent with other ways of looking at body-size scaled levels of calcaneal elongation used in this study and suggests on average that early Eocene primates had lower levels of calcaneal elongation than extant lemuriforms.

Figure 6 provides a graphical representation of the variance in slope and intercept for the different subsamples analyzed. We do not suggest that regression analysis is an appropriate way of analyzing these data, nor do we imply that these subsamples and their coefficients are independent data points. We simply use this plot to help illustrate how changing subsample composition changes slope and intercept. It is clear that subsamples with a steeper slope tend to have a higher intercept. Visualizing the data in this way helps to reveal groups that have more elongation, while controlling for slope differences. Generally speaking, extant groups exhibit higher intercepts for the slope of their relationship (indicating greater average calcaneal elongation) than do fossil groups. A clear exception is the low residual intercepts of lorisids.

### The Calcaneal Allometry of All Primates

**Figure 7:**
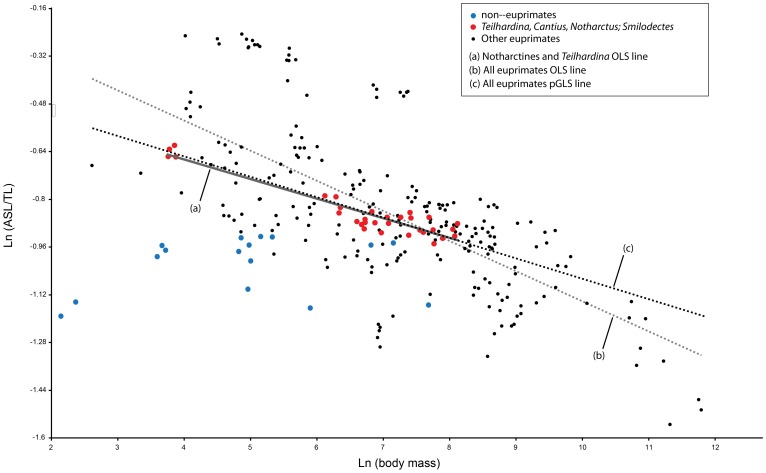
Comparison of ordinary least squares (OLS) and phylogenetic generalized least squares lines fit to an “all primate” sample. Adding data from all extant primate groups leads to a much steeper ordinary least squares regression slope (*b*) than given by analyzing smaller samples of closely related taxa (e.g., *a*). However, PGLS style regression using the *caper* package of R shows that phylogenetic autocorrelation of values has little affect for small samples of closely related taxa (low values of λ in Table 5) meaning that OLS and PGLS give nearly identical results. However, phylogenetic autocorrelation has a strong effect in larger samples (higher λ values in Table 5). The maximum likelihood PGLS regression equations for large samples (*c*) thus show a much different slope than the OLS equations for these samples. The PGLS slope and intercept are instead much closer to that for small samples.

Although comparisons of close relatives reveal significant correlations, adding more distantly related groups and utilizing bigger sample sizes begins to blur the relationship. To assess the presence of a “fundamental” allometry in calcaneal elongation, we assembled a comprehensive sample representing all major primate groups. OLS regression shows a significant inverse correlation between calcaneal elongation and body mass (Table 4; ordinary least squares: ln [CDL/CTL] = (−1)*(0.103)*ln[BM] - (0.12), n = 260). One might question whether this slope (Fig. 7) is more a function of coincidental phylogenetic autocorrelation or clade shifts [24] in elongation and body mass (by clade shift, we mean morphological differences established early in the evolution a clade and retained to some degree by most later-occurring members of the clade). That is, the very highest elongations are represented mainly by small-bodied galagos and tarsiers, while the low elongations are represented mainly by the large bodied apes. A PGLS regression on species means was used to assess whether this relationship held while accounting for large scale clade offsets as might be produced by phylogenetic autocorrelation in trait values. The result of this analysis is still a highly significant inverse correlation that in fact, matches the slope and intercept of the Eocene taxa treated alone (PGLS regression: ln [CDL/CTL] = (−1)*(0.066–0.069)*ln[BM] - (0.39–0.38), n = 98. Value ranges represent variance due to cladogram used, not confidence intervals: see Table 5 for standard errors on coefficient estimates). Finally we ran a number of analyses representing different subgroups of the total sample (see Table 5 for full results). Overall, results show that 1) generally, sub-sampling does little to change slope (Table 5), and 2) the degree of phylogenetic co-variance of trait values is stronger in the larger, phylogenetically more diverse samples (see Table 5 for λ values).

The expectation of a consistent allometric pattern of calcaneal elongation assumes the presence of a tarsifulcrumating foot, which is ubiquitous among extant prosimians. This assumption is challenged by the observation that the metatarsifulcrumating foot occurs several times in anthropoid evolution. We therefore also separated our sample into extant prosimians and extant anthropoids and ran two additional PGLS regressions. We found that each clade still exhibited a strong significant correlation between calcaneal elongation and estimated mass (Table 5).

### Scaling of Individual Segments of the Calcaneus

To assess the contribution of individual components of the elongation index to leaping we regressed distal and proximal calcaneal length against estimated body mass (Table 6). Using the PGLS approach shows that as a group, euprimates have significant negative allometry of the distal segment (as shown by Moyà-Solà et al. [7]), and significant positive allometry of the proximal segment. Interestingly, there is evidence of stronger phylogenetic signal of distal segment length, and a poor body size correlation; while in contrast there is a weaker phylogenetic signal in the proximal segment length and a very strong correlation with body mass.

**Table 6:** Regression table giving PGLS regression results of ln calcaneal segment lengths with ln of estimated body mass.

Groups	Tree	λ	Adj R^2^	slope	SE	Int	SE	t-value	P(>t)	RSE (DF)	F-value (DF)	P
All Euprimates (DL)	1	0.991	0.57	0.256	0.022	0.332	0.197	11.63	<0.00001	0.625(100)	135.3(2,100)	<0.00001
All Euprimates (DL)	2	0.990	0.57	0.252	0.022	0.360	0.138	11.72	<0.00001	0.633(100)	137.3(2,100)	<0.00001
All Euprimates (DL)	3	0.991	0.56	0.247	0.022	0.364	0.204	11.45	<0.00001	0.627(100)	131.2(2,100)	<0.00001
All Euprimates (PL)	1	0.703	0.92	0.364	0.011	−0.140	0.078	34.33	<0.00001	0.233(100)	1178(2,100)	<0.00001
All Euprimates (PL)	2	0.838	0.91	0.371	0.011	−0.175	0.071	32.74	<0.00001	0.293(100)	1072(2,100)	<0.00001
All Euprimates (PL)	3	0.743	0.92	0.363	0.010	−0.153	0.081	35.26	<0.00001	0.233(100)	1243(2,100)	<0.00001

Column heading abbreviations: see table 4 legend. Other abbreviations: DL, distal segment length; PL, Proximal segment length.

Thus, as body mass increases, there is both a disproportionately smaller increase in length of the distal segment, and a disproportionately larger increase in length of the proximal segment, which together result in a correlation between body mass and elongation index.

### Behavioral Variance in Calcaneal Elongation

The foregoing analyses confirm that a large amount of variance in calcaneal elongation is related to body mass, *not* any simple behavioral category *per se*. We therefore assessed the behavioral significance of elongation differences with a method that takes this allometry into account. Specifically we took residuals from the allometric line describing the major variation in all euprimates (i.e., treated it as a line of subtraction) and used phylogenetic ANOVA (using the *caper* package of R [88]) to assess significant behavioral variance. Three behavioral categories were used: 1) vertical clinging & leaping and/or grasp-leaping (VCL/L), 2) arboreal quadrupedalism (AQ), and 3) slow-climbing/terrestrial (SC/T). We did not include taxa that are predominantly suspensory because we had no well-informed predictions for what pattern of elongation selection should favor for an animal that loads its limbs in tension. A phylogenetic ANOVA using PGLS allows for auto-correlation between trait values and phylogenetic distance, adjusting estimates of group means and their standard errors accordingly. We first used PGLS to estimate the common slope and intercept for all primates (which matches closely the slope of many “intrageneric” and “subfamilial” groups, including notharctines: Table 3, 4, 5) and then took the residuals for each species with respect to this line (Table 1). We ran three sets of ANOVAs: 1) on all extant primates in our sample; 2) on all anthropoids; 3) on all prosimians. We ran the prosimian analysis using three different trees due to an unconventional (and relatively poorly supported) position for the galagid *Euoticus elegantulus* recovered by Springer et al. [49]. In Springer et al.’s data set *E. elegantulus* was only sampled for 7% of sites, *G. matschiei* for 2%, and *G. demidoff* for 11%, while *G. senegalensis*, *O. crassicaudatus*, and *O. garnetti* were sampled for 70–80% of sites. They recovered *E. elegantulus* as the basal branching galagid, a result rarely recovered in other studies. Furthermore, the bootstrap support for monophyly of *Galago* and *Otolemur* to the exclusion of *Euoticus* was less than 50%. Prior to Springer et al.’s [44] finding, the field had begun to converge on the idea of a sister relationship between *E. elegantulus* and *G. senegalensis*, with *Otolemur* outside of this clade, and *G. demidoff* and others as more basally diverging yet [41], [89]–[92].

Analysis 1 revealed significant differences between the SC/T group and VCL/L and AQ groups (Table 7). However, the latter two groups are not significantly different. Analysis 2 of anthropoids showed a difference only between SC/T and AQ. Finally, the third set of analyses yielded significant differences among all prosimian behavioral groups when trees representing the most frequently supported position of *Euoticus* were used [41], [89]–[92]. However, when prosimian data were analyzed using the main supertree compiled for this paper and reflecting the less conventional position of *Euoticus*, we obtained results similar to those for Analysis 1 including all primates in which there is no difference between VCL/L and AQ (Table 7). To address directly Moyà-Solà et al.’s hypothesis that specifically distal length (instead of elongation index) relative to body mass does not correlate with the degree of leaping, we used a similar approach taking residuals from the line determined using PGLS (Table 6; “Res B” of table 1). The results of this approach were similar in most respects to those utilizing elongation residuals (Table 7).

**Table 7:** Phylogenetic ANOVA of calcaneal elongation residuals (see Table 1: Res A) and distal calcaneal length residuals (see Table 1: Res B) for extant and subfossil prosimian species means from PGLS line based on “all primate” sample including posthoc comparisons.

Data	grp	tree	λ	df	MS	F	P	SC/T v AQ	SC/T v VCL/L	AQ v VCL/L
Res A	All	1	0.991	63	0.055	11.6	<0.0001	−0.107 (−4.6/**0.0001**)*	−0.112 (−3.6/**0.0006**)*	−0.004 (−0.163/0.87)
Res B	All	1	0.990	63	0.275	2.47	0.07	−0.115 (−2.2/0.035)	−0.109 (−1.6/0.123)	0.007 (0.105/0.92)
Res A	Anth	1	0.960	33	0.036	4.22	0.012	−0.078 (−2.9/**0.007**)*	−0.036 (−1.0/0.323)	0.042 (1.32/0.195)
Res B	Anth	1	0.739	33	0.028	5.35	0.004	−0.085 (−1.9/0.055)	0.052 (1.06/0.298)	0.137 (3.14/**0.004**)*
Res A	Pros	1	0.993	27	0.027	30.16	<0.0001	−0.298 (−6.4/<**0.0001**)*	−0.340 (−7.6/<**0.0001**)*	−0.042 (−1.18/0.248)
Res B	Pros	1	1.000	27	0.258	6.17	0.002	−0.389 (−2.5/**0.018**)*	−0.525 (−3.15/**0.001**)*	−0.136 (−1.16/0.256)
Res A	Pros	2	1.000	27	0.00023	32.8	<0.0001	−0.279 (−6.2/<**0.0001**)*	−0.353 (−8.1/<**0.0001**)*	−0.074 (−2.46/**0.020**)*
Res B	Pros	2	1.000	27	0.00262	9.038	0.0003	−0.327 (−2.2/**0.037**)*	−0.582 (−4.0/**0.0004**)*	−0.254 (−2.54/**0.017**)*
Res A	Pros	3	0.993	27	0.0278	30.75	<0.0001	−0.282 (−6.1/<**0.0001**)*	−0.347 (−7.8/<**0.0001**)*	−0.065 (−2.13/**0.042**)*
Res B	Pros	3	1.000	27	0.317	7.58	0.0008	−0.334 (−2.18/0.038)	−0.548 (−3.72/**0.0009**)*	−0.214 (−2.14/0.041)

Column abbreviations: df, degrees of freedom; MS, mean squared error within groups from ANOVA; F, F-statistic for ANOVA; P, probability of significant between groups variance for ANOVA. For each cell of the post-hoc comparison first the difference between group means is given. Then in parentheses the t-value/p-value for a students paired sample t-test is given. Correction for multiple post hoc comparisons using a sequential Dunn-Šidák correction for k = 3 comparisons and α = 0.05 (Initial α′ = 1- (1 - α)^1/k = ^0.0169. If smallest P-value ≤0.0169, then for the second smallest P-value, α′ = 1- (1 - α) ^1/(k−1) = ^0.026. If the second smallest P-value ≤0.026, then α′ = α = 0.05 for last P-value). Asterisks denote a significant P-value.

### Ancestral State Reconstruction

**Figure 8:**
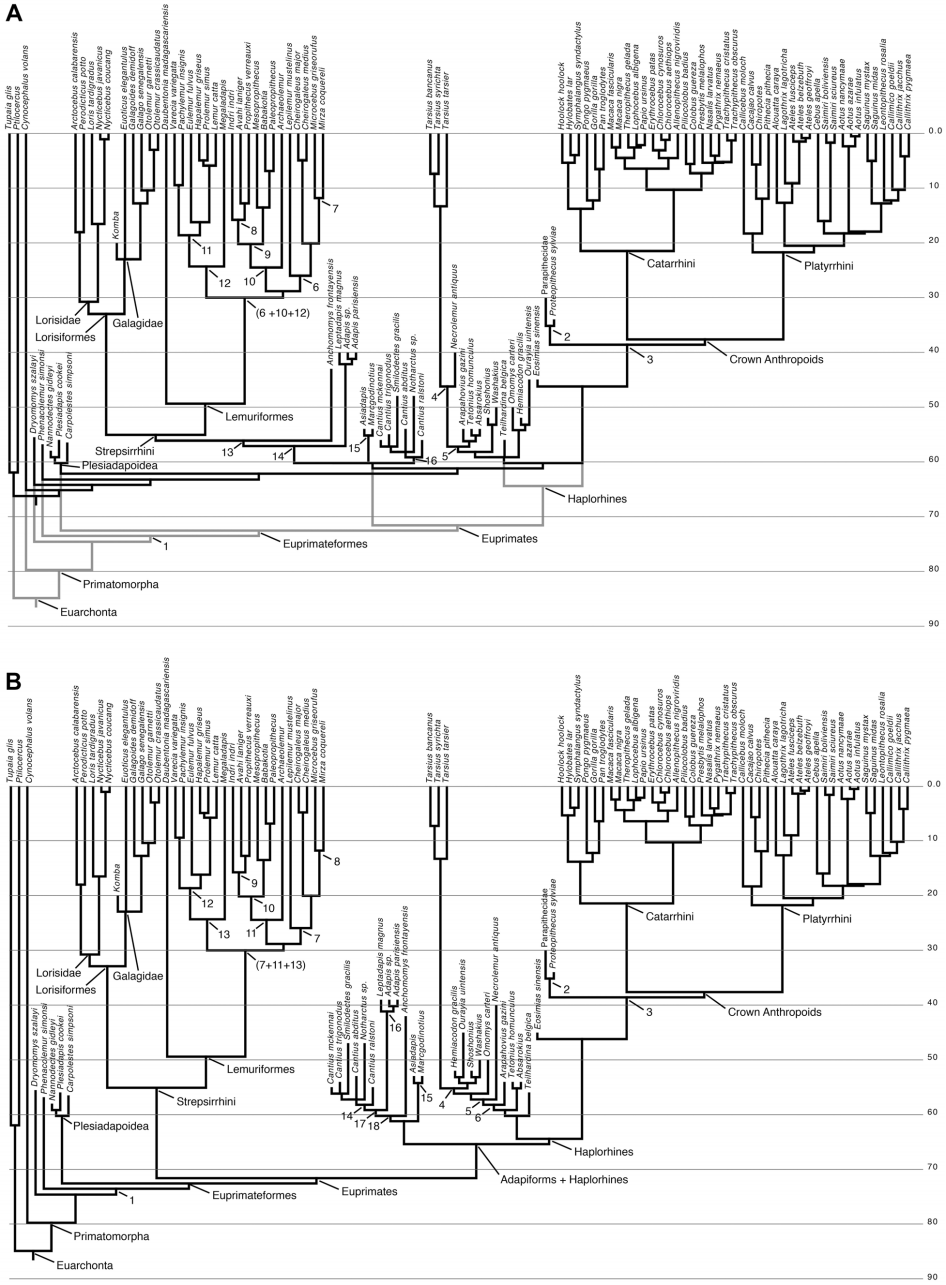
Representative trees for ASR state reconstruction. Part A shows trees 1 and 2 which differ in branch lengths towards the base of the tree only. Tree 1 has divergence dates for major extant clades set by molecular evidence. We institute minimum ghost lineages to incorporate fossils. Tree 2 has divergence dates set by fossil evidence when available such that the creation of ghost lineages is minimized even more. Node numbers of interest are given for reference with Table 8, and Figure 10. Part B represents tree 3 in which Eocene omomyiforms and adapids are treated as stem haplorhines [102], [108] which we consider the most substantially different, yet potentially correct alternative hypothesis for euprimate relationships.

**Figure 9:**
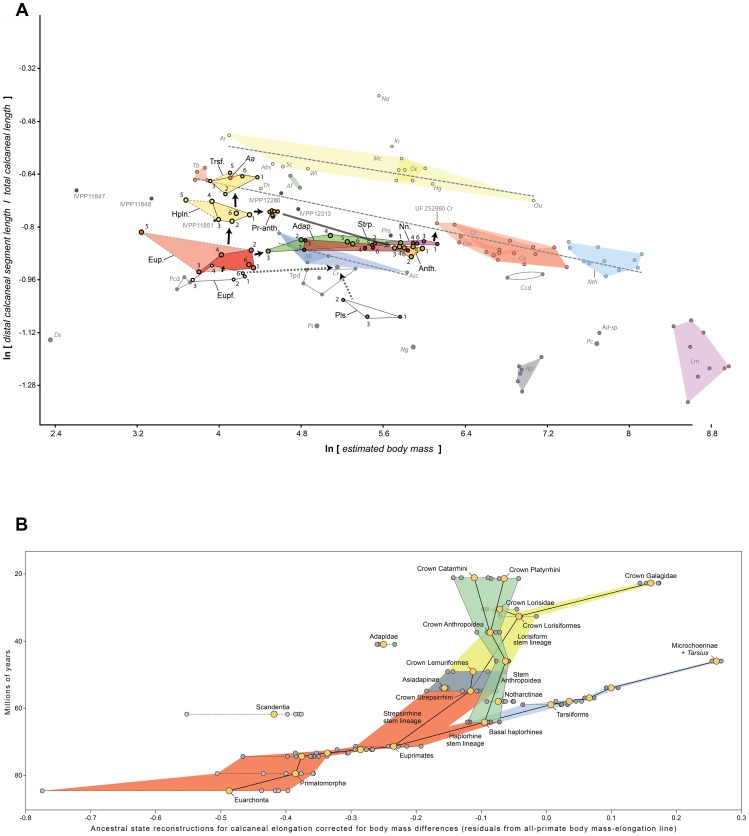
Plots of ancestral state reconstructions for nodes of interest in primate evolution. **A**, Nodal transitions imposed on fossil morphospace. We plot ancestral state reconstructions (ASR) of body mass and Calcaneal Elongation index on the morphospace of real taxa to visualize the PGLS-inferred pattern of calcanaeal evolution in the transition from stem- to Euprimates. Colored polygons with numbered points represent ancestral reconstructions for a given clade among different trees (i.e., different numbers indicate different trees – see Tables S2–S7 in File S1; Fig. 8). Note that there is slight overlap in the polygons representing the realm of euprimateform ASRs and Euprimate ASRs. The trajectory of change from the plesiadapoid ASRs to *Carpolestes simpsoni* is important for this analysis: it corroborates the idea that increases in grasping capacity should be linked to increases in calcaneal elongation, as *C. simpsoni* differs from other plesiadapids in having more proficient grasping capabilities and greater calcaneal elongation, but no evidence of greater leaping proclivities, otherwise [15]. Alternatively, if *C. simpsoni* is reconstructed as the sister taxon of euprimates (6), its position in the phylogeny is consistent with a basal trend of gradually increasing elongation relative to body mass. Regardless of tree used, the euprimate ancestor has lower elongation for its mass than any sampled taxon. This suggests parallel increases in early haplorhines and strepsirrhines coincidentally moved *Teilhardina* and *Cantius* onto the same regression line as defined by all euprimates. Non-allometric changes evolved through elongation at relatively constant body mass in haplorhines, and through increases in body mass, with only slight increases in elongation among strepsirrhines. **B**, Elongation residuals for ASRs relative to the “all euprimate” regression line (y = −0.068×+−0.39). Note this shows that despite different evolutionary trajectories of body mass and elongation change in early strepsirrhines and early haplorhines, both show similar changes in residual elongation relative to the “euprimate node.” Abbreviations: *Aa*, *Archicebus achilles*; Adap, Adapiform/ancestral strepsirrhine nodes; Anth, Anthropoid nodes; Eup, Euprimate nodes; Eupf, Euprimateform nodes; Hpln, Ancestral Haplorhine nodes; Pcd, Ptilocercidae; Tpd, Tupaiidae; Trsf, Tarsiiform nodes; Ccd, Cynocephalidae; Pr-anth, Protoanthropoid (including eosimiids) nodes; Nn, Notharctine nodes; Prs, *Proteopithecus sylviae*; see previous figures for other abbreviations.

The program *BayesTraits* allows estimation of ancestral states at all nodes of a tree of interest in which tip-taxon values are provided. We reconstructed body mass and elongation index at a number of nodes of interest on six different trees (Table 8, 9, 10, 11, 12, 13, Figs. 8, 9): 1) a maximum parsimony supertree that included all taxa of interest as described in the methods; 2) same topology as the first tree, but with basal divergences among euprimates assigned using fossils, rather than molecular calibrations; 3) maximum parsimony supertree with an additional constraint that adapiforms must be more closely related to haplorhines than to crown strepsirrhines (cf. Franzen et al. [59]); 4) same topology as the first tree with an additional constraint that *Tarsius* and anthropoids must share a common ancestor to the exclusion of omomyiforms (cf. Kay et al. [61]); 5) maximum parsimony supertree that uses the topology of Beard [69] for plesiadapiforms, linking them to dermopterans; 6) maximum parsimony tree based on the topology of Bloch and Boyer [15] for plesiadapiforms (treating *Carpolestes simpsoni*–the only carpolestid for which ankle morphology is known–as the euprimate sister taxon to the exclusion of other plesiadapiforms). Different models of evolution (i.e. Brownian motion with and without a directional trend) were assessed for each data set (body mass and elongation) on each tree. A directional model of trait evolution provided a better fit for the body mass data on all trees (as has been shown in other studies [57]). Calcaneal elongation was always best modeled by pure Brownian motion (Table 8).

**Table 8:** Tests of different models of character evolution for body mass estimates and calcaneal elongation on six alternative phylogenetic trees relating taxa of this study.

	No scalingNon-directional model	No scalingDirectionalmodel	Scaling parameter δNon-directional model	Scaling parameter δDirectionalmodel	Scaling parameter κNon-directional model	Scaling parameter κDirectional model	Scaling parameter λNon-directional model	Scaling parameter λDirectional model
Main tree, long branches, body mass	−162.3558	−157.9608	−159.4243	−**154.9677**	−161.2128	−155.2167	−163.2103	−161.2649
Main tree, long branches, elongation	**59.8914**	59.4145	59.3785	58.6943	60.1102	59.5431	59.1598	58.7270
Main tree, short branches, body mass	−164.1771	−160.5155	−157.4440	−**154.2960**	−162.3526	−156.4567	−165.1570	−161.4510
Main tree, short branches, elongation	55.9726	55.9723	57.2793	56.6791	**58.3737**	57.8692	54.9549	54.4739
Adapiform constraint, long branches, body mass	−165.1519	−160.2765	−161.6513	−156.8816	−162.4415	−**155.4430**	−166.0131	−160.9672
Adapiform constraint, long branches, elongation	**59.2397**	58.7246	58.7197	58.0267	59.1932	58.6244	58.4904	58.0014
No plesiadapiforms, long branches, body mass	−149.3272	−144.9656	−148.0982	−143.9740	−148.2682	−**142.9495**	−150.0456	−145.5001
No plesiadapiforms, long branches, elongation	**60.3872**	60.5442	60.5891	60.3830	60.3018	60.0460	60.0089	59.8953
Strict tarsier-anthropoid constraint, long branches, body mass	−161.7681	−157.0928	−159.4681	−154.7963	−160.548	−**153.8546**	−162.5497	−157.7366
Strict tarsier-anthropoid constraint, long branches, elongation	**55.9958**	55.5018	55.6492	54.9234	56.1360	56.1889	55.4388	54.9655
Plesiadapiform-dermopteran constraint, long branches, body mass	−166.1092	−162.5003	−161.3623	−**157.3264**	−163.8621	−157.7558	−167.0480	−163.3655
Plesiadapiform-dermopteran constraint, long branches, elongation	**62.5738**	62.1101	62.0914	61.4478	62.0748	61.8791	61.9341	61.4980
*Carpolestes*-primate constraint, long branches, body mass	−161.7932	−157.3033	−159.4157	−154.8462	−160.6837	−**154.8183**	−162.5991	−157.9402
*Carpolestes*-primate constraint, long branches, elongation	**62.2595**	61.7917	61.8985	61.2747	62.1583	61.6146	61.6444	61.2113

Generally speaking, resulting ASRs for most nodes of a given tree had overlapping 95% HPD levels (Tables S2–S7 in File S1). Problems with “over-conservativeness” of confidence limits on ASRs have been discussed in the past [93]–[95]. Therefore comparing mean estimates of the same node among different trees provides a different type of confidence assessment. It reveals the effect on the nodal reconstructions given uncertainty/error in the tree topology and branch lengths. While we reconstructed many nodes (Tables S2–S7 in File S1) we were principally interested in those reflecting the origin and early diversification of euprimates [euprimateforms, euprimates, crown haplorhines, tarsiiforms (omomyiforms and *Tarsius*), crown anthropoids, crown strepsirrhines, basal adapiforms/strepsirrhines, and notharctines]. Plotting ancestral reconstructions of body mass with those of calcaneal elongation along with extant values (Fig. 9A) shows the region occupied by estimates for the “euprimateform node” to be slightly below (lower average body mass reconstruction) but overlapping with the region occupied by estimates for the “euprimate node.” The combination of mass and calcaneal elongation values for all estimates of both nodes are well below the scaling relationship defined by early Eocene asiadapines, and instead are matched most closely by *Ptilocercus lowii*, with all known extant and fossil euprimates of the relevant size range having greater calcaneal elongation. The basal haplorhine node (defined here in all cases as the clade including *Tarsius*, anthropoids and all omomyiforms) occupies a region distinct from any other node reconstructed, being distinguished from the euprimate node region in having higher estimated calcaneal elongation values. The combination of mass and calcaneal elongation values is most closely matched in this case by the eosimiid calcaneus IVPP 11851. The region occupied by estimates for the tarsiiform node, which is defined as representing the common ancestor of *Teilhardina* and other omomyiforms (also including *Tarsius* in all cases except Tree 4), is also distinct from regions occupied by other nodes. It is distinguished from the haplorhine region by estimates with higher calcaneal elongation. The combination of mass and calcaneal elongation values is matched most closely by *Teilhardina belgica*, *Tetonius* cf. *homunculus*, and newly described [38] *Archicebus achilles* among sampled taxa (Fig. 9A). These reconstructions essentially lie along the overall euprimate regression line. The region of the crown anthropoid nodal estimates is well separated from those discussed so far by having much larger body mass. The region occupied by the notharctine nodal reconstructions is similar to that for anthropoids in body mass, but distinct in greater calcaneal elongation. The basal adapiform, basal strepsirrhine, and crown strepsirrhine nodal reconstructions occupy a region distinct from those for euprimateforms, euprimates, haplorhines and tarsiiforms, but overlap with those of notharctines and crown anthropoids. The overlap is due to relatively high body mass estimates for basal adapiforms/basal strepsirrhines and crown strepsirrhines in tree 1. In the combination of body mass and calcaneal elongation values, the notharctine and anthropoid nodes are closest to the primitive Fayum anthropoid *Proteopithecus sylviae* among sampled fossils. Not including the tree 1 reconstructions, the nodal estimates for basal adapiforms, basal strepsirrhines, and crown strepsirrhines are overlapping with values for the asiadapine *Marcgodinotius indicus.*

A plot of residual calcaneal elongation indices based on the nodal reconstructions for body mass and elongation (Figure 9B) suggests a pronounced shift towards increasing relative elongation over time incrementally from the euarchontan through euprimate nodes. These trends continue in parallel on both strepsirrhine and haplorhine sides of the euprimate cladogram. After the divergence of anthropoids and tarsiiforms, the tarsiiform lineage shows a continued increase in residual calcaneal elongation, while the anthropoid lineage stops increasing. The galagid lineage shows a rate of increasing residual calcaneal elongation similar to tarsiiforms, while the lorisid lineage predictably shows a reversal towards lower calcaneal elongation.

## Discussion

Our results suggest that calcaneal proportions are strongly influenced by body mass in primates. This allometric effect is tractable in being linear, with no apparent asymptotes, breaks in slope, or reversals in slope over the range observed. The relationship is apparent in both prosimians and anthropoids, despite the fact that many anthropoids are not “tarsifulcrumators” [29]. The PGLS coefficients of this linear relationship change little when using different taxonomic subsamples representing either closely related species or monophyletic clades (Table 5: however, see further discussion below). Fleagle [96] has described scaling relationships that behave this way as potentially explainable by the need to maintain “functional equivalence” whereby changes in shape maintain proportionality between mechanically relevant variables. In this case, those variables are likely the moment of the plantar flexors and body mass. In Fleagle’s words allometric relationships can be interpreted as lines of “functional equivalence” in “cases in which… various groups show a similar slope” and/or in cases where “[slope] meets theoretical expectations from some mechanical mode” (p.8). Both of these criteria are met by the results of our analyses. Demes and Günther [31] also believed that maintainence of functional equivalence can be expected to drive covariation between certain variables and body mass. In such cases they implied that these relationships could be treated as lines of subtraction: “variation around those lines reflects the influence of different ecological niches *and* selective strategies” (p.139).

Despite the observation that the relationship between body size and calcaneal elongation changes little under certain permutations of sample composition, we acknowledge that the recovered “rule” of negative allometry *is* nevertheless contingent on the composition of the sample, in the sense that one could easily pick out a sample of primates of different body size with identical calcaneal elongation values. Furthermore, certain subsamples of our analyses (e.g., Eocene Primates: Table 5) show a substantially different slope and intercept than what we consider to be the “fundamental allometry” of the clade. However, our point is that when sampling either a) the very earliest primates (within the first million years of the beginning of their fossil record), b) closely related primates (e.g., all species within a genus or extant family), or c) as exhaustively as possible, the result is negative allometry with a very similar slope in all cases. The fact that sampling only Eocene euprimates results in a very different slope may be explained by insufficient body size range within certain clades and/or insufficient overlap in behavior among clades.

An additional regression on proximal and distal calcaneal segments indicates that the change in calcaneal elongation index with body mass is effected by increasing the proximal segment to a greater degree than expected for isometry, and increasing the distal segment by a lesser degree (Table 6) with increasing body mass. Furthermore, results of phylogenetic ANOVA on elongation residuals from this regression (Table 7) suggest that at any given body size, different locomotor repertoires are associated with different degrees of calcaneal elongation in prosimian primates, but not in anthropoids. It is also clear that patterns of calcaneal elongation are clade specific, with strong phylogenetic co-variation in distal calcaneal length and the calcaneal elongation index used in this study. As such, for a given taxon, the calcaneal elongation values of its close relatives better predicts its elongation than knowledge of its behavioral category. Therefore, estimating behavior from fossil data using size-standardized elongation must be done in the context of its close relatives, if at all.

This “phylogenetic signal” in calcaneal elongation is consistent with the finding of Moyà-Solà et al. [7] that as a group, primates exhibit greater calcaneal elongation than non-primates. As has been noted previously [12], among *primates as a whole* there is not a consistent association between degree of calcaneal elongation and leaping, specifically because anthropoids fail to demonstrate this relationship. However, especially when inferring functional/adaptive significance of morphological variation during euprimate origins, it is critical to recognize additionally that: 1) calcaneal elongation *does* correlate with leaping proclivity (or at least locomotor agility) among prosimians, 2) the ancestral euprimate likely had lower elongation than any similarly-sized extant euprimates and 3) there are separate parallel trends of increasing calcaneal elongation in haplorhine and strepsirrhine descendent lineages from the ancestral modern primate.

### Fundamental Allometry of Primate Calcaneal Ratios

Running separate PGLS regressions for extant and fossil taxa results in similar slopes (−0.06 to −0.08) that may represent something approaching the “fundamental allometry” of calcaneal shape change when other factors (behavioral shifts/modifications and random morphological drift) are taken into account. Clearly, this indicates that body mass can directly influence calcaneal elongation and should be considered when comparing this morphology among primates to infer aspects of locomotion.

That calcaneal elongation and body mass are inversely correlated is perhaps not surprising if one considers that available muscle force is expected to scale to the 2/3 power of mass [97], with large animals having relatively less available muscle force in proportion to mass (Fig. 10). Less calcaneal elongation allows for more effective mechanical advantage of the Achilles tendon, soleus muscle, and gastrocnemius (triceps surae). How much does this allometry mitigate the divergence between mass-to-be-moved and available muscle force with increasing body size? We can model the system and provide an estimate: If we assume euprimate locomotor modes evolved in a 10 gram(g) primate (as suggested by [98]), then using the average estimate for the “all primate regression” (Table 4), the distal calcaneal segment (load arm) is predicted to be 58% of the total, leaving 42% for the heel (lever arm). If we assume a calcaneal length of 1 unit total, then the moment of the distal segment is 0.58*(10 g) and the plantar flexors must contribute 0.42*(13.8 g) of force to simply resist body weight when both the load- and lever-arms are horizontal (we model this situation statically for sake of simplicity). Assuming muscle mass and physiological cross-sectional area scale isometrically with body mass, then the force that muscles can produce scales to the 2/3 power of the body mass: for instance, in a 100 g animal, the plantar flexor muscles would only be able to produce 64 g of force with the same effort. However, if this animal has calcaneal elongation percentages similar to that of an animal with a mass of 10 g, then the effort required to move the load arm will have increased by 115% (i.e. it will be 138 g). On the other hand, if the allometrically expected decrease in calcaneal elongation is applied to the 100 g animal, the increase in effort is only 53% relative to the available muscle area. To look at it another way, without the allometric trend documented here, the effort multiplication experienced by a 7 kg animal, relative to a 10 g one (3.8 times) would be experienced already in a 550 g animal (Fig. 10). Therefore the observed allometric effect delays major increases in muscle effort by an order of magnitude in body size. The effect of allometry on the mechanics of primate leaping is also affected by the size of euprimates when they first appeared (at least in terms of effort multiplication from a “starting point”). We used 10 g initially, but 75 g or 100 g appear more likely (from the ancestral state reconstruction analyses – see above). At these masses, the entire extant prosimian body size range can be covered while increasing muscle recruitment effort by a factor of 2.5–2.6, instead of factors of 4.1–4.6, respectively.

This exercise also allows us to assess the evolutionary conditions under which a euprimate lineage might most easily deviate from what is predicted from allometric effects. If relative effort is multiplied as a consequence of body mass increases, it follows that it will be diminished by body size decreases. Therefore, in a lineage evolving to smaller body size, calcaneal elongation can be increased faster than dictated by allometry such that, relative effort does not decrease, but instead stays the same compared to the muscle effort experienced by the ancestor. We can model this in a way similar to what we did for body size increases: specifically we can ask the question: “as body size decreases from the ancestral state, what is the maximum increase in calcaneal elongation that will keep the relative muscle effort constant?” Again, the answer depends on the starting size of the ancestor. So for a 10 g primate, with proportions dictated by the “Eocene primate line” (y = −0.068× –0.386), evolving smaller (which seems unlikely), to 5 grams, allows a ∼4% increase in the length of the distal calcaneal segment (relative to what would be dictated by the allometric relationship) without increasing the relative muscle effort required. However, reduction in mass from 75 g to 5 g in a lineage would permit a 17% increase in calcaneal elongation (relative to the allometric prediction). This equates to elongation similar to that observed in extant *Galago senegalensis*, but in a much smaller animal (1/50^th^ the mass). If we imagine that this 5 g species then began evolving to larger body mass again, and following the proportional change expected given our allometric slope of 0.06–0.08, by the time it reached the size of modern tarsiers (100–120 g), it would have elongation in the range of omomyines. Since the slope of the allometric line is not one of perfect functional equivalency, presumably other anatomical/behavioral changes will accompany decreasing elongation as body size increases, allowing the animal to meet the demands of the environment for its locomotion. If some lineage descended from these hypothetical tarsier-sized primates became smaller again, more elongation could result without increasing effort. In this “ladders and chutes model” of changing calcaneal elongation, extreme levels of elongation in galagos and tarsiers could happen “least expensively” through several within-lineage trends of decreasing and then increasing body mass.

While this model is simplified for the discussion here and by definition hypothetical in the face of the actual evolutionary history of primates, it still provides some potentially useful insights that can be applied to interpreting the morphology of extant and fossil primates. For instance, the allometric effect of body size has a much more significant impact in constraining morphology if the ancestral primate was in the realm of 10 g than if it were closer to 200 g. Additionally, if the ancestral primate was 10 g, with proportions similar to what are observed for eosimiids estimated at that body size, then it is unlikely that reduction of body mass in these taxa could produce the shifts in elongation that we see in various modern clades. Either subsequent evolution to larger body sizes would have had to occur first, with reduction in size to follow (allowing energetic-cost-diminished elongation), *or* increases in elongation without body size decreases must have occurred along with other modifications to behavior that balanced out the increased muscular effort required by the decreases in mechanical advantage. Most likely both of these scenarios have operated in different lineages to allow for non-allometric increases in calcaneal elongation during the history of primate evolution.

### Behavioral and Evolutionary Significance of Changes in Calcaneal Elongation

#### Differences among primates with different degrees of leaping proclivity

**Figure 11:**
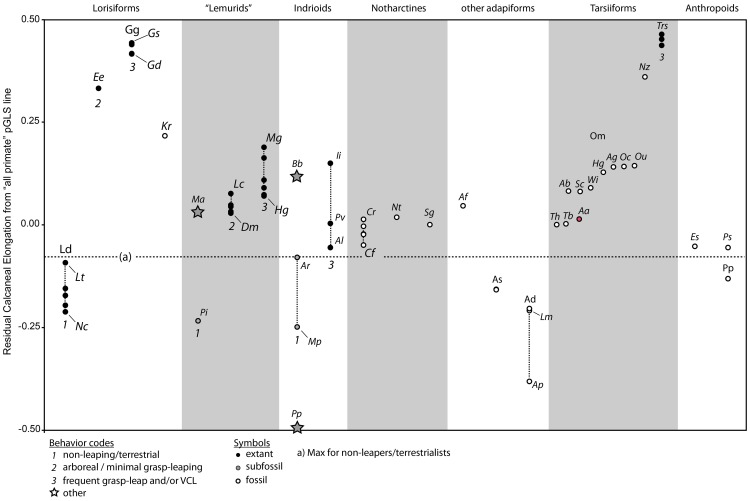
Box plots of residual elongation. We plot species mean values for residual elongation from the all primate line (Residual A from Table 1). The distribution of values within clades corresponds very well to degree of agility of locomotion. For fossils the variation corresponds with locomotor agility hypotheses based on additional skeletal features [30]. When these residual data sets are examined with phylogenetic ANOVA, a strong relationship between elongation and behavior is revealed (Table 7) meaning that calcaneal elongation is broadly related to behavior in contrast to the conclusion of Moyà-Solà et al. [7]. See previous figures for taxon abbreviations.

Prior to this study the question of whether and how distal elongation of the ankle reflects leaping proclivity had been answered in very general terms using an impressively comprehensive sample [7]. Results from our analyses suggest a functional association between presence/degree of leaping specialization and calcaneal elongation, at least among prosimian primates. The signal from our phylogenetic ANOVA of elongation residuals, while weak at the scale of all prosimians, suggests significant differences among leapers (greatest calcaneal elongation), arboreal quadrupeds (intermediate calcaneal elongation) and slow climbers/terrestrialists (low calcaneal elongation). We suggest that this signal appears weaker than it should due to 1) limited knowledge of the phylogenetic history of prosimians, 2) imperfect behavioral categories and 3) the limits on ecological diversity across prosimians primates, that if it were greater, would add statistical power to our analyses. Within clades striking correlations between calcaneal elongation residuals and behavior are apparent. For instance, calcaneal elongation residuals in cheirogaleids is strongly correlated with differences in leaping proclivity among taxa described by Gebo [74], with *Cheirogaleus major* leaping the least (6% of locomotor bouts) and having the lowest residual (0.032), *Cheirogaleus medius* leaping more (21%) and having a higher residual (0.048), *Mirza coquereli* leaping more yet (27%) and having a higher residual yet (0.162) and *Microcebus murinus* leaping most (38%) and having the highest residual (0.182). The relationship clearly applies across lorisiforms (Fig. 11). However, even on the smaller scale of galagos included in Gebo’s [74] study, there is some variation that matches previously identified behavioral differences: *Galago senegalensis* leaps more than any other galago (63%) and has the highest residual (0.442). However, variation in leaping reliance among other galagos, as documented by Gebo [74], is not correlated with calcaneal elongation residuals (this is not including *Euoticus*, whose lower residual yet is predicted by its use of large diameter supports and claw-clinging [76], see Fig. 11). Among larger-bodied taxa the relationships generally hold: within non-cheirogaleid, non-indriid lemuriforms, *Daubentonia* is the least specialized for leaping [75] and has the lowest residual value (0.028), while *Varecia* is slightly more acrobatic according to Gebo [74] (leaps were 21–25% of the locomotor bouts) and its residual is higher (0.048). *Lemur catta* leaps more frequently when on the ground (55%) than *Eulemur* (44%), but less while in the trees (22% v. 30–37%). Nevertheless *Lemur* and *Eulemur* are both more leaping reliant overall than *Varecia* and have accordingly higher residuals (0.072–0.076). *Lepilemur* is a more committed leaper (no data in Gebo [74] though) and has a higher residual yet (0.106). Another group for which the relationship appears to break down slightly is *Hapalemur* that leaps more (56% – although it should be noted that this value is close to that for *Lemur catta* on the ground), but has residuals similar to those of other lemurids that leap less (0.070–0.072). Interestingly, Hamrick [99] has noted that *Hapalemur* also lacks predicted adaptations for vertical clinging in its wrist. Therefore some unappreciated aspect of *Hapalemur*’s ecology or evolutionary history may have muted the development of adaptations to vertical clinging and leaping. Overall the correlation is striking when considering leaping behavior on a finer scale than done in our phylogenetic ANOVA and looking within groups.

Going back to the exceptions (some galagos and *Hapalemur*), an important point to consider here is that “average leaping reliance,” as documented by Gebo [73] as a percentage of all locomotor bouts observed, is neither a sufficient or necessary condition for the hypothesis that an animal will exhibit adaptations for leaping. Many assumptions underlie the expectation for such a correlation. Another behavioral variable that may conceivably correlate with morphology as well or better than documented leaping reliance is “leaping performance.” For example, two species of the same size may use leaping with similar frequency, but one may consistently leap farther than the other, or, when stressed, have a maximum leaping distance that is significantly farther. It should be noted that such definitions of performance are not necessarily expected to correlate with energetic efficiency in a behavior so coarsely defined as “leaping.” However, if one were to specify “leaping” more narrowly in terms of substrates used and distances covered, measurements of “energetic efficiency” might be more closely equivalent to “leaping ability” in terms of achievable leaping distances. Studies that evaluate leaping performance in a sample broad enough to be relevant here have not yet been done. Finally, “leaping reliance” may be defined not only as frequency during daily routines, but also by its recruitment for locomotion fulfilling particular, critical roles. That is, a small percentage of total daily locomotion may serve in predator evasion, or alternatively, in ambushing prey. However, if a species has been observed to use leaping during most predator evasion or predation events, one could reasonably hypothesize that these events put a strong selection pressure on high leaping performance, even if leaping is a small part of the daily routine.

Previous studies have led to the conclusion that after a certain size threshold, there is no longer a benefit bestowed by ankle elongation to large leapers [31]–[33]. Some of the discussion above notes patterns in our data on lemurids that suggests against this. Furthermore, several additional lines of evidence show that while allometric constraints reduce the degree of elongation in large taxa, offsets between behavioral categories still exist. For example, while Gebo and Dagosto [33] concluded that the indriid foot was primarily adapted for climbing, not leaping, we note that the calcaneus of *Indri indri* is a strong positive outlier to lemuriform regressions as well as “all primate” regressions. This is true whether one considers the calcaneal ratio, or the absolute length of the distal calcaneal segment relative to body mass (Table 1, Res A–B). This suggests a leaping effect on residual calcaneal elongation in *Indri*. On the other hand the position of other indriids contradicts the general trend (they are committed leapers, but have low residuals) and has been difficult to explain [33]. However, it is well-documented by the sub-fossil record that known indroid outgroups to the extant Indriidae were not leapers [100]. Furthermore, it is even possible that more than one clade within crown Indriidae has adapted to leaping independently, and that different clades have increased leaping specialization at different times (i.e., some more recently than others). A debated hypothesis that, if true, would add plausibility to this idea is that *Mesopropithecus*, judged to be anti-pronograde due to an intermembral index greater than 100 [100], is the sister taxon to extant *Propithecus* [101] (and probably *Avahi* as well). If an independent behavioral transition to leaping happened very recently in *Avahi* and *Propithecus*, compared to *Indri* [66], then some aspects of the skeleton may still be “adapting” in those taxa. Strong phylogenetic co-variance in calcaneal elongation residuals demonstrated by our analyses (Table 7, Fig. 11) in fact implies that this is a reasonable expectation.

Another more recently posited hypothesis based on molecular data places the Paleopropithecidae as a sister group of indriids, and places archaeolemurids as sister of these two clades [66]. Regardless of when or how many times leaping evolved in extant indriids, this suggests a long evolutionary history of non-leaping, slow-climbing, and/or terrestrialism in the indrioid clade. If the ancestral indriid was a slow climber and/or terrestrial and had calcanei with extremely low elongation ratios [certainly a possibility given that such a condition exists in certain subfossil species (Table 2)], then all extant indriids may indeed have experienced increases in elongation that cannot be explained by allometry and reflect increased leaping compared to their ancestors. While such changes can only be appreciated with analyses that extensively sample subfossil lemur morphology, the limited subfossil data in our study show that residual elongation in extant indriids is greater than that in *Archaeolemur*, *Paleopropithecus*, and *Mesopithecus*, which are reconstructed as semi-terrestrial, antipronograde, and slow-climbing, respectively [100] (see Fig. 11). While *Babakotia* actually has high residual calcaneal elongation, as stated in the methods, we do not have specific predictions for the elongation constraints of inverted quadrupeds, and/or highly specialized, sloth-like quadrumanous suspensory taxa. It may be that quadrumanous suspension allows and/or selects for greater elongation than is possible/useful for pronograde and orthograde animals of similar size in some situations. The fact that *Cynocephalus volans* has the greatest degree of elongation among non-primate euarchontans, despite also being the most massive in this group, may reflect a similar functional correlation. Comparison of elongation in sloths to that of other xenarthrans could provide data to test this idea. On the other hand, *Babakotia* and *Paleopropithecus* have the lowest residual distal calcaneal segment lengths of any sampled euprimate (see Table 1, Res. B: −0.726 and −0.634, respectively). The only other primates with similarly low residuals are the hylobatids (Table 1). *Avahi* (−0.109), *Propithecus* (−0.008), and *Indri* (0.156) are all much higher.

Our explanation for the muted pattern of distal calcaneal elongation among indriid leapers as a consequence of recent and potentially multiple transitions to leaping from non-leaping indrioid ancestors, if correct, is most likely still only part of the story. This muted pattern is plausibly also contingent on, or driven by, 1) indriid leaping specializations first evolving in an ancestor of a larger size than the ancestral galagos and 2) the lack of evidence for any pronounced lineal decreases in body mass among indrioids [the evolutionary situation in which our model (above) suggests that increases in tarsal elongation can be most profound]. Our ASRs suggest that the ancestral galagid was around 250 g, while the nodes of the indrioid clade are reconstructed as having been between ∼1,500–2,000 g (Tables S2–S7 in File S1) with little variation and no obvious trends. These data begin to reconcile ideas about body size limits for “ankle powered leaping” with apparent paradoxes such as different structural solutions for leaping employed by taxa of similar body mass (i.e., *Avahi* and *Otolemur*). While our study suggests there is no strict body size “cut off” for a tarsal-lengthening effect from leaping specialization, a *strong* tarsal-elongation response to frequent leaping selection would appear to be most likely in small-bodied lineages rather than large ones given the constraints of the observed allometric line and the finding that (according to our model) tarsal elongation can happen most easily during lineal decreases in body mass.

#### Ancestral state reconstructions

Among available non-euprimate eurchontans no clear allometric trend is present (Table 2). Taxa exhibiting values for calcaneal elongation that are on the low end of euprimates (for their body masses) are the plesiadapoid plesiadapiform *Carpolestes simpsoni*, tupaiid tree shrews, and the dermopteran *Cynocephalus volans*. Looking at the nodal trend leading from the base of Euarchonta to Euprimates shows predominantly body size increases and minimal elongation increases (Tables S2–S7 in File S1). While all reconstructions of the ancestral plesiadapoid have significantly larger body size and lower elongation than *C. simpsoni*, we note that poor taxon sampling of more primitive species may be driving this pattern. If more primitive, much smaller (and much older) carpolestids such as *Elphidotarsius florencae*, and more basal, small plesiadapoids such as *Chronolestes simul* could have been sampled, the ASR for plesiadapoid body mass would likely have been much smaller. Likewise if one assumes that the ankle morphology of *C. simpsoni* is similar to those of both *E. florencae* (a distinct possibility) and the most primitive plesiadapoids, then the overall trend in plesiadapoid evolution leading to *C. simpsoni* would be reconstructed as paralleling that leading to the euprimate ancestor more than can be inferred from our results (Fig. 9A: note right-most dashed arrow). This possibility can only be directly addressed through new fossil discoveries.

Regardless of the accuracy of the plesiadapoid ASR in our analysis, *C. simpsoni* has a higher elongation residual than any estimate for the euprimateform node or any nodes more basal, suggesting corresponding functional differences in *C. simpsoni*. The hindlimb skeleton of *C. simpsoni* does not exhibit obvious potential correlates of leaping [15]. Instead, the most striking aspects of *C. simpsoni*, which distinguish it from other plesiadapiforms, are its euprimate-like divergent and seemingly opposable hallux, and short non-hallucal metatarsals [15]. The grasping hallux and tarsifulcrimating foot of both euprimates and *C. simpsoni* appears to have happened coincident with an increase in distal calcaneal elongation. These acquisitions may have happened in parallel or may be homologous [15]. The combination of features in *C. simpsoni* is consistent with Moyà-Solà et al.’s [7] hypothesis that a moderate amount of calcaneal elongation in euprimates is a function of development of a specialized hallux and tarsifulcrumating foot, not leaping. More specifically, by analogy with *C. simpsoni* (which shows no other obvious correlates of leaping), we can explain increasing elongation as a result of compensation for evolution of tarsifulcrumation alone in any primate lineage that does not exceed the elongation residual values seen in *C. simpsoni*.

After evolution of the lineage representing the ancestral stock of crown primates (represented by the ASR for the Euprimate node in our analyses: see Fig. 9A), subsequent further elongation (beyond that seen in *C. simpsoni*) is reconstructed as having occurred along branches leading to the ancestral strepsirrhine and haplorhine lineages (Fig. 9A, B). This further elongation therefore exceeds the amount explainable by acquisition of a tarsifulcrumating foot.

Other authors have suggested that the didelphid *Caluromys* might be the best available analogue for early euprimates [10], [11], [98]. A cursory look at didelphids does not provide any support for Moyà-Solà et al.’s [7] hypothesis. Despite increased specialization of the hallux and greater prehensility compared to some of its relatives, the arboreal marsupial *Caluromys philander* does not exhibit increased actual or a higher residual calcaneal elongation [n = 3, ln(DL/TL) = −1.40±0.05; ln(CW*CD) = 1.53±0.15 (body mass estimate using equation derived from primates = 161 g); residual from all primate allometric line = −0.68±0.04] compared to its more generalized scansorial relative *Monodelphis brevicaudata* [n = 3, ln(DL/TL) = −1.30±0.06; ln(CW*CD) = 0.56±0.08 (body mass estimate using equation derived from primates = 45 g); residual from all primate allometric line = −0.62±0.07]. At the very least, this suggests phylogenetic dependency on whether the hallucal grasp complex is functionally correlated with the distal calcaneal segment length.

If *C. simpsoni* is reconstructed as the sister taxon of euprimates to the exclusion of other plesiadapoids (Table S7 in File S1; Fig. 9A, note left-most dashed arrow), its position in the phylogeny makes it a contributer to the basal trend of gradually increasing elongation relative to body mass – which could relate to selection for both/either improved grasping and/or leaping early in primate evolution. Undoubtedly the large differences in the reconstructed evolutionary history of body size change implied for the lineage leading to *C. simpsoni* is greatly influenced by missing data on small-bodied early plesiadapoids such as *Chronolestes simul*, *Pronothodectes matthewi,* and *Elphidotarsius florencae*.

The trajectories in both haplorhine and strepsirrhine lineages suggest significant functional/behavioral shifts associated with increasing elongation, because these increases do not follow the allometric slope identified earlier in this study. Haplorhines evolved mainly by increasing elongation at the same size as the ancestral euprimate, while strepsirrhines evolved mainly by increasing in body size with only slight increases in elongation compared to the ancestral euprimate. Nonetheless, improved leaping in both clades is suggested by the fact that they both approach, rather than parallel, the “all euprimates” regression line (thereby acquiring greater “body-size standardized” elongation than hypothetical taxa represented by more basal nodes). This pattern is also clear on a plot of residual elongation against node depth (Fig. 9B). The evidence for parallel evolution of elongated tarsals is consistent with the long known fact that omomyiforms have increased their foot length by significantly lengthening bones of the foot beyond the transverse tarsal joint (cuneiforms and cuboid) possibly beyond the degree exhibited by extant cheirogaleids in many cases [30].

It is important to note that the ancestral state reconstructions here suggest that calcaneal elongation as seen in the early fossils *Teilhardina*, *Anchomomys* or *Cantius*, or leaping proficiency as seen in even “generalized” modern strepsirrhines, was not a synapomorphy of Euprimates. This is especially relevant given uncertainties about the functional significance of nails compared to claws and the observation that anatomical details of distal phalanges exhibited by early omomyiforms [52] differ markedly from those of early adapiforms [102]. If nails are particularly relevant in improving leaping performance then we might even expect that non-hallucal nails evolved in parallel with improved leaping in two major clades of euprimates (possibly from a common ancestor having a more “*Carpolestes*-like” foot). A leaping adaptation for nails remains plausible since specialized hallucal grasping alone does not explain the loss of claws (as specialized graspers *Caluromys*, *Petaurus*, and many other marsupials retain large non-hallucal claws, while also sporting a large, divergent opposable hallux with a nail). Furthermore, the idea that nails evolved to aid grasping in large-bodied arborealists [103] cannot be entertained given the presence of nails in 30 g *Teilhardina* and the lack of fossil evidence for more basal euprimates having been any larger than this.

Another implication of the ancestral state reconstructions is that the evolution of notharctines is not explained by decreased elongation due to increasing body size from an animal similar in size and ankle proportions to *Teilhardina*. In other words, the alignment of *Teilhardina* with notharctines along the “all euprimates” regression line would appear to be coincidental relative to the phylogenetic history of the two groups. This also means that it is difficult to talk about “behavioral equivalence” in these two taxa relative to the allometric line. This perspective, that *Teilhardina* and *Cantius* have achieved ankle elongation in parallel and cannot be equated or contrasted behaviorally, would be further supported if future discoveries of *Teilhardina* show the typical omomyiform pattern of cuneiform elongation. This raises the question of “for what clades does the allometric relationship explain reconstructed evolutionary change?” There are several. The evolution of adapines from an asiadapine-like ancestor could be explained by increases in body mass with allometrically expected decreases in elongation. Notharctine evolution starting with known *Cantius* is explained by increases in body size with allometric decreases in ankle length. Likewise, Omomyinae have followed an allometrically predicted decrease in ankle elongation from a smaller-bodied, more basal tarsiiform. Finally, the morphological change in anthropoid calcaneal proportions can be explained by the allometric expectation of decreasing ankle elongation from an eosimiid-like ancestral haplorhine.

### Behavioral Interpretation of Specific Early Euprimates

We were able to resolve and account for allometric effects on calcaneal elongation in this study, providing improved potential for interpreting the behavioral significance of residual calcaneal elongation. However, because of the strong phylogenetic co-variance of calcaneal elongation recovered in our analyses, reconstructing locomotor behavior from the calcaneus alone must take into account several lines of information. The presence of parallel trends of increasing elongation in basal haplorhines and strepsirrhines (i.e., which goes beyond what might be expected for improvements related to grasping alone [7]) suggests consistent presence of selection for improved leaping (given other results presented here suggesting an association between leaping proclivity and calcaneal elongation in extant prosimians). Selection for improved leaping implies that leaping must have constituted an important activity in the locomotor strategies of at least the earliest ancestors of both haplorhine and strepsirrhine clades. If we try to answer the question “how much did they leap and how effectively?” the only answer that is defensible is “enough that it improved their fitness if they did it well.” As discussed above, this might mean very infrequently relative to the daily activities of a given animal. Therefore, leaping frequency need not have increased, but leaping performance probably did. This again reveals a gap in the behavioral data needed to assess the functional significance of calcaneal elongation. Behavioral categories based on overall frequency of different behaviors [74] are not enough. What is really needed is a classification based on 1) performance in certain settings, and 2) frequency of use in specific settings where fitness gradients are likely to be high (e.g., predator escape, predation). Defining performance is clearly a difficult task as it requires artificial behavioral classifications and assumptions about the critical aspects of performance. Technological advances in lab and field methodologies should make future collection of such data increasingly feasible.

With all of these caveats in mind we now re-consider the behavioral significance of calcaneal elongation in various fossil primates when allometry and phylogenetic co-variance are accounted for.

#### Notharctines

Gebo [30], Rose and Walker [104], Gebo et al. [40], Fleagle and Anapol [105], Schmitt [106], Silcox et al. [107] and others have interpreted a similar range of locomotor behaviors for early North American notharctines. Most authors suggest that *Notharctus* and *Smilodectes* exhibit some degree of VCL leaping with increased leaping proclivities compared to *Cantius*, the most basal notharctine. Previous studies of the calcaneus added little to these interpretations. For instance, Gebo et al. [40] documented that *Notharctus* exhibited less calcaneal elongation than *Cantius*. While this is true, it appears to contradict the general conclusion about notharctine behavioral differences outside of an allometric context. As we have shown, most of the difference in calcaneal elongation among early North American notharctines can be explained by body size differences. However, if one examines the residual calcaneal elongation values (Table 1; Fig. 11), *Notharctus* actually exhibits a higher elongation residual (indicating more elongation for its body size) than all species of *Cantius*. *Smilodectes* exhibits less elongation than *Notharctus*, which actually is consistent with locomotor interpretation based on analyses of the humeral head [106]. Therefore an allometric treatment of calcaneal elongation returns a pattern broadly consistent with that from other regions of the skeleton and indicative of more leaping in *Notharctus* than in *Cantius* [30], [106], [107]. Comparing residual calcaneal elongation of these fossils with that of extant taxa, shows the fossils to exhibit less residual calcaneal elongation than most arboreal quadrupedal, leaping and vertical clinging and leaping primates. This likely indicates that leaping behaviors were not as effective in any early Eocene adapiforms. This is consistent with a recent analysis of body proportions by Gingerich [108], showing *Notharctus* to be most similar to *Cheirogaleus* and well-separated from leapers it has been compared to previously like *Lepilemur* and *Avahi*. Therefore, despite the possibility that differences between *Notharctus* and extant leapers are results of clade shifts in morphology that are not reflected by behavior, the fact that the rest of the skeleton lacks leaping specializations decreases the likelihood of this for us. By extension, *Cantius* and *Smilodectes* would also be considered ineffective or infrequent leapers. The inference for these latter taxa could be tested with analyses of more complete skeletal material.

#### Asiadapines

Rose et al. [37] argued that early Eocene (∼53–54 mya) adapiforms *Marcgodinotius* and *Asiadapis* from Gujarat, India, could be reconstructed as active arboreal quadrupeds with some leaping proclivities based on phenetic similarity to *Cantius*. For the calcaneus, taking a phenetic approach to a locomotor reconstruction is problematic given the results of this study. Given the small body size of asiadapines compared to *Cantius*, the similar levels of calcaneal elongation equate to very low residual calcanael elongation suggesting a slow-climbing lifestyle (Table 1; Figs. 4, 5, 6, 7, 11). What is unclear from our ancestral state reconstructions is whether asiadapines have reverted to smaller body size and shorter ankles from a larger-bodied, longer-tarsaled ancestor (as implied by ASRs based on the maximum parsimony supertree – Table S2 in File S1) or whether the common ancestor of asiadapines and its sister taxon had body-mass and elongation proportions more similar to asiadapines. If the former scenario is true, this definitely suggests a decreased emphasis on leaping in these taxa relative to the general strepsirrhine stem. If the latter is true, it is more difficult to reconstruct their locomotor repertoire relative to other primates. However, in either case, short ankles at such small body size would appear to suggest relatively ineffective leaping. Turning to other features of the asiadapine skeleton: although astragalar depth and a strong posterior trochlear shelf (present in asiadapines and *Cantius*) may demonstrate leaping, this has never been broadly demonstrated aside from contrasts between lorisids and other strepsirrhines. Furthermore, the femur does not demonstrate leaping proclivities beyond what might be expected in a mainly quadrupedal *Cheirogaleus*. One feature not mentioned by Rose et al. [37] which suggests against leaping is the very distal position of the third trochanter (it is positioned distal to the lesser trochanter). This morphology is reminiscent of other taxa which lack prosimian-like specializations for leaping such as sciurid rodents, plesiadapiforms [109], and basal stem catarrhines [110].

In sum, we think Rose et al.’s [37] interpretation of these taxa could be correct, depending upon how the morphology of asiadapines compares to the ancestors they share with other strepsirrhines. However, in the context of our analysis the available data seems to suggest that compared to notharctines including early *Cantius*, asiadapines were less specialized for and probably less effective at leaping.

#### Adapines

Various authors [30], [111] have interpreted *Adapis* and *Leptadapis* as cautious slow-climbers and possibly lorisid-like. Our results corroborate this perspective.

#### Anchomomys

Roig et al. [112] and Moyà-Solà et al. [7] made a different allometry-based argument for the lack of specialized leaping in *Anchomomys frontanenysis.* Their study was apparently motivated by the seemingly high degree of calcaneal elongation in this small-bodied adapiform. Their conclusion was thus somewhat surprising given the qualitative appearance of strong elongation in this bone. In our analyses, *Anchomomys* exhibits moderate residual calcaneal elongation compared with other early euprimates: it is higher than in any notharctines, *Teilhardina* and *Tetonius*, yet lower than in *Absarokius* and omomyines (Table 1, Fig. 11). This suggests to us that the *Anchomomys* lineage experienced selection for greater improvements in leaping compared to lineages of notharctines, *Teilhardina*, and *Tetonius*, though we would not argue that it was a specialized vertical clinger and leaper and are basically in agreement with Roig et al. [112] and Moyà-Solà et al. [7] on this point.

##### Omomyiforms

Based on a variety of postcranial elements Gebo [30] argued that omomyiforms were “cheirogaleid-like in their locomotor behavior.” He also argued that anaptomorphines (*Teilhardina* and *Tetonius*) relied on specialized leaping to a lesser degree than omomyines (*Arapahovius, Hemiacodon*, and *Washakius*) or microchoerines. Our results for the same taxa are completely consistent with this conclusion. The “anaptomorphines” have residual calcaneal elongation that is lower than and non-overlapping with that of omomyines in our analyses (Table 1, Fig. 11). However, our study included additional anaptomorphines (*Absarokius*) and omomyines (*Omomys*). *Absarokius* shows greater residual calcaneal elongation than *Shoshonius*, so the pattern is no longer as clean. Furthermore, recent phylogenetic analyses based on large data sets rarely recover “anaptomorphine” and “omomyine” clades as delimited above [54], [113], [114]. For instance *Arapahovius* is now usually reconstructed as phylogenetically closer to “anaptomorphines” than omomyines. When interpreting behavior from the calcaneus in omomyiforms an important additional consideration is the contribution of the distal tarsal elements to overall tarsal elongation, which is lacking in other euprimates with calcaneal elongation. Savage and Waters [115] attribute cuboid bones to both *Tetonius* and *Arapahovius*. That attributed to *Tetonius* has little more elongation than what is seen in much larger notharctids, while that of *Arapahovius* shows more elongation. This suggests that while the degree of distal calcaneal elongation in *Tetonius* has an effect on overall elongation of the tarsus, which is similar to that in other euprimates, in other omomyiforms calcaneal elongation is only part of the story. This therefore accentuates the implied differences in overall tarsus elongation suggested by differences in residual distal calcaneal elongation observed in *Tetonius* as compared to *Arapahovius* (Fig. 11). Nevertheless, this additional information on the cuboids is consistent with the information from the calcanei. Therefore it is valid to state that *Teilhardina* and *Tetonius* (“anaptomorphines”) show equivalent distal calcaneal elongation to notharctines that suggests a similar, mainly quadrupedal *Cheirogaleus*-like behavioral repertoire, while *Absarokius* as well as *Arapahovius, Shoshonius, Washakius, Omomys* and *Ourayia* (“omomyines”) appear more similar to *Microcebus* and other more leaping–reliant extant lemuriforms. Other authors have come to similar conclusions based on studies that sampled a greater diversity of elements from within the skeleton (like Gebo), including Anemone and Covert [116], Covert and Hamrick [117] and Covert [118]. These patterns reveal more explanatory power for inferring behavioral differences from calcaneal elongation than previously recognized. For instance, Gebo [30] and Dagosto et al. [36] concluded that calcaneal elongation did not differentiate anaptomorphines and omomyines, as we would have also done without taking into account allometric patterns.

##### Note added in press *Archicebus Achilles*

Ni and colleagues [38] recently described *Archicebus achilles*, the most complete associated and semi-articulated partial skeleton and skull of an omomyiform. The specimen is from the early Eocene of China and may be close in age to some species of *Teilhardina. A. achilles* is similar to *Teilhardina* in morphology, in dimensions of the dentition, skull, and orbits; and in estimated body mass (e.g., see tables S3–4 of [38] and figure S9 of [38]). Nevertheless, the phylogenetic analysis of Ni et al. [38] recovers this taxon as a more basal species of omomyiform than any species of *Teilhardina* based on subtle differences in dental features interpreted as more primitive (larger P_1_, smaller P_4_ metaconid, and low-crowned lower premolars) and a shorter, broader distal calcaneal segment. If *A. achilles* is closer to the ancestor of tarsiiforms than species of *Teilhardina*, then this specimen provides a test of the morphological hypothesis generated by our ancestral state reconstructions (ASR), and allows further consideration of our proposal that patterns of calcaneal elongation reflect the importance of leaping behaviors in early primate evolution. The observation by Ni et al. [38] that *A. achilles* has a distal calcaneus that is shorter than in *Teilhardina* runs contrary to the predictions of our ASRs, which show the ancestral tarsiiform to be essentially identical to *Teilhardina* in calcaneal proportions.

To evaluate the calcaneal morphology of *A. achilles* for ourselves, we compared the measurements taken on *A. achilles* by Ni et al. [38] (Table S2 in [38]) to those we took on *Teilhardina belgica* and reported here (Table S1 in File S1). In fact, the measurements given for *A. achilles* (TL = 6.5, DL = 3.39, CW = 1.76, CD = 1.28) are almost identical to those measured by us for *T. belgica* IRSNB M1237 prior to the publication of [38] (Table S1 in File S1: TL = 6.52, DL = 3.377, CW = 1.58, CD = 1.11). While the cuboid facet measures for *A. achilles* are slightly larger than those of IRSNB M1237, we have noticed a similar discrepancy between our measurements of cuboid facet dimensions on *T. belgica* and those of Gebo et al. [119] on the same specimens (compare our Table S1 in File S1 to table 6 in [119]).

Of course, our ASRs refer to the calcaneal elongation index, not absolute length of the distal calcaneal segment. The calcaneal elongation index for *A. achilles* based on these measures (52% or −0.654 as log-transformed ratio) is slightly greater than that for IRSNB M1237. In terms of residual values, *A. achilles* is calculated at 0.01 (compare to “Res A” of Tables 1–2; Figs. 9A, 11). This is higher than the average value for *T. belgica* (0.002) (Table 2, Res A; Figs. 9A, 11). IRSNB M1247 has the highest residual of any *T. belgica* individual we measured, and its value is 0.01, identical to that of *A. achilles*. However, we note that residual values are affected by mass estimates, and our regressions using the calcaneal cuboid facet give a higher estimate of mass in *A. achilles* (62 g) than obtained by Ni et al. [38] (20–30 g). This value is also slightly greater than our average estimate for *T. belgica* (47.25 g: see Table S1 in File S1). Several pieces of evidence suggest that Ni et al. [38] underestimate the mass of both *Teilhardina* and *Archicebus* by a small, but (in this context) important margin: 1) They rely partly on Gingerich’s [120] “tarsioid” regression, which is not actually an empirical result based on independent data, but is a composite that assumes the slope of his “non-tariser primate” regression and extrapolates an intercept using body mass and tooth dimensions of *Tarsius* alone - using this line assumes all tarsiiforms have the greatly enlarged teeth of modern *Tarsius*, which is not necessarily justified since this is likely an adaptation for the unusual tarsier habit of strict faunivory, not likely shared by most omomyiforms; 2) their skull width and body length data show *A. achilles* to be slightly larger than *Microcebus berthae* which ranges up to 38 g according to their sources; 3) the cuboid facet dimensions they report for *A. achilles* match our measurements for *Microcebus griseorufus* (Table S1 in File S1) and our body mass estimates for *M. griseorufus* at 59–72 g (Table S1 in File S1) are correct to within about 5% of species/sex means. On the other hand, if we had been able to take measurements on the cuboid facet directly rather than using values published by Ni et al. [38], we expect those values would have been slightly smaller and indicated a body mass in the 40–50 g range (overlapping with our estimates for *T. belgica*). Regardless of the exact body mass, it is clear that *T. belgica* and *A. achilles* are extremely similar in size. Given their identical calcaneal proportions, they should share a similar calcaneal elongation residual.

Therefore, the morphology of *A. achilles* actually matches very closely the predictions of the hypothesis generated by our ancestral state reconstructions as it plots within the limits of the mean estimates for the ancestral tarsiiform (Fig. 9A). Furthermore, the presence of a low intermembral index, and leaping features in the femur of *A. achilles* as noted by [38], are consistent with our suggestion that calcaneal elongation increased as a result of consistent pressure for effective leaping in early euprimate evolution.

### What is Leaping for?

A careful consideration of the results of this study suggests strongly that there was selection for increased calcaneal elongation, and thus arguably improved leaping, leading up to and extending into early euprimate evolution. What aspect of the environment of early euprimates selected for these changes? In other words – what was/is the biological role [121] of leaping? The possibilities are numerous. Crompton and others have provided a compelling argument for predator avoidance being a primary driver, based on studies of the context in which maximum leaping performance is utilized in extant prosimians [122]. Leaping might also provide a more energy efficient way of navigating a discontinuous foraging environment [3], [8], [9]. Finally, leaping might have evolved as part of a predatory ambush pattern as is utilized by some insectivorous primates today. It seems likely that all of these factors (and more) have either helped to maintain, or selected for improvements in leaping proficiency in different primate lineages throughout the clade’s evolution. Determining which, if any, of these factors dominated at the beginning of primate evolution is beyond the scope of this paper. Such a question might ultimately be addressable with a more complete picture of changes in early primate and euprimate morphology in combination with information on fine scale changes in the environmental context of these animals. Such a picture can only be generated with paleoenvironment reconstructions (including community structure) that have fine temporal resolution, and with more complete anatomical and taxonomic sampling of early primates.

### Conclusion

Returning to our original questions, we conclude that there is a consistent relationship between calcaneal elongation and body mass among primates as a whole, in which larger taxa have predictably lower degrees of calcaneal elongation. Behavioral differences for more acrobatic leaping are associated with greater calcaneal elongation at all body sizes, while slow, cautious climbing and terrestriality is associated with lower calcaneal elongation in prosimians with a tarsifulcrumating foot. Anthropoids do not have a leaping related signal imposed on allometric variation in the calcaneus, probably due to the evolutionarily-frequent anatomical departure from a tarsifulcrumating foot. However, arboreal quadrupedal anthropoids have a more elongate ankle than anthropoid slow-climbers or terrestrialists. Although variance in calcaneal elongation among fossil taxa correlates better with previously suggested behavioral differences for these same species when taking allometry into account, strong phylogenetic covariance in size-“corrected” calcaneal elongation makes it difficult to reconstruct locomotor behavior by pure analogy to extant forms. This strong phylogenetic covariance and ASRs showing that various taxa must have had ancestors first beginning to specialize in leaping at substantially different body sizes helps explains why today some similarly-sized, leaping reliant taxa (e.g., *Otolemur* and *Avahi*) have very different degrees of calcaneal elongation.

Initial increases in calcaneal elongation during the euprimateform-euprimate transition may have been due to the acquisition of a grasping hallux and tarsifulcrumating foot, as suggested by the presence of a grasping hallux and a more elongate distal calcaneus in the stem primate *Carpolestes simpsoni*, which may represent either the ancestral state for euprimates or a parallel acquisition under very similar conditions. Subsequent increases in calcaneal elongation occur in parallel among stem haplorhines and stem strepsirrhines and are best explained by *persistent selection for* and *improved performance in* acrobatic leaping ability. We also note that even in the case in which calcaneal elongation increases appear related to increased grasping ability and to “recovery of lost load arm” this would actually imply selection for *maintenance* of some critical amount of leaping ability during the euprimate transition – meaning that either way, leaping behaviors were important at the origin of Euprimates. Clearly increases in calcaneal elongation are not a *de facto* consequence of increased hallucal specialization as illustrated by the lack of elongation in the didelphid *Caluromys* as compared to *Monodelphis.*

Finally, even though it seems justifiable to conclude from patterns of calcaneal elongation observed in this study that there was some selection for more agile behavior over the course of the euprimateform-euprimate transition, the first animals to benefit from improvements in leaping ability would not likely have been particularly good leapers by modern standards. This is supported by the mechanical consequences of their still relatively short limbs and the lack of a I-II pedal grasp type in larger forms like *Cantius.* Accordingly, regardless of the importance of leaping behaviors to their fitness, the earliest sampled euprimates *Teilhardina* and *Cantius* (and *Archicebus*) most likely had leaping abilities that were more closely analogous to those of generalized arboreal quadrupeds like the dwarf lemurs (*Cheirogaleus*) based on similar calcaneal proportions and other proportional similarities throughout the skeleton. Therefore, based on these data, we are hesitant to speculate on the degree to which large forward-facing eyes (which presumably evolved during the euprimateform-euprimate transition) were adaptive in the context of a suite of features allowing highly acrobatic leaping. Nevertheless, prior advancements in the visual system were likely critical for subsequent improvements in leaping behavior during early euprimate evolution. Finally, the parallel acquisition of leaping specialization in haplorhines and strepsirrhines hints at the possibility of parallel acquisition of other features that can be functionally related to leaping, even including those features that have been thought of as euprimate synapomorphies in the past.

## Supporting Information

Figure S1 (TIF)

File S1 Supporting Infromation Tables S1–S7.
(DOC)
