# Correction: Evolution and Allometry of Calcaneal Elongation in Living and Extinct Primates

**DOI:** 10.1371/annotation/77784e61-3a43-4615-85bd-b6b4369568fd

**Published:** 2013-09-10

**Authors:** Doug M. Boyer, Erik R. Seiffert, Justin T. Gladman, Jonathan I. Bloch

The Reference for the image used in Figure 1A was incorrectly omitted. The reference is: "Kingdon, J. 1971. East African Mammals: An Atlas of Evolution in Africa. Vol. I: Primates. Academic Press, London, 446 pp." 

