# Supplementary figures and images for: Evolution and Allometry of Calcaneal Elongation in Living and Extinct Primates
Source: PLoS One. 2013 Jul 3;8(7):e67792. doi: 10.1371/journal.pone.0067792 (PMC3701013; doi:10.1371/journal.pone.0067792)

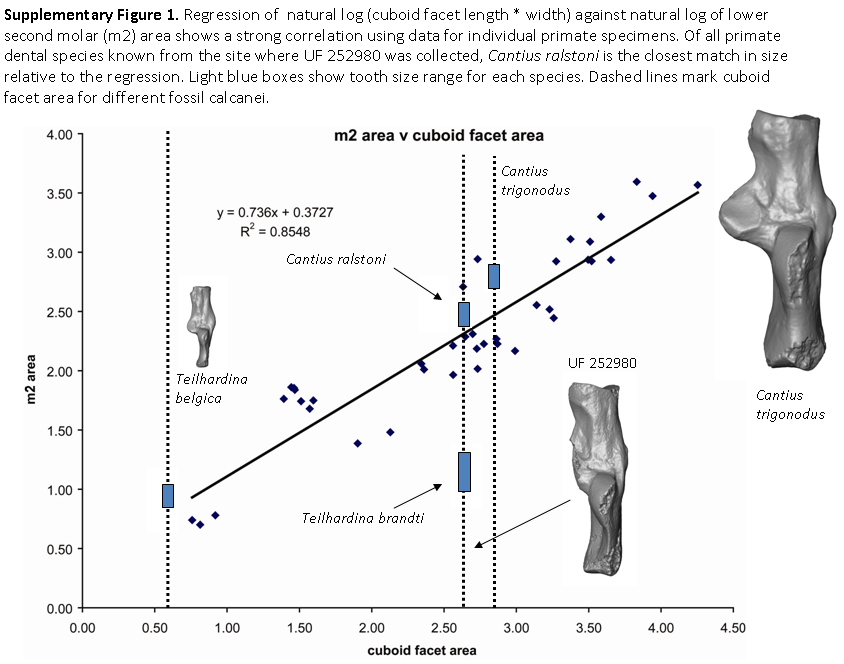

Supplement: Figure S1 — (TIF) [file pone.0067792.s001.tif]
