# Supplementary material for: Evolution and Allometry of Calcaneal Elongation in Living and Extinct Primates
Source: PLoS One. 2013 Jul 3;8(7):e67792. doi: 10.1371/journal.pone.0067792 (PMC3701013; doi:10.1371/journal.pone.0067792)
Supplement: File S1 — Supporting Infromation Tables S1–S7. (DOC) [file pone.0067792.s002.doc]

Supplemental Table S1. Raw Data (see foot note at bottom for table information).

| **Taxon** | **Res** | **Scan** | **sex** | **Catalogue** | **ML** | **CW** | **CD** | **ln(CA)** | **bm** | **bm reg** | **est ln(bm)** | **antilog (bm)** | **DL** | **TL** | **ln(CE)** |
| --- | --- | --- | --- | --- | --- | --- | --- | --- | --- | --- | --- | --- | --- | --- | --- |
| *Cantius ralstoni* | 20.735 | 3 | NA | UM 79150 | 50 | 3.89 | 2.965 | 2.445 | NA | - | 6.270 | 545 | 7.25 | - | - |
| *Cantius ralstoni* | 20.735 | 3 | NA | UM SLC VC Msc6 | 50 | 3.68 | 2.695 | 2.294 | NA | - | 6.069 | 446 | 7.22 | - | - |
| *Cantius ralstoni* | 20.5 | 3 | NA | UF 252980 | 50 | 3.91 | 2.635 | 2.332 | NA | - | 6.120 | 469 | 7.160 | 15.765 | -0.789 |
| *Cantius mckennai* | 28.737 | 3 | NA | USGS 5897 | 210 | 3.53 | 3.435 | 2.495 | NA | - | 6.336 | 582 | 7.640 | 17.830 | -0.847 |
| *Cantius mckennai* | 32.693 | 3 | NA | USGS 25029B | 250 | 4.74 | 3.44 | 2.792 | NA | - | 6.729 | 862 | 8.145 | 19.430 | -0.869 |
| *Cantius mckennai* | 32.693 | 3 | NA | USGS 25029A | 250 | 4.29 | 2.86 | 2.507 | NA | - | 6.352 | 591 | 8.730 | 20.010 | -0.829 |
| *Cantius trigonodus* | 32.707 | 3 | NA | USGS 6769 | 448 | 4.84 | 3.885 | 2.934 | NA | - | 6.918 | 1042 | 8.575 | - | - |
| *Cantius trigonodus* | 32.707 | 3 | NA | USGS 6765 | 444 | 4.675 | 4.15 | 2.965 | NA | - | 6.960 | 1086 | 8.815 | - | - |
| *Cantius trigonodus* | 30.439 | 3 | NA | USGS 21829 | 409 | 4.995 | 4.185 | 3.040 | NA | - | 7.059 | 1199 | 8.995 | 21.285 | -0.861 |
| *Cantius trigonodus* | 32.153 | 3 | NA | AMNH 16852 | ? | 4.78 | 3.27 | 2.749 | NA | - | 6.673 | 815 | 9.050 | 21.950 | -0.886 |
| *Cantius trigonodus* | 28.737 | 3 | NA | USGS 21767 | 409 | 3.685 | 3.18 | 2.461 | NA | - | 6.291 | 556 | 9.060 | 20.030 | -0.793 |
| *Cantius trigonodus* | Na | Na | NA | USGS 21765 | 420 | 4.905 | 3.595 | 2.870 | NA | - | 6.833 | 957 | 10.035 | 23.295 | -0.842 |
| *Cantius frugivorus* | 32.707 | 3 | NA | USGS 21776 | 546 | 4.305 | 3.525 | 2.720 | NA | - | 6.634 | 784 | 7.905 | - | - |
| *Cantius frugivorus* | 29.591 | 3 | NA | USGS 21828 | 556 | 4.175 | 3.86 | 2.780 | NA | - | 6.714 | 849 | 7.700 | 18.940 | -0.900 |
| *Cantius frugivorus* | 32.199 | 3 | NA | USGS 6792 | 556 | 5.03 | 2.945 | 2.696 | NA | - | 6.602 | 759 | 8.310 | 19.950 | -0.876 |
| *Cantius abditus* | 29.591 | 3 | NA | USGS 21774 | 541 | 5.24 | 3.735 | 2.974 | NA | - | 6.972 | 1099 | 9.005 | 22.445 | -0.913 |
| *Cantius abditus* | Na | Na | NA | USGS 21775 | 550 | 4.78 | 3.405 | 2.790 | NA | - | 6.727 | 860 | 9.180 | 22.085 | -0.878 |
| *Cantius abditus* | 44.544 | 3 | NA | USGS 21827 | 463 | 5.55 | 4.82 | 3.287 | NA | - | 7.386 | 1664 | 9.400 | 23.635 | -0.922 |
| *Cantius abditus* | 35.083 | 3 | NA | USGS 21825 | 561 | 5.12 | 3.55 | 2.900 | NA | - | 6.873 | 996 | 9.555 | 23.025 | -0.880 |
| *Cantius abditus* | 29.591 | 3 | NA | USGS 21771 | 463 | 5.79 | 3.67 | 3.056 | NA | - | 7.081 | 1226 | 9.890 | 23.895 | -0.882 |
| *Cantius abditus* | 30.439 | 3 | NA | USGS 6783 | 481 | 6 | 4.065 | 3.194 | NA | - | 7.264 | 1472 | 9.890 | 23.415 | -0.862 |
| *Notharctus* sp. | 32.283 | 3 | NA | AMNH 1727 | - | 7.65 | 5.76 | 3.786 | NA | - | 8.049 | 3227 | 9.995 | 24.600 | -0.901 |
| *Notharctus* sp*.* | 30.126 | 3 | NA | AMNH 131956 | - | 7.26 | 4.64 | 3.517 | NA | - | 7.692 | 2259 | 10.110 | 23.940 | -0.862 |
| *Notharctus* sp*.* | 30.126 | 3 | NA | AMNH 131955 | - | 7.07 | 4.46 | 3.451 | NA | - | 7.605 | 2070 | 9.830 | 24.440 | -0.911 |
| *Notharctus* sp*.* | 28.933 | 3 | NA | AMNH 55061 | - | 6.105 | 4.975 | 3.414 | NA | - | 7.555 | 1969 | 9.050 | 22.365 | -0.905 |
| *Notharctus* sp*.* | 30.0 | 2 | NA | AMNH 11474 | - | 6.095 | 4.49 | 3.309 | NA | - | 7.417 | 1715 | 9.535 | 22.625 | -0.864 |
| *Notharctus* sp*.* | 28.933 | 3 | NA | AMNH 13766 | - | 6.64 | 5.37 | 3.574 | NA | - | 7.768 | 2436 | 9.665 | 25.000 | -0.950 |
| *Notharctus* sp*.* | 41.518 | 3 | NA | AMNH 131945 | - | 8.06 | 5.76 | 3.838 | NA | - | 8.118 | 3458 | 10.530 | 25.470 | -0.883 |
| *Notharctus* sp*.* | 28.248 | 3 | NA | AMNH 11478 | - | 7.52 | 6 | 3.809 | NA | - | 8.080 | 3330 | 10.590 | 26.670 | -0.924 |
| *Notharctus* sp*.* | 32.153 | 3 | NA | AMNH 129382 | - | 5.705 | 4.755 | 3.301 | NA | - | 7.405 | 1695 | 10.095 | 23.530 | -0.846 |
| *Smilodectes* sp*.* | 30.902 | 3 | NA | AMNH 131774 | - | 7.18 | 4.91 | 3.563 | NA | - | 7.753 | 2400 | 10.130 | 25.020 | -0.904 |
| *Smilodectes* sp*.* | 29.929 | 3 | NA | AMNH 131763 | - | 7.79 | 5.04 | 3.670 | NA | - | 7.896 | 2769 | 9.780 | 24.820 | -0.931 |
| *Marcgodinotius indicus* | 15.0 | 1 | NA | GU 709 | - | 2.38 | 1.67 | 1.380 | NA | - | 4.856 | 132 | 3.060 | 7.600 | -0.910 |
| *Marcgodinotius indicus* | 15.0 | 1 | NA | GU 751 | - | 2.61 | 1.78 | 1.536 | NA | - | 5.063 | 163 | 3.390 | 8.250 | -0.889 |
| *Marcgodinotius indicus* | 15.0 | 1 | NA | GU 1644 | - | 2.19 | 1.68 | 1.303 | NA | - | 4.753 | 120 | 2.910 | 6.950 | -0.871 |
| *Marcgodinotius indicus* | 15.0 | 1 | NA | GU 1643 | - | 2.08 | 1.67 | 1.245 | NA | - | 4.677 | 111 | 3.210 | 7.560 | -0.857 |
| *Marcgodinotius indicus* | 15.0 | 1 | NA | GU 710 | - | 2.13 | 1.52 | 1.175 | NA | - | 4.583 | 101 | 2.970 | 6.750 | -0.821 |
| *Asiadapis cambayensis* | 15.0 | 1 | NA | GU 760 | - | 2.98 | 2.78 | 2.114 | NA | - | 5.830 | 351 | 4.260 | 10.900 | -0.939 |
| *Adapis parisiensis* | 32.153 | 3 | NA | AMNH 10016 | - | 5.48 | 3.52 | 2.960 | NA | - | 6.952 | 1078 | 5.490 | 18.780 | -1.230 |
| *Adapis parisiensis* | 20.5 | 2 | NA | NMB QE 644 | - | 5.48 | 3.45 | 2.939 | NA | - | 6.926 | 1050 | 5.180 | 17.550 | -1.220 |
| *Adapis parisiensis* | 20.5 | 2 | NA | NMB QE 741 | - | 5.56 | 3.48 | 2.963 | NA | - | 6.956 | 1082 | 4.500 | 16.450 | -1.296 |
| *Adapis parisiensis* | 20.5 | 2 | NA | NMB QE 779 | - | 5.73 | 3.33 | 2.949 | NA | - | 6.938 | 1062 | 5.230 | 18.110 | -1.242 |
| *Adapis parisiensis* | 20.5 | 2 | NA | NMB QF 558 | - | 5.34 | 3.51 | 2.931 | NA | - | 6.914 | 1038 | 4.260 | 15.100 | -1.265 |
| *Adapis parisiensis* | 20.5 | 2 | NA | NMB QH 640 | - | 5.69 | 3.92 | 3.105 | NA | - | 7.145 | 1307 | 5.580 | 18.380 | -1.192 |
| *Adapis* sp*.* | 20.5 | 2 | NA | NMB QE 530 | - | 6.91 | 4.94 | 3.530 | NA | - | 7.710 | 2299 | 7.210 | 22.080 | -1.119 |
| *Leptadapis magnus* | 30.0 | 2 | NA | NMB QW 1676 | - | 10.41 | 6.44 | 4.205 | NA | - | 8.606 | 5633 | 11.600 | 34.200 | -1.081 |
| *Leptadapis magnus* | 30.0 | 2 | NA | NMB QE 604 | - | 9.05 | 6.51 | 4.076 | NA | - | 8.434 | 4745 | 10.530 | 31.600 | -1.099 |
| *Leptadapis magnus* | 30.0 | 2 | NA | NMB QE 830 | - | 10.31 | 7.12 | 4.296 | NA | - | 8.726 | 6353 | 10.320 | 31.580 | -1.118 |
| *Leptadapis magnus* | 30.0 | 2 | NA | NMB QE 920 | - | 9.6 | 6.82 | 4.182 | NA | - | 8.574 | 5458 | 8.850 | 33.380 | -1.328 |
| *Leptadapis magnus* | 30.0 | 2 | NA | NMB QF 421 | - | 9.94 | 7.09 | 4.255 | NA | - | 8.672 | 6019 | 9.260 | 32.350 | -1.251 |
| *Leptadapis magnus* | Na | Na | NA | ACQ 265 | - | 10.26 | 7.48 | 4.340 | NA | - | 8.785 | 6740 | 10.110 | 34.450 | -1.226 |
| *Leptadapis magnus* | Na | Na | NA | ACQ 266 | - | 10.35 | 8.55 | 4.483 | NA | - | 8.974 | 8142 | 10.280 | 34.820 | -1.220 |
| *Leptadapis magnus* | Na | Na | NA | PQ 1746 | - | 10.92 | 8.73 | 4.557 | NA | - | 9.073 | 8988 | 10.760 | 34.260 | -1.158 |
| *Leptadapis magnus* | Na | Na | NA | PQ 1747 | - | 11.06 | 8.61 | 4.556 | NA | - | 9.072 | 8975 | 11.100 | 36.260 | -1.184 |
| *Leptadapis magnus* | 30.0 | 2 | NA | ACQ 267 | - | 10.73 | 7.98 | 4.450 | NA | - | 8.931 | 7794 | 10.120 | 34.470 | -1.226 |
| *Leptadapis magnus* | Na | Na | NA | ACQ 268 | - | 9.53 | 6.98 | 4.197 | NA | - | 8.596 | 5575 | 8.850 | 28.250 | -1.161 |
| *Anchomomys frontanyensis* | Na | Na | NA | IPS 7748 | - | 2.22 | 1.7 | 1.328 | NA | - | 4.787 | 124 | 5.520 | 10.900 | -0.680 |
| *Anchomomys frontanyensis* | Na | Na | NA | IPS 7769 | - | 2.07 | 1.7 | 1.258 | NA | - | 4.694 | 113 | 5.200 | 9.900 | -0.644 |
| *Teilhardina belgica* | 6.90 | 3 | NA | IRSNB M1247 | - | 1.46 | 1.21 | 0.569 | NA | - | 3.779 | 45 | 3.580 | 6.740 | -0.633 |
| *Teilhardina belgica* | Na | Na | NA | IRSNB M1236 | - | 1.36 | 1.37 | 0.626 | NA | - | 3.855 | 49 | 3.972 | 7.390 | -0.621 |
| *Teilhardina belgica* | Na | Na | NA | IRSNB 16786-03 | - | 1.58 | 1.21 | 0.648 | NA | - | 3.884 | 50 | 3.580 | 6.870 | -0.652 |
| *Teilhardina belgica* | Na | Na | NA | IRSNB M1237 | - | 1.58 | 1.11 | 0.562 | NA | - | 3.77 | 45 | 3.377 | 6.520 | -0.658 |
| *Arapahovius gazini* | 10.0 | 1 | NA | UCM 67850 | - | 1.66 | 1.35 | 0.807 | NA | - | 4.095 | 62 | 4.960 | 8.380 | -0.524 |
| *Absarokius* sp*.* | 10.0 | 1 | NA | UCM 67907 | - | 2.22 | 1.39 | 1.127 | NA | - | 4.520 | 95 | 5.640 | 10.390 | -0.611 |
| *Omomys carteri* | 26.22 | 3 | NA | UCM 67679 | - | 3.29 | 2.62 | 2.154 | NA | - | 5.883 | 370 | 7.440 | 14.430 | -0.662 |
| *Omomys carteri* | 17.063 | 3 | NA | UCM 68745 | - | 3.02 | 2.70 | 2.099 | NA | - | 5.809 | 344 | 8.310 | 15.580 | -0.629 |
| *Omomys carteri* | 26.22 | 3 | NA | UCM 69065 | - | 3.46 | 2.73 | 2.246 | NA | - | 6.005 | 418 | 8.360 | 15.620 | -0.625 |
| *Omomys carteri* | 10.0 | 1 | NA | UCM 67678 | - | 3.41 | 2.28 | 2.051 | NA | - | 5.746 | 323 | 7.610 | 14.270 | -0.629 |
| *Washakius insignis* | 14.937 | 3 | NA | AMNH 88824 | - | 2.27 | 1.76 | 1.385 | NA | - | 4.862 | 133 | 5.570 | 10.420 | -0.626 |
| *Shoshonius cooperi* | 12.152 | 3 | NA | CM 69765 | - | 2.12 | 1.57 | 1.202 | NA | - | 4.620 | 105 | 5.470 | 10.160 | -0.619 |
| *Hemiacodon gracilis* | 17.925 | 3 | NA | AMNH 12613 | - | 3.77 | 2.65 | 2.302 | NA | - | 6.079 | 450 | 7.620 | 14.900 | -0.671 |
| *Omomys carteri* | 10.0 | 1 | NA | UM 98604 | - | 3.07 | 2.49 | 2.034 | NA | - | 5.724 | 315 | 7.290 | 14.120 | -0.661 |
| *Ourayia uintensis* | 20.5 | 2 | NA | SDNH 4020-60933 | - | 5.51 | 3.81 | 3.044 | NA | - | 7.065 | 1206 | 10.810 | 22.250 | -0.722 |
| *Necrolemur zitteli* | 18.88 | 3 | NA | PMZ A/V 637 | - | na | na | na | NA | - | 5.559 | 267 | 13.030 | 19.520 | -0.404 |
| *Tetonius homunculus* | 14.924 | 3 | NA | AMNH 88820-21# | - | na | na | na | NA | - | 4.403 | 84 | 4.860 | 9.640 | -0.685 |
| *Omomys* sp*.?* | 17.925 | 3 | NA | AMNH 29164 | - | 3.24 | 2.45 | 2.072 | NA | - | 5.774 | 332 | 8.290 | 15.030 | -0.595 |
| *Tarsius bancanus* | 20.5 | 2 | M | AMNH 106754 | - | 2.38 | 1.78 | 1.444 | 128 | - | 4.940 | 144 | 20.770 | 27.040 | -0.264 |
| *Tarsius bancanus* | 20.5 | 2 | F | AMNH 106649 | - | 2.34 | 1.72 | 1.392 | 117 | - | 4.872 | 135 | 19.430 | 24.910 | -0.248 |
| *Tarsius spectrum* | 20.5 | 2 | F | AMNH 109367 | - | 2.52 | 1.91 | 1.571 | 108 | - | 5.110 | 171 | 20.030 | 26.590 | -0.283 |
| *Tarsius spectrum* | 20.5 | 2 | F | AMNH 109368 | - | 2.45 | 1.85 | 1.511 | 108 | - | 5.030 | 158 | 19.140 | 25.050 | -0.269 |
| *Tarsius spectrum* | 20.5 | 2 | F | AMNH 109369 | - | 2.42 | 2.04 | 1.597 | 108 | - | 5.143 | 177 | 19.520 | 26.070 | -0.289 |
| *Tarsius syrichta* | 20.5 | 2 | M | AMNH 203297 | - | 2.26 | 1.91 | 1.462 | 134 | - | 4.965 | 148 | 17.620 | 23.620 | -0.293 |
| *Tarsius syrichta* | 20.5 | 2 | F | AMNH 203296 | - | 2.38 | 1.825 | 1.469 | 117 | - | 4.973 | 149 | 18.180 | 24.300 | -0.290 |
| *Tarsius syrichta* | 20.5 | 2 | M | AMNH 166856 | - | 2.2 | 2.117 | 1.538 | 134 | - | 5.066 | 163 | 18.740 | 24.900 | -0.284 |
| *Galagoides demidoff* | 15.0 | 1 | ? | AMNH 212958 | - | 1.75 | 1.21 | 0.750 | 70.5* | - | 4.020 | 57 | 14.020 | 18.060 | -0.253 |
| *Galagoides demidoff* | 15.0 | 1 | ? | AMNH 212956 | - | 2.15 | 1.42 | 1.116 | 70.5* | - | 4.505 | 93 | 15.310 | 19.910 | -0.263 |
| *Galagoides demidoff* | 15.0 | 1 | F | AMNH 241121 | - | 2.17 | 1.43 | 1.132 | 70.5* | - | 4.527 | 95 | 15.090 | 19.980 | -0.281 |
| *Galago senegalensis* | 20.5 | 2 | M | AMNH 187358 | - | 3.13 | 2.2 | 1.929 | 271 | - | 5.585 | 275 | 22.490 | 30.220 | -0.295 |
| *Galago senegalensis* | 15.0 | 1 | M | AMNH 35445 | - | 2.54 | 2.7 | 1.925 | 271 | - | 5.580 | 273 | 18.750 | 26.270 | -0.337 |
| *Galago senegalensis* | 20.5 | 2 | F | AMNH 87064 | - | 3.02 | 2.45 | 2.001 | 248 | - | 5.680 | 302 | 19.580 | 27.330 | -0.333 |
| *Galago senegalensis* | 20.5 | 2 | F | AMNH 119521 | - | 2.6 | 2.58 | 1.903 | 248 | - | 5.550 | 265 | 19.440 | 27.170 | -0.335 |
| *Galago senegalensis* | 20.5 | 2 | M | AMNH 70410 | - | 2.42 | 2.84 | 1.928 | 271 | - | 5.582 | 274 | 18.700 | 25.700 | -0.318 |
| *Otolemur crassicaudatus* | 20.5 | 2 | M | AMNH 216239 | - | 5.93 | 4.26 | 3.229 | 1510+ | - | 7.310 | 1542 | 22.150 | 34.960 | -0.456 |
| *Otolemur crassicaudatus* | 39.0 | 2 | M | AMNH 216244 | - | 5.9 | 4.41 | 3.259 | 1510+ | - | 7.350 | 1604 | 22.530 | 35.080 | -0.443 |
| *Otolemur crassicaudatus* | 20.5 | 2 | F | AMNH 216241 | - | 5.72 | 4.24 | 3.189 | 1258+ | - | 7.256 | 1461 | 21.540 | 33.550 | -0.443 |
| *Otolemur crassicaudatus* | 25.0 | 2 | M | AMNH 80801 | - | 6.413 | 4.13 | 3.277 | 1510+ | - | 7.373 | 1642 | 22.830 | 35.430 | -0.439 |
| *Otolemur garnetti* | 20.5 | 2 | ? | DPC osteo#011 | - | 4.48 | 4.15 | 2.923 | 764 | - | 6.903 | 1026 | 21.510 | 33.140 | -0.432 |
| *Otolemur garnetti* | 20.5 | 2 | ? | DPC 022 | - | 4.64 | 4.01 | 2.924 | 764 | - | 6.904 | 1027 | 19.280 | 30.550 | -0.460 |
| *Otolemur garnetti* | 20.5 | 2 | ? | DPC 1083 | - | 4.42 | 4.04 | 2.882 | 764 | - | 6.850 | 973 | 21.330 | 32.390 | -0.418 |
| *Euoticus elegantulus* | 18.0 | 1 | M | AMNH 241127 | - | 3.27 | 2.57 | 2.129 | 274 | - | 5.849 | 358 | 14.140 | 22.210 | -0.452 |
| *Komba robustus* | 18.0 | 1 | NA | KNM-SO 1364 | - | 3.1 | 2.4 | 2.007 | NA | - | 5.688 | 304 | 7.200 | 12.560 | -0.556 |
| *Microcebus griseorufus* | 10.0 | 1 | F | AMNH 174415 | - | 1.71 | 1.32 | 0.814 | 63 | - | 4.104 | 62 | 6.420 | 10.000 | -0.443 |
| *Microcebus griseorufus* | 10.0 | 1 | F | AMNH 174430 | - | 1.51 | 1.66 | 0.919 | 63 | - | 4.244 | 72 | 6.080 | 9.940 | -0.492 |
| *Microcebus griseorufus* | 10.0 | 1 | F | AMNH 174431 | - | 1.68 | 1.33 | 0.804 | 63 | - | 4.091 | 62 | 6.380 | 10.260 | -0.475 |
| *Microcebus griseorufus* | 10.0 | 1 | M | AMNH 174471 | - | 1.57 | 1.36 | 0.759 | 59 | - | 4.031 | 58 | 5.590 | 9.190 | -0.497 |
| *Mirza coquereli* | 15.0 | 1 | ? | DPC 1139 | - | 2.73 | 2.69 | 1.994 | 315 | - | 5.670 | 299 | 8.110 | 15.190 | -0.628 |
| *Mirza coquereli* | 15.0 | 1 | ? | DPC 0137 | - | 2.7 | 2.6 | 1.949 | 315 | - | 5.611 | 282 | 8.100 | 14.560 | -0.586 |
| *Cheirogaleus major* | 20.5 | 2 | ? | AMNH 100640 | - | 3.38 | 2.38 | 2.085 | NA | - | 5.791 | 338 | 6.830 | 14.420 | -0.747 |
| *Cheirogaleus medius* | 18.0 | 1 | M | AMNH 80072 | - | 2.85 | 2.45 | 1.943 | 283 | - | 5.603 | 280 | 6.120 | 12.450 | -0.710 |
| *Cheirogaleus medius* | 13.026 | 3 | ? | DPC 031 | - | 2.7 | 2.03 | 1.701 | 282.5 | - | 5.282 | 203 | 4.720 | 9.780 | -0.729 |
| *Cheirogaleus medius* | 15.0 | 1 | ? | DPC 0142 | - | 2.68 | 2.16 | 1.756 | 282.5 | - | 5.355 | 218 | 4.840 | 9.950 | -0.721 |
| *Cheirogaleus medius* | 15.0 | 1 | ? | DPC 1023 | - | 2.84 | 2.2 | 1.832 | 282.5 | - | 5.456 | 241 | 5.140 | 10.190 | -0.684 |
| *Daubentonia madagascariensis* | 39.0 | 2 | F | AMNH 185643 | - | 7.74 | 4.99 | 3.654 | 2490 | - | 7.874 | 2709 | 9.560 | 23.340 | -0.893 |
| *Eulemur fulvus albifrons* | 25.0 | 2 | F | AMNH 170708 | - | 6.1 | 4.52 | 3.317 | 2077 | - | 7.427 | 1732 | 10.270 | 23.080 | -0.810 |
| *Eulemur fulvus albifrons* | 25.0 | 2 | F | AMNH 170717 | - | 6.64 | 4.33 | 3.359 | 2077 | - | 7.482 | 1831 | 10.320 | 22.870 | -0.796 |
| *Eulemur fulvus albifrons* | 35.0 | 2 | F | AMNH 170723 | - | 7.14 | 4.59 | 3.490 | 2077 | - | 7.656 | 2178 | 10.410 | 24.150 | -0.842 |
| *Eulemur fulvus albifrons* | 25.0 | 2 | F | AMNH 170728 | - | 6.65 | 4.82 | 3.467 | 2077 | - | 7.626 | 2115 | 10.040 | 22.900 | -0.825 |
| *Eulemur fulvus fulvus* | 35.0 | 2 | M | AMNH 31254 | - | 6.43 | 4.54 | 3.374 | 2000 | - | 7.502 | 1868 | 10.300 | 22.320 | -0.773 |
| *Eulemur fulvus mongoz* | 35.0 | 2 | M | AMNH 17403 | - | 5.7 | 4.64 | 3.275 | 2180 | - | 7.371 | 1639 | 9.410 | 20.860 | -0.796 |
| *Hapalemur griseus* | 20.5 | 2 | F | AMNH 170680 | - | 5.19 | 3.73 | 2.963 | 786.5 | - | 6.957 | 1083 | 9.100 | 19.720 | -0.773 |
| *Hapalemur griseus* | 20.5 | 2 | F | AMNH 170689 | - | 4.53 | 3.39 | 2.732 | 786.5 | - | 6.650 | 796 | 8.410 | 17.820 | -0.751 |
| *Hapalemur griseus* | 20.5 | 2 | M | AMNH 61589 | - | 4.86 | 2.86 | 2.632 | 867.5 | - | 6.517 | 698 | 7.850 | 17.220 | -0.786 |
| *Hapalemur griseus* | 20.5 | 2 | M | AMNH 170675 | - | 4.47 | 3.2 | 2.661 | 867.5 | - | 6.555 | 725 | 7.750 | 16.680 | -0.767 |
| *Prolemur simus* | Na | Na | NA | DPC 10926a | - | 7.06 | 5.73 | 3.700 | NA | - | 7.935 | 2881 | 11.090 | 25.620 | -0.837 |
| *Prolemur simus* | Na | Na | NA | DPC 10926b | - | 6.9 | 5.9 | 3.706 | NA | - | 7.944 | 2905 | 10.180 | 24.760 | -0.889 |
| *Prolemur simus* | Na | Na | NA | DPC 10926c | - | 6.9 | 5.46 | 3.629 | NA | - | 7.841 | 2621 | 11.280 | 25.570 | -0.818 |
| *Prolemur simus* | Na | Na | NA | DPC 10975a | - | 6.88 | 5.7 | 3.669 | NA | - | 7.894 | 2764 | 11.370 | 25.830 | -0.821 |
| *Prolemur simus* | 41.017 | 3 | NA | DPC 6818 | - | 6.73 | 5.57 | 3.624 | NA | - | 7.834 | 2604 | 10.700 | 24.250 | -0.818 |
| *Prolemur simus* | 30.0 | 2 | NA | DPC 6652a | - | 6.9 | 5.52 | 3.640 | NA | - | 7.855 | 2659 | 11.000 | 25.240 | -0.831 |
| *Prolemur simus* | 30.0 | 2 | NA | DPC 6652c | - | 7.71 | 5.77 | 3.795 | NA | - | 8.062 | 3268 | 10.040 | 24.370 | -0.887 |
| *Prolemur simus* | 41.017 | 3 | NA | DPC 10988a | - | 7.15 | 5.74 | 3.715 | NA | - | 7.955 | 2936 | 9.920 | 23.840 | -0.877 |
| *Prolemur simus* | 41.017 | 3 | NA | DPC 9925 | - | 6.82 | 5.02 | 3.533 | NA | - | 7.714 | 2308 | 10.040 | 23.920 | -0.868 |
| *Varecia variegata?* | 30.0 | 2 | NA | DPC 11843-B | - | 8.08 | 7.09 | 4.048 | NA | - | 8.397 | 4572 | 11.520 | 28.790 | -0.916 |
| *Varecia variegata* | 35.0 | 2 | F | AMNH 100512 | - | 8.56 | 5.39 | 3.832 | 3515 | √ | 8.110 | 3430 | 11.520 | 28.080 | -0.891 |
| *Varecia variegata* | 35.0 | 2 | F | AMNH 201384 | - | 9.23 | 5.58 | 3.942 | 3515 | √ | 8.256 | 3969 | 11.000 | 26.800 | -0.891 |
| *Lemur catta* | 35.0 | 2 | F | AMNH 22912 | - | 7.3 | 4.52 | 3.496 | 2210 | √ | 7.665 | 2198 | 10.440 | 23.800 | -0.824 |
| *Lemur catta* | 35.0 | 2 | M | AMNH 170739 | - | 7.58 | 4.46 | 3.521 | 2210 | √ | 7.697 | 2270 | 10.750 | 24.220 | -0.812 |
| *Lemur catta* | 25.0 | 2 | M | AMNH 170740 | - | 7.33 | 4.58 | 3.514 | 2210 | √ | 7.688 | 2249 | 10.110 | 23.860 | -0.859 |
| *Lepilemur mustelinus* | 20.5 | 2 | F | AMNH 170556 | - | 4.53 | 3.54 | 2.775 | 777 | √ | 6.707 | 843 | 8.970 | 18.770 | -0.738 |
| *Lepilemur mustelinus* | 20.5 | 2 | F | AMNH 170560 | - | 4.51 | 3.13 | 2.647 | 777 | √ | 6.538 | 712 | 8.550 | 18.180 | -0.754 |
| *Lepilemur mustelinus* | 20.5 | 2 | F | AMNH 170568 | - | 4.7 | 3.25 | 2.726 | 777 | √ | 6.643 | 791 | 9.960 | 20.150 | -0.705 |
| *Lepilemur mustelinus* | 20.5 | 2 | M | AMNH 170569 | - | 4.73 | 3.25 | 2.733 | 777 | √ | 6.651 | 797 | 8.970 | 18.240 | -0.710 |
| *Lepilemur mustelinus* | 20.5 | 2 | M | AMNH 170559 | - | 4.53 | 2.86 | 2.562 | 777 | √ | 6.424 | 635 | 8.750 | 17.910 | -0.716 |
| *Indri indri?* | 30.0 | 2 | NA | DPC 10975b | - | 8.74 | 7.54 | 4.188 | NA | - | 8.583 | 5506 | 13.600 | 31.070 | -0.826 |
| *Indri indri* | 39.0 | 2 | F | AMNH 100504 | - | 9.15 | 7.69 | 4.254 | 6335 | √ | 8.670 | 6006 | 15.410 | 34.980 | -0.820 |
| *Indri indri* | 39.0 | 2 | M | AMNH 208992 | - | 9.18 | 7.91 | 4.285 | 5830 | √ | 8.712 | 6262 | 14.760 | 33.770 | -0.828 |
| *Propithecus coquereli* | 35.0 | 2 | M | AMNH 208989 | - | 6.73 | 5.06 | 3.528 | 3700 | √ | 7.707 | 2292 | 10.000 | 24.080 | -0.879 |
| *Propithecus v. verreauxi* | 25.0 | 2 | F | AMNH 170474 | - | 6.95 | 5.19 | 3.585 | 2950 | √ | 7.783 | 2474 | 9.670 | 24.690 | -0.937 |
| *Propithecus v. verreauxi* | 39.0 | 2 | F | AMNH 170491 | - | 6.62 | 5.05 | 3.509 | 2950 | √ | 7.682 | 2237 | 10.000 | 25.020 | -0.917 |
| *Propithecus v. verreauxi* | 39.0 | 2 | F | AMNH 170471 | - | 7.21 | 5.2 | 3.624 | 2950 | √ | 7.834 | 2604 | 9.780 | 24.250 | -0.908 |
| *Avahi laniger* | 35.0 | 2 | M | AMNH 170461 | - | 5.29 | 4.37 | 3.141 | 1030 | √ | 7.193 | 1371 | 7.760 | 19.670 | -0.930 |
| *Loris tardigradus* | 15.0 | 1 | M | AMNH 34256(L) | - | 3.29 | 1.49 | 1.590 | 264 | √ | 5.134 | 175 | 3.890 | 8.670 | -0.801 |
| *Loris tardigradus* | 15.0 | 1 | M | AMNH 34256(R) | - | 3.15 | 1.57 | 1.598 | 264 | √ | 5.146 | 177 | 3.910 | 8.700 | -0.800 |
| *Loris tardigradus* | 18.0 | 1 | ? | AMNH 150038 | - | 3.34 | 1.77 | 1.777 | 266.5 | √ | 5.383 | 224 | 4.030 | 9.480 | -0.855 |
| *Loris tardigradus* | 18.0 | 1 | F | AMNH 165931 | - | 3.24 | 1.87 | 1.802 | 269 | √ | 5.415 | 232 | 3.610 | 8.760 | -0.886 |
| *Nycticebus coucang* | 20.5 | 2 | M | AMNH 16951 | - | 2.71 | 3.84 | 2.342 | 679 | √ | 6.133 | 475 | 4.350 | 11.870 | -1.004 |
| *Nycticebus javanicus* | 20.5 | 2 | F | AMNH 102027 | - | 5.03 | 2.58 | 2.563 | NA | - | 6.426 | 637 | 4.620 | 12.500 | -0.995 |
| *Nycticebus coucang* | 20.5 | 2 | ? | AMNH 90381 | - | 3.96 | 2.68 | 2.362 | NA | - | 6.159 | 488 | 4.210 | 11.780 | -1.029 |
| *Perodicticus potto* | 20.5 | 2 | F | AMNH 269907 | - | 5.03 | 3.48 | 2.862 | 1049 | √ | 6.823 | 948 | 4.710 | 13.420 | -1.047 |
| *Perodicticus potto* | 20.5 | 2 | F | AMNH 269851 | - | 4.5 | 3.3 | 2.698 | 1049 | √ | 6.605 | 762 | 4.720 | 12.720 | -0.991 |
| *Perodicticus potto* | 20.5 | 2 | M | AMNH 184597 | - | 4.75 | 3.71 | 2.869 | 1057 | √ | 6.832 | 956 | 4.520 | 12.630 | -1.028 |
| *Perodicticus potto* | 20.5 | 2 | M | AMNH 52685 | - | 5.29 | 3.76 | 2.990 | 1057 | √ | 6.993 | 1123 | 5.920 | 15.320 | -0.951 |
| *Arctocebus calabarensis* | 18.0 | 1 | F | AMNH 212954 | - | 3.21 | 1.8 | 1.754 | 306 | √ | 5.352 | 218 | 3.160 | 8.460 | -0.985 |
| *Arctocebus calabarensis* | 18.0 | 1 | M | AMNH 207949 | - | 2.96 | 1.92 | 1.738 | 312 | √ | 5.330 | 213 | 3.140 | 7.770 | -0.906 |
| *Hylobates lar* | 41.556 | 3 | ? | AMNH 119601 | - | 11.31 | 8.08 | 4.515 | NA | - | 9.017 | 8497 | 8.430 | 27.760 | -1.192 |
| *Symphalangus syndactylus* | 41.556 | 3 | F | AMNH 106583 | - | 9.37 | 6.79 | 4.153 | 10700 | √ | 8.536 | 5255 | 8.680 | 25.250 | -1.068 |
| *Hylobates sp. [hoolock?]* | 41.556 | 3 | M | AMNH 146725 | - | 20.95 | 5.42 | 4.732 | NA | - | 9.305 | 11336 | 8.010 | 20.950 | -0.961 |
| *Presbytis melalophos* | 63.25 | 3 | M | AMNH 106599 | - | 9.9 | 6.62 | 4.183 | 6590 | √ | 8.576 | 5466 | 12.250 | 30.490 | -0.912 |
| *Trachypithecus cristata* | 77.9 | 2 | M | MCZ 37670 | - | 10.21 | 6.71 | 4.227 | 6610 | √ | 8.635 | 5797 | 9.160 | 24.050 | -0.965 |
| *Trachypithecus obscura* | 41.556 | 3 | M | AMNH 112977 | - | 9.78 | 7.06 | 4.235 | 8167 | √ | 8.645 | 5857 | 11.420 | 30.030 | -0.967 |
| *Theropithecus gelada* | 187.5 | 5 | NA | SBU_Th5 | - | 13.36 | 8.52 | 4.735 | NA | - | 9.309 | 11373 | 12.430 | 39.160 | -1.148 |
| *Theropithecus gelada* | ~40 | 6 | NA | AMNH 201008 | - | 14.56 | 9.74 | 4.955 | NA | - | 9.600 | 15227 | 14.860 | 44.530 | -1.097 |
| *Nasalis larvatus* | 44.883 | 4 | M | MCZ 37327 | - | 14.86 | 9.75 | 4.976 | 20400 | √ | 9.629 | 15666 | 15.010 | 41.770 | -1.023 |
| *Nasalis larvatus* | 44.883 | 4 | M | MCZ 37328 | - | 14.48 | 11.58 | 5.122 | 20400 | √ | 9.823 | 19019 | 16.410 | 44.310 | -0.993 |
| *Nasalis larvatus* | 44.883 | 4 | M | MCZ 41557 | - | 15.17 | 10.46 | 5.067 | 20400 | √ | 9.750 | 17676 | 15.450 | 43.120 | -1.026 |
| *Chlorocebus aethiops* | 39.0 | 2 | NA | SBU OCr | - | 8.92 | 6.24 | 4.019 | NA | - | 8.359 | 4400 | 9.720 | 27.630 | -1.045 |
| *Chlorocebus aethiops* | 39.0 | 2 | NA | SBU OCr07 | - | 8.33 | 6.32 | 3.964 | NA | - | 8.285 | 4087 | 10.870 | 27.880 | -0.942 |
| *Macaca nigra* | 39.0 | 2 | NA | SBU OCn01 | - | 10.12 | 6.99 | 4.259 | NA | - | 8.677 | 6049 | 12.010 | 30.390 | -0.928 |
| *Pan troglodytes troglodytes* | 44.883 | 4 | F | AMNH 167343 | - | 25.04 | 15.7 | 5.974 | 45800 | √ | 10.954 | 58940 | 17.680 | 58.770 | -1.201 |
| *Pan troglodytes verus* | 44.883 | 4 | F | AMNH 89354 | - | 22.89 | 15.5 | 5.872 | 41600 | √ | 10.818 | 51436 | 15.050 | 58.530 | -1.358 |
| *Pan troglodytes verus* | 44.883 | 4 | F | AMNH 89426 | - | 22.42 | 14.53 | 5.786 | 41600 | √ | 10.704 | 45925 | 15.920 | 52.790 | -1.199 |
| *Gorilla gorilla gorilla* | 187.5 | 5 | F | MCZ 17684 | - | 23.88 | 21.63 | 6.247 | 71500 | √ | 11.316 | 84680 | 14.150 | 67.090 | -1.556 |
| *Gorilla gorilla gorilla* | 187.5 | 5 | M | MCZ 20038 | - | 29.88 | 23.95 | 6.573 | 170400 | √ | 11.749 | 130538 | 19.630 | 85.640 | -1.473 |
| *Gorilla gorilla gorilla* | 187.5 | 5 | M | MCZ 29048 | - | 30.83 | 23.91 | 6.603 | 170400 | √ | 11.788 | 135774 | 19.630 | 88.570 | -1.507 |
| *Pongo pygmaeus* | 187.5 | 5 | F | MCZ 37363 | - | 19.51 | 17.11 | 5.811 | 35700 | √ | 10.737 | 47438 | 15.920 | 49.970 | -1.144 |
| *Pongo pygmaeus* | 187.5 | 5 | M | MCZ 37362 | - | 23.37 | 20.54 | 6.174 | 78200 | √ | 11.219 | 76830 | 15.870 | 60.810 | -1.343 |
| *Pongo pygmaeus* | 187.5 | 5 | F | MCZ 37365 | - | 20.29 | 18.29 | 5.916 | 35700 | √ | 10.877 | 54598 | 14.640 | 53.720 | -1.300 |
| *Procolobus badius* | ~40 | 6 | M | AMNH 52303 | - | 11.24 | 8.16 | 4.519 | 8692 | √ | 9.022 | 8539 | 14.830 | 35.790 | -0.881 |
| *Procolobus badius* | ~40 | 6 | NA | ED 4651 | - | 9.83 | 7.23 | 4.264 | NA | - | 8.683 | 6086 | 11.110 | 28.790 | -0.952 |
| *Pygathrix nemaeus* | ~40 | 6 | M | AMNH 87225 | - | 14.3 | 8.76 | 4.830 | 10910 | √ | 9.436 | 12915 | 15.510 | 38.590 | -0.912 |
| *Papio ursinus* | ~40 | 6 | M | AMNH 80774 | - | 15.92 | 12.65 | 5.305 | 29858 | √ | 10.066 | 24255 | 15.430 | 48.720 | -1.150 |
| *Macaca fascicularis* | 78.0 | 2 | M | MCZ 35613 | - | 9.14 | 6.19 | 4.036 | 5360 | √ | 8.381 | 4497 | 8.750 | 26.190 | -1.096 |
| *Macaca fascicularis* | 78.0 | 2 | M | MCZ 35656 | - | 8.75 | 6.49 | 4.039 | 5360 | √ | 8.386 | 4519 | 10.720 | 26.330 | -0.899 |
| *Macaca fascicularis* | 78.0 | 2 | M | MCZ 35673 | - | 8.59 | 5.87 | 3.920 | 5360 | √ | 8.228 | 3859 | 9.680 | 25.440 | -0.966 |
| *Erythrocebus patas pyr.* | ~40 | 6 | F | AMNH 34709 | - | 10 | 6.58 | 4.187 | 6500 | √ | 8.581 | 5495 | 11.940 | 31.980 | -0.985 |
| *Lophocebus albigena* | ~40 | 6 | M | AMNH 52603 | - | 11.2 | 7.96 | 4.490 | 8092 | √ | 8.984 | 8223 | 12.760 | 35.690 | -1.029 |
| *Allenopithecus nigroviridis* | 36.0 | 1 | NA | RS pers Coll. | - | 8.27 | 6.47 | 3.980 | NA | - | 8.307 | 4176 | 11.140 | 29.050 | -0.958 |
| *Chlorocebus cynosuros* | ~40 | 6 | M | AMNH 80787 | - | 9.72 | 7.12 | 4.237 | 5950 | √ | 8.648 | 5875 | 12.040 | 29.500 | -0.896 |
| *Colobus guereza* | ~40 | 6 | M | AMNH 27711 | - | 12.89 | 9.64 | 4.822 | 9022 | √ | 9.425 | 12777 | 12.220 | 37.700 | -1.127 |
| *Callicebus donacophilus* | 20.5 | 2 | F | AMNH 211490 | - | 5.75 | 4.1 | 3.160 | 919 | √ | 7.219 | 1407 | 7.350 | 17.280 | -0.855 |
| *Callicebus moloch* | 20.5 | 2 | M | AMNH 94977 | - | 6.04 | 4.04 | 3.195 | 1020 | √ | 7.264 | 1473 | 7.730 | 18.760 | -0.887 |
| *Callicebus cupreus* | 20.5 | 2 | F | AMNH 130361 | - | 5.69 | 4.1 | 3.150 | 1120 | √ | 7.205 | 1387 | 6.270 | 17.170 | -1.007 |
| *Aotus infulatus* | 20.5 | 2 | F | AMNH 94992 | - | 5.77 | 4.15 | 3.176 | 1240 | √ | 7.239 | 1436 | 7.190 | 17.190 | -0.872 |
| *Aotus nancymaae [trivirgatus]* | 20.5 | 2 | M | AMNH 239851 | - | 6.09 | 3.98 | 3.188 | 780 | √ | 7.255 | 1460 | 7.290 | 18.360 | -0.924 |
| *Aotus azarae [trivirgatus]* | 20.5 | 2 | F | AMNH 187963 | - | 5.4 | 3.53 | 2.948 | 736 | √ | 6.937 | 1061 | 7.660 | 18.930 | -0.905 |
| *Saimiri sciureus* | 20.5 | 2 | M | AMNH 188090 | - | 5.02 | 3.4 | 2.837 | 779 | √ | 6.790 | 916 | 7.040 | 16.090 | -0.827 |
| *Saimiri boliviensis* | 20.5 | 2 | F | AMNH 211650 | - | 5.23 | 3.41 | 2.881 | 711 | √ | 6.848 | 971 | 6.850 | 15.500 | -0.817 |
| *Saimiri boliviensis* | 20.5 | 2 | F | AMNH 211613 | - | 5.35 | 3.6 | 2.958 | 711 | √ | 6.950 | 1076 | 7.150 | 16.680 | -0.847 |
| *Cebus apella* | 25.0 | 2 | F | AMNH 133608 | - | 8.18 | 5.06 | 3.723 | 2520 | √ | 7.966 | 2970 | 10.660 | 24.200 | -0.820 |
| *Cebus apella* | 25.0 | 2 | F | AMNH 133647 | - | 7.68 | 5.91 | 3.815 | 2520 | √ | 8.088 | 3356 | 9.410 | 23.370 | -0.910 |
| *Cebus apella* | 25.0 | 2 | F | AMNH 133674 | - | 8.33 | 5.35 | 3.797 | 2520 | √ | 8.064 | 3276 | 10.410 | 23.480 | -0.813 |
| *Leontopithecus rosalia* | 26.523 | 3 | ? | AMNH 185347 | - | 4.29 | 2.82 | 2.493 | 609 | √ | 6.333 | 580 | 6.060 | 14.710 | -0.887 |
| *Callithrix j. penicillata* | 26.523 | 3 | F | AMNH 133702 | - | 3.42 | 2.2 | 2.018 | 307 | √ | 5.703 | 309 | 4.520 | 10.720 | -0.864 |
| *Saguinus midas* | 26.523 | 3 | F | AMNH 266481 | - | 5.31 | 3.33 | 2.873 | 575 | √ | 6.837 | 960 | 6.590 | 15.370 | -0.847 |
| *Saguinus mystax (fuscicollis)* | 26.523 | 3 | M | AMNH 188177 | - | 3.33 | 2.6 | 2.158 | 343 | √ | 5.889 | 372 | 4.990 | 11.420 | -0.828 |
| *Saguinus mystax* | 26.523 | 3 | M | AMNH 188174 | - | 3.52 | 2.49 | 2.171 | 343 | √ | 5.905 | 378 | 4.810 | 11.280 | -0.852 |
| *Pithecia pithecia* | 35.0 | 2 | M | AMNH 149149 | - | 7.07 | 5.24 | 3.612 | 1940 | √ | 7.819 | 2563 | 8.260 | 22.160 | -0.987 |
| *Pithecia monachus* | 35.0 | 2 | NA | AMNH 187978 | - | 8.59 | 6.14 | 3.965 | NA | - | 8.288 | 4097 | 9.580 | 25.390 | -0.975 |
| *Pithecia hirsuta* | 35.0 | 2 | NA | AMNH 202373 | - | 7.34 | 5.21 | 3.644 | NA | - | 7.861 | 2674 | 8.140 | 22.220 | -1.004 |
| *Cacajao calvus* | 35.0 | 2 | NA | AMNH 201122 | - | 9.86 | 6.25 | 4.121 | NA | - | 8.494 | 5037 | 12.140 | 29.790 | -0.898 |
| *Cacajao calvus* | 35.0 | 2 | M | AMNH 70192 | - | 9.78 | 6.44 | 4.143 | 3450 | √ | 8.523 | 5185 | 12.580 | 30.160 | -0.874 |
| *Cacajao* sp*.* | 35.0 | 2 | NA | SBU NCj1 | - | 9.94 | 6.05 | 4.097 | NA | - | 8.462 | 4876 | 12.570 | 28.000 | -0.801 |
| *Chiropotes satanas irrorata* | 35.0 | 2 | M | AMNH 95760 | - | 9.76 | 6.2 | 4.103 | NA | - | 8.470 | 4916 | 10.120 | 26.020 | -0.944 |
| *Chiropotes satanas satanas* | 35.0 | 2 | M | AMNH 96123 | - | 7.79 | 6.14 | 3.868 | 3010 | √ | 8.158 | 3598 | 9.640 | 24.130 | -0.918 |
| *Chiropotes* sp. | 35.0 | 2 | NA | SBU NCh2 | - | 8.61 | 6.36 | 4.003 | NA | - | 8.337 | 4306 | 10.550 | 26.640 | -0.926 |
| *Callimico goeldii* | 20.5 | 2 | NA | AMNH 183289 | - | 4.58 | 3.11 | 2.656 | 516 | √ | 6.550 | 721 | 5.160 | 13.170 | -0.937 |
| *Callimico goeldii* | 30.0 | 2 | NA | SBU NCm01 | - | 4.92 | 3.25 | 2.772 | 516 | √ | 6.703 | 840 | 5.890 | 13.750 | -0.848 |
| *Callithrix pygmaea* | 18.0 | 1 | F | AMNH 244101 | - | 2.27 | 1.44 | 1.184 | 122 | √ | 4.596 | 102 | 2.960 | 6.960 | -0.855 |
| *Callithrix pygmaea* | 18.0 | 1 | NA | AMNH 244365 | - | 2.45 | 1.64 | 1.391 | NA | - | 4.870 | 134 | 3.040 | 7.120 | -0.851 |
| *Ateles belzebuth* | 39.0 | 2 | F | AMNH 259 | - | 12.59 | 8.47 | 4.669 | 7850 | √ | 9.222 | 10430 | 14.530 | 34.940 | -0.877 |
| *Ateles fusciceps* | 39.0 | 2 | M | AMNH 188140 | - | 13.47 | 9.49 | 4.851 | 8890 | √ | 9.463 | 13267 | 13.610 | 35.080 | -0.947 |
| *Ateles geoffroyi* | 39.0 | 2 | NA | AMNH 172985 | - | 13.14 | 10.69 | 4.945 | NA | - | 9.588 | 15035 | 14.250 | 35.270 | -0.906 |
| *Alouatta caraya* | 39.0 | 2 | M | AMNH 211510 | - | 10.97 | 7.61 | 4.425 | 6420 | √ | 8.897 | 7536 | 11.440 | 33.940 | -1.087 |
| *Alouatta caraya* | 39.0 | 2 | M | AMNH 211525 | - | 11.07 | 7.37 | 4.402 | 6420 | √ | 8.867 | 7310 | 10.230 | 31.660 | -1.130 |
| *Alouatta caraya* | 39.0 | 2 | M | AMNH 211521 | - | 9.37 | 5.93 | 4.018 | 6420 | √ | 8.357 | 4390 | 10.040 | 30.850 | -1.123 |
| *Alouatta* sp. | 39.0 | 2 | NA | SBU NAl17 | - | 10.57 | 8.46 | 4.493 | NA | - | 8.988 | 8256 | 11.540 | 33.010 | -1.051 |
| *Lagothrix lagotricha* | 39.0 | 2 | F | AMNH 188156(L) | - | 10.26 | 6.55 | 4.208 | 7090 | √ | 8.609 | 5651 | 11.860 | 29.920 | -0.925 |
| *Lagothrix lagotricha* | 39.0 | 2 | F | AMNH 188156(R) | - | 10.66 | 6.26 | 4.201 | 7090 | √ | 8.600 | 5598 | 11.730 | 30.670 | -0.961 |
| *Proteopithecus sylviae* | 15.0 | 1 | NA | DPC 24776 | - | 2.17 | 3.31 | 1.972 | NA | - | 5.641 | 290 | 5.490 | 12.530 | -0.825 |
| Parapithecidae | 30.839 | 3 | NA | DPC 8810 | - | 2.66 | 5.64 | 2.708 | NA | - | 6.619 | 772 | 7.820 | 20.730 | -0.975 |
| Parapithecidae | 28.645 | 3 | NA | DPC 15679 | - | 3.72 | 5.61 | 3.038 | NA | - | 7.057 | 1197 | 7.850 | 21.790 | -1.021 |
| Parapithecidae | 28.645 | 3 | NA | DPC 2381 | - | 2.87 | 5.85 | 2.821 | NA | - | 6.768 | 896 | 7.810 | 21.320 | -1.004 |
| Parapithecidae | 21.924 | 3 | NA | DPC 20576 | - | 2.14 | 4.01 | 2.150 | NA | - | 5.877 | 368 | 6.530 | 15.890 | -0.889 |
| Parapithecidae | 30.839 | 3 | NA | DPC 1003 | - | 2.32 | 5.13 | 2.477 | NA | - | 6.311 | 568 | 9.350 | 23.390 | -0.917 |
| *Eosimias* | 14.143 | 3 | NA | IVPP 12313 | - | 2.3 | 1.63 | 1.321 | NA | - | 4.778 | 123 | 3.990 | 8.410 | -0.746 |
| *Eosimias* | 14.143 | 3 | NA | IVPP 12280 | - | 2.16 | 1.53 | 1.195 | NA | - | 4.611 | 104 | 4.040 | 8.110 | -0.697 |
| Eosimiid | 4.200 | 3 | NA | IVPP 11847 | - | 1.02 | 0.72 | -0.309 | NA | - | 2.614 | 14 | 2.090 | 4.160 | -0.688 |
| *Eosimias* | 7.081 | 3 | NA | IVPP 11851 | - | 1.95 | 1.04 | 0.707 | NA | - | 3.962 | 54 | 3.090 | 6.740 | -0.780 |
| Eosimiid | 3.323 | 3 | NA | IVPP 11848 | - | 1.29 | 0.99 | 0.245 | NA | - | 3.348 | 29 | 2.220 | 4.530 | -0.713 |
| *Tupaia belangeri* | 15.0 | 1 | NA | AMNH 113135 | - | 2.42 | 1.83 | 1.488 | NA | - | 4.999 | 153 | 3.270 | 8.950 | -1.007 |
| *Tupaia* sp. | 15.0 | 1 | NA | AMNH 215178 | - | 2.36 | 1.84 | 1.468 | NA | - | 4.973 | 149 | 3.270 | 8.500 | -0.955 |
| *Tupaia* sp. | 15.0 | 1 | NA | AMNH 215179 | - | 2.32 | 1.67 | 1.354 | NA | - | 4.822 | 128 | 3.030 | 8.040 | -0.976 |
| *Tupaia* sp. | 15.0 | 1 | NA | AMNH 215175 | - | 2.27 | 1.75 | 1.379 | NA | - | 4.855 | 132 | 3.450 | 8.740 | -0.930 |
| *Ptilocercus lowii* | 20.5 | 2 | NA | USNM 488055 | - | 1.36 | 1.19 | 0.481 | NA | - | 3.663 | 40 | 1.960 | 5.100 | -0.956 |
| *Ptilocercus lowii* | 20.5 | 2 | NA | USNM 488058 | - | 1.37 | 1.12 | 0.428 | NA | - | 3.592 | 37 | 1.850 | 5.000 | -0.994 |
| *Ptilocercus lowii* | 20.5 | 2 | NA | USNM 481106 | - | 1.34 | 1.26 | 0.524 | NA | - | 3.719 | 42 | 1.900 | 5.020 | -0.972 |
| *Cynocephalus volans* | 20.5 | 2 | NA | AMNH 207001 | - | 5.1 | 4.4 | 3.111 | NA | - | 7.153 | 1318 | 5.740 | 14.800 | -0.947 |
| *Cynocephalus volans* | 20.5 | 2 | NA | UNSM 15502 | - | 4.45 | 3.91 | 2.856 | NA | - | 6.815 | 940 | 5.020 | 13.030 | -0.954 |
| *Plesiadapis cookei* | 18.0 | 1 | NA | UM 87990 | - | 6.61 | 5.06 | 3.510 | NA | - | 7.683 | 2238 | 5.570 | 17.700 | -1.156 |
| *Nannodectes gidleyi* | Na | Na | NA | AMNH 17379 | - | 3.13 | 2.79 | 2.167 | NA | - | 5.900 | 376 | 3.070 | 9.840 | -1.165 |
| *Carpolestes simpsoni* | 10.0 | 1 | NA | UM 101963 | - | 2.35 | 2.11 | 1.601 | NA | - | 5.149 | 178 | 2.650 | 6.690 | -0.926 |
| Paromomyidae sp. indet. | 10.0 | 1 | NA | USNM 442240 | - | 2.3 | 1.87 | 1.459 | NA | - | 4.960 | 147 | 2.410 | 7.260 | -1.103 |
| *Dryomomys szalayi* | 8.0 | 1 | NA | UM 41870 | - | 0.83 | 0.73 | -0.501 | NA | - | 2.359 | 11 | 0.830 | 2.610 | -1.146 |
| *Paleopropithecus* cf. *ingens* | Na | Na | NA | DPC 17214A | - | 11.8 | 8.04 | 4.553 | NA | - | 9.067 | 8931 | 5.23 | 25.56 | -1.587 |
| *Paleopropithecus* cf. *ingens* | Na | Na | NA | DPC 17214A | - | 12.62 | 9.46 | 4.782 | NA | - | 9.372 | 12116 | 5.65 | 27.49 | -1.582 |
| *Paleopropithecus* sp*.* | Na | Na | NA | DPC 17164 | - | 13.21 | 8.71 | 4.745 | NA | - | 9.323 | 11537 | 7.27 | 28.37 | -1.362 |
| *Babakotia radofilai* | Na | Na | NA | DPC 11824 | - | 11.66 | 6.46 | 4.322 | NA | - | 8.761 | 6575 | 11.92 | 28.75 | -0.880 |
| *Babakotia radofilai* | Na | Na | NA | DPC 11824 | - | 11.99 | 6.22 | 4.312 | NA | - | 8.747 | 6488 | 12.29 | 28.82 | -0.852 |
| *Babakotia radofilai* | Na | Na | NA | DPC 11818 | - | 9.54 | 5.83 | 4.019 | NA | - | 8.358 | 4396 | 12 | 27.52 | -0.830 |
| *Mesopropithecus dolichobrachion* | Na | Na | NA | DPC 6833 | - | 8.41 | 5.23 | 3.784 | NA | - | 8.046 | 3219 | 7.69 | 25.07 | -1.182 |
| *Pachylemur insignis* | Na | Na | NA | DPC 11822 | - | 12.33 | 7.56 | 4.535 | NA | - | 9.043 | 8724 | 9.04 | 31.05 | -1.234 |
| *Archaeolemur* cf. *edwardsi*. | Na | Na | NA | DPC 9106 R | - | 14.29 | 9.82 | 4.944 | NA | - | 9.586 | 15016 | 12.69 | 38.24 | -1.103 |
| *Archaeolemur* cf. *edwardsi* | Na | Na | NA | DPC 9106 L | - | 14.42 | 10.02 | 4.973 | NA | - | 9.625 | 15609 | 12.65 | 38.4 | -1.110 |
| *Archaeolemur* sp. | Na | Na | NA | DPC 12879 R | - | 13.41 | 10.94 | 4.988 | NA | - | 9.645 | 15928 | 11.71 | 35.12 | -1.098 |
| *Archaeolemur* sp. | Na | Na | NA | DPC 12879 L | - | 14.05 | 10.42 | 4.986 | NA | - | 9.643 | 15884 | 11.77 | 37.71 | -1.164 |
| *Archaeolemur* majori | Na | Na | NA | DPC 18740 | - | 14.42 | 10.12 | 4.983 | NA | - | 9.638 | 15817 | 11.98 | 36.8 | -1.122 |
| *Megaladapis* cf. *madagascariensis* | Na | Na | NA | DPC 18936 | - | 26.24 | 13.68 | 5.883 | NA | - | 10.833 | 52239 | 19.77 | 59.01 | -1.094 |
| *Megaladapis madagascariensis* | Na | Na | NA | DPC 13733 | - | 21.56 | 10.92 | 5.461 | NA | - | 10.273 | 29843 | 17.07 | 45.86 | -0.988 |
| *Megaladapis* cf. *madagascariensis*/grandidieri | Na | Na | NA | DPC 9089 | - | 20.02 | 12.11 | 5.491 | NA | - | 10.312 | 31028 | 16.89 | 51.68 | -1.118 |

Abbreviations: antilog(bm), antilog of value in “est ln(bm)” field and corrected through multiplication by Quasi-Maximum Likelihood Estimator1.0308 (Delson et al. 2000); BM, body mass in grams from literature (*, Walkers Mammals of the World; +, Primates in Perspective; otherwise mass value comes from Smith & Jungers 1997); bm reg, specimens used in regression of body mass on CA; CA, cuboid area =(CW*CD); CD, cuboid depth (in mm); CE, Calcaneal elongation=(DL/TL); CW, cuboid width (in millimeters - mm); DL, distal length of calcaneus as measured from distal margin of ectal facet (in mm); ln, natural logarithm; M, male; ML, meter level in Bighorn Basin (see Gebo et al., 1991); F, female; TL, total proximodistal length of calcaneus (in mm); Res, scanning resolution in microns; Scan, scanning facility/machine model: **1)** SBU, Scanco uCT40**, 2)** SBU Scanco vivaCT75, **3)** AMNH Phoenix v / tome / x, **4)** OSU GE Locus SP, **5)** Medical CT, **6)** Bruekman Structured Light Scanner.

**Table S2.** Ancestral state reconstructions for Tree 1. Tree 1 is MRP supertree that incorporates the strict consensus of the most parsimonious trees found by analysis of the modified matrix of Boyer and Seiffert (in press), generated as described in text. Trees 1 and 2 differ in branch lengths (Tree 1 with a longer base). Trees 1-2 differ from 3-4 in topology. See Fig. 8A-B for nodes of interest and specific differences between trees.

| Clade | Estimated ln body mass | | | ln(distal length/total length) | | |
| --- | --- | --- | --- | --- | --- | --- |
|  |  |  |  |  |  |  |
|  | Phylogenetic scaling parameter δ, directional | | | Phylogenetic scaling parameter κ | | |
|  | **Mean** | **L 95%HPD** | **U 95% HPD** | **Mean** | **L 95% HPD** | **U 95% HPD** |
| Euarchonta root | 2.517 | 0.792 | 4.222 | -0.9730 | -1.1558 | -0.7867 |
| Scandentia | 3.017 | 0.400 | 5.594 | -0.9704 | -1.2800 | -0.6573 |
| Primatomorpha | 3.393 | 1.608 | 5.198 | -0.9741 | -1.1566 | -0.7890 |
| Euprimateforms (Plesiadapoidea + Primates) | 4.245 | 3.025 | 5.475 | -0.9506 | -1.0857 | -0.8133 |
| Plesiadapoidea | 5.760 | 5.096 | 6.434 | -1.0722 | -1.1551 | -0.9872 |
| Paromomyidae + Euprimateforms (1) | 4.055 | 2.779 | 5.320 | -0.9670 | -1.1070 | -0.8303 |
| Micromomyidae + 1 | 3.841 | 2.498 | 5.206 | -0.9763 | -1.1234 | -0.8305 |
| **Crown Primates (=Euprimates)** | 4.336 | 3.105 | 5.579 | -0.9228 | -1.0604 | -0.7822 |
| **Crown Haplorhini** | 4.294 | 3.093 | 5.512 | -0.7629 | -0.9073 | -0.6142 |
| Crown Anthropoidea | 5.978 | 4.994 | 6.983 | -0.8637 | -1.0007 | -0.7297 |
| *Crown* *Platyrrhini* | 6.946 | 5.931 | 7.957 | -0.9006 | -1.0398 | -0.7647 |
| Callitrichidae | 6.212 | 5.337 | 7.082 | -0.8750 | -0.9968 | -0.7493 |
| Cebidae | 6.876 | 5.853 | 7.906 | -0.8757 | -1.0225 | -0.7336 |
| Ceboidea | 6.774 | 5.851 | 7.685 | -0.8874 | -1.0128 | -0.7622 |
| Atelidae | 8.064 | 7.018 | 9.100 | -0.9625 | -1.1097 | -0.8136 |
| Pitheciidae | 7.158 | 6.074 | 8.263 | -0.9023 | -1.0560 | -0.7500 |
| *Crown* *Catarrhini* | 8.374 | 7.117 | 9.643 | -1.0449 | -1.2199 | -0.8680 |
| Cercopithecoidea | 8.660 | 7.811 | 9.515 | -0.9998 | -1.1224 | -0.8791 |
| Hominoidea | 9.468 | 8.463 | 10.491 | -1.1637 | -1.3081 | -1.0184 |
| Hominidae | 9.806 | 8.817 | 10.795 | -1.2035 | -1.3445 | -1.0609 |
| Homininae | 10.676 | 9.817 | 11.533 | -1.3191 | -1.4427 | -1.1921 |
| Proteopithecidae + Parapithecidae (2) | 5.769 | 5.262 | 6.281 | -0.8483 | -0.9193 | -0.7796 |
| Crown Anthropoidea + 2 (3) | 5.784 | 4.888 | 6.683 | -0.8637 | -1.0007 | -0.7297 |
| Eosimiidae + 3 | 4.571 | 4.006 | 5.136 | -0.7544 | -0.8300 | -0.6790 |
| *Tarsiiformes* | 4.372 | 3.703 | 5.034 | -0.6502 | -0.7333 | -0.5668 |
| Microchoerinae + *Tarsius* (4) | 5.012 | 3.867 | 6.166 | -0.4576 | -0.6098 | -0.3042 |
| Anaptomorphinae + 4 (5) | 4.351 | 3.711 | 5.006 | -0.6079 | -0.6903 | -0.5253 |
| Washakiini + 5 | 4.368 | 3.708 | 5.039 | -0.6284 | -0.7122 | -0.5446 |
| Omomyinae | 5.688 | 5.052 | 6.332 | -0.6619 | -0.7451 | -0.5787 |
| **Crown Strepsirrhini** | 5.744 | 4.606 | 6.859 | -0.8529 | -0.9937 | -0.7128 |
| *Crown Lemuriformes* | 6.055 | 4.551 | 7.601 | -0.8631 | -1.0605 | -0.6676 |
| Cheirogaleidae | 5.744 | 4.493 | 7.001 | -0.7404 | -0.9159 | -0.5671 |
| Lepilemur + Cheirogaleidae (6) | 6.422 | 5.186 | 7.661 | -0.8277 | -0.9946 | -0.6628 |
| *Microcebus* + *Mirza* (7) | 5.141 | 3.99 | 6.220 | -0.6241 | -0.7895 | -0.4595 |
| Crown Indriidae (8) | 7.609 | 6.511 | 8.707 | -0.9373 | -1.0918 | -0.7824 |
| Paleopropithecidae + 8 (9) | 7.561 | 6.429 | 8.691 | -0.9782 | -1.1320 | -0.8225 |
| Archaeolemuridae + 9 (10) | 7.340 | 6.147 | 8.562 | -0.9460 | -1.1093 | -0.7833 |
| Crown Lemuridae (11) | 7.560 | 6.406 | 8.701 | -0.9195 | -1.0790 | -0.7602 |
| *Megaladapis* + 11 (12) | 7.505 | 6.234 | 8.750 | -0.9214 | -1.0909 | -0.7507 |
| 6 + 10 + 12 | 6.873 | 5.655 | 8.080 | -0.8887 | -1.0485 | -0.7312 |
| *Crown Lorisiformes* | 5.398 | 4.092 | 6.740 | -0.7681 | -0.9501 | -0.5862 |
| Crown Galagidae | 5.526 | 4.729 | 6.309 | -0.5901 | -0.7017 | -0.4792 |
| Galagidae - *Euoticus* | 5.474 | 4.441 | 6.481 | -0.4200 | -0.5652 | -0.2722 |
| *Galago* + *Otolemur* | 5.744 | 4.771 | 6.713 | -0.4086 | -0.5497 | -0.2685 |
| Crown Lorisidae | 5.402 | 4.043 | 6.743 | -0.7986 | -0.9885 | -0.6160 |
| *Anchomomys* + crown Strepsirrhini (13) | 5.737 | 4.721 | 6.755 | -0.8558 | -0.9822 | -0.7293 |
| Adapidae + 13 (14) | 5.839 | 4.902 | 6.788 | -0.8719 | -0.9864 | -0.7537 |
| Asiadapinae (15) | 5.342 | 4.917 | 5.768 | -0.9005 | -0.9561 | -0.8464 |
| Notharctinae (16) | 6.129 | 5.524 | 6.745 | -0.8522 | -0.9285 | -0.7759 |
| 14 + 15 +16 | 5.838 | 5.128 | 6.552 | -0.8660 | -0.9545 | -0.7781 |
| Adapidae | 7.818 | 7.286 | 8.358 | -1.1667 | -1.2387 | -1.0932 |

**Table S3.** Ancestral state reconstructions for Tree 2. See Fig. 8A-B for nodes of interest and specific differences between trees.

| **Clade** | Estimated ln body mass | | | ln(distal length/total length) | | |
| --- | --- | --- | --- | --- | --- | --- |
|  | Phylogenetic scaling parameter δ, directional | | | Phylogenetic scaling parameter κ | | |
|  | **Mean** | **L 95% HPD** | **U 95% HPD** | **Mean** | **L 95% HPD** | **U 95% HPD** |
| Euarchonta root | -2.1724 | -11.8909 | 4.1691 | -1.0122 | -1.1326 | -0.8934 |
| Scandentia | 0.8989 | -2.8412 | 4.4611 | -0.9990 | -1.1752 | -0.8273 |
| Primatomorpha | 1.8653 | -1.0316 | 4.5282 | -1.0181 | -1.1404 | -0.8934 |
| Euprimateforms (Plesiadapoidea + Primates) | 4.1422 | 2.7937 | 5.4149 | -0.9609 | -1.0624 | -0.8607 |
| Plesiadapoidea | 5.2096 | 4.207 | 6.198 | -1.0223 | -1.1185 | -0.9274 |
| Paromomyidae + Euprimateforms (1) | 3.5106 | 1.8581 | 5.063 | -1.0098 | -1.1171 | -0.9022 |
| Micromomyidae + 1 | 2.6189 | 0.4448 | 4.5669 | -1.0297 | -1.1442 | -0.9140 |
| Crown Primates (=Euprimates) | 4.3127 | 3.1047 | 5.4719 | -0.8713 | -0.9686 | -0.7732 |
| Crown Haplorhini | 4.125 | 2.944 | 5.2565 | -0.7835 | -0.8892 | -0.6812 |
| Crown Anthropoidea | 5.8742 | 4.8257 | 6.8994 | -0.8917 | -1.0362 | -0.7452 |
| Crown Platyrrhini | 6.7609 | 5.8095 | 7.697 | -0.9104 | -1.0534 | -0.7673 |
| Callitrichidae | 6.068 | 5.2782 | 6.838 | -0.8756 | -1.0033 | -0.7456 |
| Cebidae | 6.7227 | 5.8056 | 7.6572 | -0.8714 | -1.0134 | -0.7287 |
| Ceboidea | 6.591 | 5.7606 | 7.4289 | -0.8907 | -1.0181 | -0.7615 |
| Atelidae | 7.9769 | 7.0503 | 8.8944 | -0.9766 | -1.1186 | -0.8305 |
| Pitheciidae | 7.0027 | 5.9953 | 7.9891 | -0.9075 | -1.0551 | -0.7561 |
| Crown Catarrhini | 8.3151 | 7.1534 | 9.4897 | -1.0312 | -1.1997 | -0.8645 |
| Cercopithecoidea | 8.5914 | 7.8449 | 9.3405 | -1.0007 | -1.1360 | -0.8654 |
| Hominoidea | 9.4036 | 8.4907 | 10.3062 | -1.1431 | -1.2899 | -0.9979 |
| Hominidae | 9.7528 | 8.8805 | 10.6282 | -1.2089 | -1.3470 | -1.0672 |
| Homininae | 10.6506 | 9.9091 | 11.4007 | -1.3221 | -1.4469 | -1.1964 |
| Proteopithecidae + Parapithecidae (2) | 5.7355 | 5.2242 | 6.2619 | -0.8622 | -0.9527 | -0.7716 |
| Crown Anthropoidea + 2 (3) | 5.6924 | 4.7569 | 6.6319 | -0.8636 | -0.9929 | -0.7340 |
| Eosimiidae + 3 | 4.5236 | 3.8831 | 5.1611 | -0.7701 | -0.8697 | -0.6725 |
| Tarsiiformes | 4.0642 | 3.0787 | 5.0278 | -0.6980 | -0.7892 | -0.6077 |
| Microchoerinae + Tarsius (4) | 4.8278 | 3.5269 | 6.1388 | -0.4558 | -0.6017 | -0.3072 |
| Anaptomorphinae + 4 (5) | 4.0762 | 3.1223 | 5.0105 | -0.6037 | -0.7087 | -0.4944 |
| Washakiini + 5 | 4.0575 | 3.0483 | 5.0174 | -0.6466 | -0.7486 | -0.5427 |
| Omomyinae | 5.585 | 4.7343 | 6.4162 | -0.6733 | -0.7732 | -0.5707 |
| Crown Strepsirrhini | 4.8008 | 3.2205 | 6.3629 | -0.8409 | -0.9880 | -0.6915 |
| Crown Lemuriformes | 4.8008 | 3.2205 | 6.3629 | -0.8639 | -1.0444 | -0.6841 |
| Cheirogaleidae | 5.4428 | 4.2649 | 6.5834 | -0.7227 | -0.8859 | -0.5596 |
| Lepilemur + Cheirogaleidae (6) | 6.0555 | 4.8852 | 7.2227 | -0.8093 | -0.9681 | -0.6520 |
| Microcebus + Mirza (7) | 4.9639 | 3.9490 | 5.9891 | -0.6115 | -0.7613 | -0.4622 |
| Crown Indriidae (8) | 7.4412 | 6.4452 | 8.4251 | -0.9339 | -1.0810 | -0.7863 |
| Paleopropithecidae + 8 (9) | 7.3429 | 6.2983 | 8.3812 | -0.9923 | -1.1459 | -0.8411 |
| Archaeolemuridae + 9 (10) | 7.0696 | 5.9155 | 8.1855 | -0.9668 | -1.1244 | -0.8106 |
| Crown Lemuridae (11) | 7.3711 | 6.3265 | 8.4202 | -0.9313 | -1.0834 | -0.7767 |
| Megaladapis + 11 (12) | 7.2601 | 6.0826 | 8.4472 | -0.9403 | -1.1021 | -0.7779 |
| 6 + 10 + 12 | 6.4931 | 5.3217 | 7.6829 | -0.8983 | -1.0561 | -0.7424 |
| Crown Lorisiformes | 4.9723 | 3.6615 | 6.3154 | -0.7635 | -0.9291 | -0.5980 |
| Crown Galagidae | 5.3882 | 4.6494 | 6.1289 | -0.5797 | -0.6952 | -0.4632 |
| Galagidae - Euoticus | 5.3662 | 4.4542 | 6.2630 | -0.4144 | -0.5568 | -0.2737 |
| Galago + Otolemur | 5.6607 | 4.8045 | 6.5149 | -0.3996 | -0.5374 | -0.2639 |
| Crown Lorisidae | 4.9999 | 3.6581 | 6.3271 | -0.8173 | -0.9870 | -0.6465 |
| Anchomomys + crown Strepsirrhini (13) | 4.7925 | 3.2981 | 6.2492 | -0.8448 | -0.9762 | -0.7122 |
| Adapidae + 13 (14) | 4.9135 | 3.4756 | 6.3364 | -0.8861 | -1.0140 | -0.7599 |
| Asiadapinae (15) | 5.2204 | 4.6497 | 5.7953 | -0.8988 | -0.9754 | -0.8239 |
| Notharctinae (16) | 5.5136 | 4.5534 | 6.4268 | -0.8518 | -0.9437 | -0.7623 |
| 14 + 15 +16 | 4.8276 | 3.736 | 5.8997 | -0.8703 | -0.9627 | -0.7779 |
| Adapidae | 7.7585 | 7.174 | 8.3423 | -1.1460 | -1.2392 | -1.0490 |

**Table S4.** Ancestral state reconstructions for Tree 3. Tree 3 is the MRP supertree that incorporates the strict consensus of the most parsimonious trees found by analysis of the modified matrix of Boyer and Seiffert (in press), with adapiforms constrained to be stem or crown haplorhines. Otherwise generated as described in text. Trees 1-2 differ from 3-6 in topology. Trees 3-6 also differ in topology. See Fig. 8A-B for nodes of interest and specific differences between trees.

| **Clade** | Estimated ln body mass | | | ln(distal length/total length) | | |
| --- | --- | --- | --- | --- | --- | --- |
|  | Phylogenetic scaling parameter κ, directional | | | No phylogenetic scaling parameter | | |
|  | **Mean** | **L 95% HPD** | **U 95% HPD** | **Mean** | **L 95% HPD** | **U 95% HPD** |
| Euarchonta root | 2.301 | 0.8212 | 3.7886 | -0.9794 | -1.1611 | -0.7898 |
| Scandentia | 2.7714 | 0.9963 | 4.5001 | -0.9717 | -1.2861 | -0.6628 |
| Primatomorpha | 2.8766 | 1.4611 | 4.293 | -0.9811 | -1.1671 | -0.7975 |
| Euprimateforms (Plesiadapoidea + Primates) | 3.7454 | 2.6814 | 4.8156 | -0.9617 | -1.1018 | -0.8243 |
| Plesiadapoidea | 5.4464 | 4.7457 | 6.1522 | -1.073  -1.1567 | -1.1567 | -0.9892 |
| Paromomyidae + Euprimateforms (1) | 3.5079 | 2.4357 | 4.5774 | -0.9773 | -1.1192 | -0.8376 |
| Micromomyidae + 1 | 3.1667 | 2.0215 | 4.2758 | -0.9853 | -1.1321 | -0.8365 |
| Crown Primates (=Euprimates) | 3.8056 | 2.6952 | 4.935 | -0.9371 | -1.0818 | -0.7967 |
| Adapiformes + Haplorhini | 4.0839 | 3.1057 | 5.0516 | -0.8075 | -0.9317 | -0.6851 |
| Crown Haplorhini | 3.9975 | 3.0451 | 4.9737 | -0.7784 | -0.9026 | -0.6558 |
| Crown Anthropoidea | 5.7125 | 4.6872 | 6.7448 | -0.8646 | -1.0004 | -0.7323 |
| Crown Platyrrhini | 6.3247 | 5.2712 | 7.3551 | -0.901 | -1.0397 | -0.7624 |
| Callitrichidae | 5.8059 | 4.8819 | 6.7093 | -0.8749 | -0.9999 | -0.7522 |
| Cebidae | 6.5127 | 5.4898 | 7.5178 | -0.8762 | -1.0196 | -0.7305 |
| Ceboidea | 6.242 | 5.3023 | 7.1786 | -0.888 | -1.0135 | -0.7619 |
| Atelidae | 7.6278 | 6.6116 | 8.6507 | -0.9621 | -1.1098 | -0.8139 |
| Pitheciidae | 6.6694 | 5.5999 | 7.7498 | -0.9025 | -1.058 | -0.7505 |
| Crown Catarrhini | 7.5952 | 6.3758 | 8.7884 | -1.0453 | -1.2208 | -0.8663 |
| Cercopithecoidea | 8.0606 | 7.1005 | 9.0094 | -0.9996 | -1.1219 | -0.8783 |
| Hominoidea | 8.8531 | 7.8135 | 9.874 | -1.164 | -1.3098 | -1.0205 |
| Hominidae | 9.4635 | 8.4853 | 10.4492 | -1.203 | -1.3448 | -1.0618 |
| Homininae | 10.4693 | 9.6119 | 11.3407 | -1.3187 | -1.4423 | -1.1917 |
| Proteopithecidae + Parapithecidae (2) | 5.6906 | 5.0886 | 6.2897 | -0.8483 | -0.9187 | -0.7786 |
| Crown Anthropoidea + 2 (3) | 5.447 | 4.5299 | 6.3454 | -0.8512 | -0.9736 | -0.7303 |
| Eosimiidae + 3 | 4.4955 | 3.8271 | 5.1584 | -0.7553 | -0.8302 | -0.6794 |
| Tarsiiformes | 3.9169 | 3.1657 | 4.6848 | -0.6616 | -0.7555 | -0.5685 |
| Washakiini + Macrotarsiini +Tarsius (4) | 4.8444 | 4.0835 | 5.5948 | -0.6237 | -0.7088 | -0.5361 |
| Microchoerinae + 4 (5) | 4.5003 | 3.736 | 5.2586 | -0.6093 | -0.7003 | -0.517 |
| Anaptomorphinae + 5 (6) | 4.049 | 3.3246 | 4.77 | -0.6382 | -0.728 | -0.5522 |
| Arapahovius + 6 | 4.1926 | 3.4862 | 4.9043 | -0.6129 | -0.6987 | -0.5257 |
| Crown Strepsirrhini | 4.4811 | 2.9693 | 5.9356 | -0.8742 | -1.0961 | -0.6527 |
| Crown Lemuriformes | 5.1447 | 3.6171 | 6.6649 | -0.8792 | -1.1099 | -0.6471 |
| Cheirogaleidae | 5.1299 | 3.9468 | 6.3062 | -0.7412 | -0.9177 | -0.5663 |
| Lepilemur + Cheirogaleidae (7) | 5.6985 | 4.502 | 6.8628 | -0.8302 | -0.9952 | -0.6611 |
| Microcebus + Mirza (8) | 4.7771 | 3.704 | 5.8159 | -0.6251 | -0.7904 | -0.4599 |
| Crown Indriidae (9) | 7.2711 | 6.2187 | 8.3245 | -0.9382 | -1.0933 | -0.7831 |
| Paleopropithecidae + 9 (10) | 7.092 | 5.9807 | 8.2108 | -0.979 | -1.136 | -0.8244 |
| Archaeolemuridae + 10 (11) | 6.8561 | 5.6844 | 8.0269 | -0.9477 | -1.1109 | -0.7836 |
| Crown Lemuridae (12) | 7.1352 | 6.0165 | 8.2401 | -0.919 | -1.0781 | -0.7587 |
| Megaladapis + 12 (13) | 7.073 | 5.8618 | 8.2886 | -0.9226 | -1.0958 | -0.7521 |
| 7 + 11 + 13 | 6.1274 | 4.9144 | 7.339 | -0.8908 | -1.054 | -0.731 |
| Crown Lorisiformes | 4.6412 | 3.3647 | 5.8764 | -0.7725 | -0.9597 | -0.5898 |
| Crown Galagidae | 5.1274 | 4.2968 | 5.9375 | -0.591 | -0.7029 | -0.4798 |
| Galagidae - Euoticus | 5.0222 | 4.0265 | 6.0051 | -0.4205 | -0.568 | -0.2733 |
| Galago + Otolemur | 5.4359 | 4.5002 | 6.3956 | -0.4086 | -0.5495 | -0.2684 |
| Crown Lorisidae | 4.7101 | 3.454 | 5.9724 | -0.8025 | -0.9895 | -0.613 |
| Notharctinae (14) | 5.9973 | 5.3399 | 6.6566 | -0.8445 | -0.9241 | -0.7653 |
| Asiadapinae (15) | 5.1975 | 4.6889 | 5.7018 | -0.8998 | -0.9554 | -0.8448 |
| Adapidae (16) | 7.6767 | 7.0405 | 8.3 | -1.1677 | -1.2413 | -1.0953 |
| Adapidae + Notharctinae (17) | 5.6619 | 4.8698 | 6.4465 | -0.8519 | -0.9465 | -0.7558 |
| Anchomomyini + 17 (18) | 5.1573 | 4.3107 | 6.0041 | -0.8421 | -0.9459 | -0.7377 |
| Asiadapinae + 18 | 4.8415 | 3.9661 | 5.7243 | -0.8417 | -0.951 | -0.7319 |

**Table S5.** Ancestral state reconstructions for Tree 4. Tree 4 is the MRP supertree that incorporates the strict consensus of the most parsimonious trees found by analysis of the modified matrix of Boyer and Seiffert (in press) with *Tarsius* and Anthropoidea constrained to form a clade to the exclusion of omomyiforms and other non-anthropoid primates. Otherwise generated as described in text.

| **Clade** | Estimated ln body mass | | | Ln(distal length/total length) | | |
| --- | --- | --- | --- | --- | --- | --- |
|  | Phylogenetic scaling parameter κ, directional | | | No phylogenetic scaling parameter | | |
|  | **Mean** | **L 95% HPD** | **U 95% HPD** | **Mean** | **L 95% HPD** | **U 95% HPD** |
| Euarchonta root | 2.4884 | 1.0736 | 3.9197 | -0.9517 | -1.1438 | -0.7653 |
| Scandentia | 2.8749 | 1.0914 | 4.6310 | -0.9573 | -1.2787 | -0.6436 |
| Primatomorpha | 3.0344 | 1.6693 | 4.3799 | -0.9503 | -1.1355 | -0.7615 |
| Euprimateforms (Plesiadapoidea + Primates) | 3.9247 | 2.9583 | 4.9004 | -0.9193 | -1.0525 | -0.7826 |
| Paromomyidae + Euprimateforms (1) | 3.6707 | 2.6761 | 4.6737 | -0.9393 | -1.0775 | -0.7998 |
| Micromomyidae + 1 | 3.3432 | 2.2748 | 4.4233 | -0.9496 | -1.0972 | -0.8026 |
| Crown Primates (= Euprimates) | 4.0225 | 3.0383 | 4.9970 | -0.8861 | -1.0194 | -0.7490 |
| Crown Anthropoidea | 5.7591 | 4.7486 | 6.7639 | -0.8638 | -1.0028 | -0.7274 |
| Eosimiidae + crown Anthropoidea (2) | 4.5118 | 3.8674 | 5.1465 | -0.7511 | -0.8299 | -0.6750 |
| Crown Haplorhini | 4.0087 | 3.0367 | 4.9804 | -0.6969 | -0.8330 | -0.5604 |
| Tarsiidae | 4.5041 | 3.3371 | 5.6324 | -0.3172 | -0.5048 | -0.1341 |
| Post-*Teilhardina* omomyiforms + 3 (4) | 4.0214 | 3.2165 | 4.8328 | -0.7015 | -0.8201 | -0.5837 |
| *Teilhardina* + 4 (= omomyiforms + crown haplorhines) | 3.9266 | 3.0546 | 4.8000 | -0.7245 | -0.8471 | -0.6009 |
| Adapiformes + Strepsirrhini | 5.4275 | 4.7220 | 6.1264 | -0.8650 | -0.9546 | -0.7750 |
| Notharctinae | 5.8898 | 5.2810 | 6.5185 | -0.8517 | -0.9308 | -0.7738 |
| Crown Strepsirrhini | 5.0866 | 4.0084 | 6.1636 | -0.8283 | -0.9748 | -0.6785 |

**Table S6.** Ancestral state reconstructions for Tree 5. Tree 5 is largely the same as Tree 1, but plesiadapiforms are placed as stem dermopterans following Beard (1993). See text for other details.

| **Clade** | Estimated ln body mass | | | Ln(distal length/total length) | | |
| --- | --- | --- | --- | --- | --- | --- |
|  | Phylogenetic scaling parameter δ, directional | | | No phylogenetic scaling parameter | | |
|  | **Mean** | **L 95% HPD** | **U 95% HPD** | **Mean** | **L 95% HPD** | **U 95% HPD** |
| Primatomorpha | 1.3164 | -2.3033 | 4.8006 | -0.9098 | -1.0902 | -0.7292 |
| Dermoptera + Paromomyidae (1) | 4.8880 | 4.1809 | 5.5967 | -1.0976 | -1.1685 | -1.0252 |
| Plesiadapoidea + 1 (2) | 4.7161 | 3.8262 | 5.6135 | -1.0878 | -1.1734 | -1.0026 |
| Micromomyidae + 2 | 4.2110 | 3.1121 | 5.2993 | -1.0867 | -1.1892 | -0.9861 |
| Crown Primates (=Euprimates) | 3.2531 | 0.9416 | 5.5752 | -0.8209 | -0.9925 | -0.6532 |
| Crown Haplorhini | 3.6848 | 1.9897 | 5.3741 | -0.7222 | -0.8726 | -0.5729 |
| Tarsiiformes | 4.1116 | 3.2835 | 4.9690 | -0.6426 | -0.7254 | -0.5601 |
| Crown Tarsiidae | 4.7596 | 3.6012 | 5.8857 | -0.3024 | -0.4747 | -0.1265 |
| Eosimiidae + Anthropoidea | 4.5169 | 3.8817 | 5.1438 | -0.7523 | -0.8273 | -0.6786 |
| Crown Anthropoidea | 5.8941 | 4.8456 | 6.9324 | -0.8639 | -0.9967 | -0.7314 |
| Adapiformes + Strepsirrhini | 5.4956 | 4.5838 | 6.4117 | -0.8564 | -0.9430 | -0.7678 |
| Notharctinae | 5.8881 | 5.1232 | 6.6597 | -0.8469 | -0.9228 | -0.7715 |
| Crown Strepsirrhini | 5.2518 | 3.9144 | 6.6224 | -0.8476 | -0.9869 | -0.7097 |

**Table S7.** Ancestral state reconstructions for Tree 6. Tree 6 is largely the same as Tree 1, but *Carpolestes* is placed as more closely related to euprimates than other plesiadapiforms, following Bloch and Boyer (2002). See text for other details.

| Clade | Estimated ln body mass | | | ln(distal length/total length) | | |
| --- | --- | --- | --- | --- | --- | --- |
|  | Phylogenetic scaling parameter κ, directional | | | No phylogenetic scaling parameter | | |
|  | **Mean** | **L 95% HPD** | **U 95% HPD** | **Mean** | **L 95% HPD** | **U 95% HPD** |
| Euarchonta root | 2.7374 | 1.3288 | 4.1455 | -0.9830 | -1.1574 | -0.8080 |
| Scandentia | 3.0067 | 1.1788 | 4.808 | -0.9754 | -1.2771 | -0.6745 |
| Primatomorpha | 3.2644 | 1.947 | 4.584 | -0.9842 | -1.1550 | -0.8129 |
| Carpolestidae + Primates (1) | 4.2321 | 3.2636 | 5.2088 | -0.9417 | -1.0698 | -0.8120 |
| Euprimateforms (Plesiadapidae + 1) | 4.1459 | 3.1691 | 5.1154 | -0.9682 | -1.0965 | -0.8385 |
| Plesiadapidae | 6.1847 | 5.6239 | 6.7503 | -1.1532 | -1.2196 | -1.0871 |
| Paromomyidae + Euprimateforms (2) | 3.8633 | 2.8536 | 4.8728 | -0.9819 | -1.1148 | -0.8489 |
| Micromomyidae + 2 | 3.5432 | 2.4566 | 4.6218 | -0.9888 | -1.1293 | -0.8491 |
| **Crown Primates (=Euprimates)** | 4.2959 | 3.2687 | 5.2938 | -0.9155 | -1.0462 | -0.7810 |
| **Crown Haplorhini** | 4.1728 | 3.1418 | 5.1993 | -0.7598 | -0.9023 | -0.6194 |
| Crown Anthropoidea | 5.8083 | 4.8141 | 6.8219 | -0.8648 | -0.9952 | -0.7349 |
| *Crown* *Platyrrhini* | 6.4948 | 5.4665 | 7.5165 | -0.9000 | -1.0359 | -0.7670 |
| *Crown* *Catarrhini* | 7.7851 | 6.5825 | 8.9783 | -1.0460 | -1.2168 | -0.8753 |
| Crown Anthropoidea + Parapithecoidea (3) | 5.553 | 4.6616 | 6.4453 | -0.8512 | -0.9674 | -0.7326 |
| Eosimiidae + 3 | 4.5401 | 3.9012 | 5.1687 | -0.7542 | -0.8284 | -0.6817 |
| *Tarsiiformes* | 4.2313 | 3.5743 | 4.8877 | -0.6494 | -0.7302 | -0.5689 |
| Microchoerinae + *Tarsius* (4) | 4.7994 | 3.7843 | 5.8329 | -0.4573 | -0.6047 | -0.3067 |
| Anaptomorphinae + 4 (5) | 4.2204 | 3.5369 | 4.9087 | -0.6073 | -0.6876 | -0.5275 |
| Washakiini + 5 | 4.2293 | 3.5505 | 4.9137 | -0.6275 | -0.7084 | -0.5452 |
| Omomyinae | 5.5104 | 4.8451 | 6.1747 | -0.6620 | -0.7439 | -0.5823 |
| **Crown Strepsirrhini** | 5.3039 | 4.235 | 6.3883 | -0.8533 | -0.9900 | -0.7170 |
| *Crown Lemuriformes* | 5.6994 | 4.3365 | 7.03 | -0.8634 | -1.0523 | -0.6760 |
| *Microcebus* + *Mirza* | 4.8703 | 3.7989 | 5.937 | -0.6245 | -0.7829 | -0.4613 |
| Crown Lemuridae | 7.2671 | 6.1563 | 8.3646 | -0.9182 | -1.0734 | -0.7641 |
| Crown Lemuriformes - *Daubentonia* | 6.4046 | 5.2418 | 7.5905 | -0.8883 | -1.0425 | -0.7341 |
| *Crown Lorisiformes* | 4.9737 | 3.7558 | 6.1891 | -0.7675 | -0.9444 | -0.5911 |
| Crown Galagidae | 5.2666 | 4.4568 | 6.0689 | -0.5904 | -0.6966 | -0.4811 |
| Galagidae - *Euoticus* | 5.1481 | 4.1566 | 6.1472 | -0.4199 | -0.5606 | -0.2768 |
| *Galago* + *Otolemur* | 5.5174 | 4.5638 | 6.4676 | -0.4085 | -0.5441 | -0.2721 |
| Crown Lorisidae | 4.9921 | 3.7493 | 6.2284 | -0.7970 | -0.9750 | -0.6140 |
| *Anchomomys* + crown Strepsirrhini (6) | 5.316 | 4.3478 | 6.2736 | -0.8553 | -0.9783 | -0.7318 |
| Adapidae + 6 (7) | 5.5069 | 4.6192 | 6.406 | -0.8710 | -0.9839 | -0.7558 |
| Asiadapinae (8) | 5.2918 | 4.8154 | 5.7652 | -0.9005 | -0.9542 | -0.8479 |
| Notharctinae (9) | 5.9351 | 5.3122 | 6.5513 | -0.8518 | -0.9265 | -0.7774 |
| 7 + 8 + 9 | 5.5035 | 4.7956 | 6.2245 | -0.8649 | -0.9507 | -0.7802 |
| Adapidae | 7.6913 | 7.0826 | 8.2919 | -1.1667 | -1.238 | -1.0967 |
